# The Promiscuous Profile of Complement Receptor 3 in Ligand Binding, Immune Modulation, and Pathophysiology

**DOI:** 10.3389/fimmu.2021.662164

**Published:** 2021-04-29

**Authors:** Christina Lamers, Carla Johanna Plüss, Daniel Ricklin

**Affiliations:** Molecular Pharmacy Unit, Department of Pharmaceutical Sciences, University of Basel, Basel, Switzerland

**Keywords:** complement, integrin, CR3 (CD11b/CD18), inflammation, autoimmune diseases, host defense

## Abstract

The β_2_-integrin receptor family has a broad spectrum of physiological functions ranging from leukocyte adhesion, cell migration, activation, and communication to the phagocytic uptake of cells and particles. Among the members of this family, complement receptor 3 (CR3; CD11b/CD18, Mac-1, α_M_β_2_) is particularly promiscuous in its functional profile and ligand selectivity. There are close to 100 reported structurally unrelated ligands for CR3, and while many ligands appear to cluster at the α_M_I domain, molecular details about binding modes remain largely elusive. The versatility of CR3 is reflected in its functional portfolio, which includes prominent roles in the removal of invaders and cell debris, induction of tolerance and synaptic pruning, and involvement in the pathogenesis of numerous autoimmune and chronic inflammatory pathologies. While CR3 is an interesting therapeutic target for immune modulation due to these known pathophysiological associations, drug development efforts are limited by concerns of potential interference with host defense functions and, most importantly, an insufficient molecular understanding of the interplay between ligand binding and functional impact. Here, we provide a systematic summary of the various interaction partners of CR3 with a focus on binding mechanisms and functional implications. We also discuss the roles of CR3 as an immune receptor in health and disease, as an activation marker in research and diagnostics, and as a therapeutic target.

## Integrins and CR3: Function Through Flexibility

Integrins are a group of diverse cell surface receptors that play key roles in cell adhesion, communication, activation, migration, and cellular uptake. They provide major molecular links between extracellular matrix components, adhesion molecules, and plasma proteins. As a phylogenetically ancient family of large glycoproteins with origins that can be traced back 750 million years, integrins coevolved with the development of specialized cells, tissue, and metabolic and host defense systems such as the complement system (1). Integrins transmit signals bidirectionally across the plasma membrane and participate in a wide range of processes, such as inflammation, tissue homeostasis, and angiogenesis. Dysregulation of integrin activity has been associated with various clinical conditions, including autoimmune, thrombotic, and vascular diseases and cancer metastasis. Extensive efforts have therefore been directed towards the development of integrin antagonists (2–4), but few have yielded clinically approved drugs. In general, integrins are considered poor drug targets due to their molecular complexity, functional versatility and, in some cases, ligand promiscuity of the integrin receptors.

**Figure 1:**
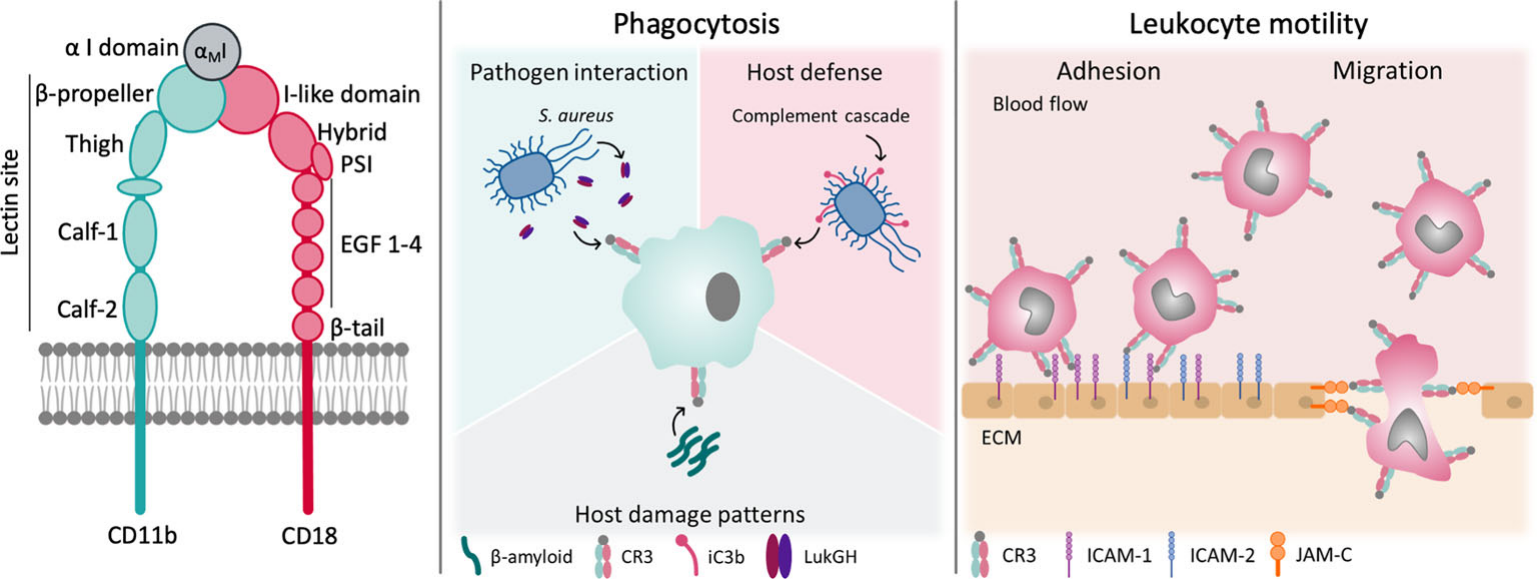
Structure and function of CR3. CR3 exerts a broad variety of functions in host-defense and leukocyte motility. Some of them are shown.

Few members of the integrin family illustrate the complexity of integrin-mediated interactions and functions as impressively as complement receptor 3 (CR3; CD11b/CD18, Mac-1, α_M_β_2_). This gives CR3 immense translational potential as a diagnostic marker and pharmacological target, which is reflected in a ligand repertoire approaching 100 molecules of natural and synthetic origin. CR3 is involved in leukocyte adhesion and migration, phagocytic elimination of pathogens, induction of both inflammatory and tolerogenic responses, and modulation of parallel or downstream host defense pathways (**Figure 1**). Despite numerous studies on CR3, aspects of its roles in health and disease and how it can be influenced on a molecular level remain elusive. Before unraveling the interactome and functional spectrum of CR3, we will provide a brief summary of the general molecular features of integrin receptors with a focus on the β_2_ family.

### Integrin Structure and Conformational States

Integrins are heterodimers that consist of two non-covalently associated α and β subunits, which are both type I transmembrane glycoproteins. The human integrin family encompasses 24 members, assembled from 18 different α and 8 different β subunits. In general, each subunit is defined by a large multidomain extracellular section, a transmembrane domain, and a typically short intracellular tail, the latter of which is important for integrin regulation and linkage to the actin cytoskeleton inside the cell (5). Whereas all α subunits share a β-propeller, a thigh, and two calf domains in their ectodomain, half of them, including CD11b of CR3, also have a globular I domain that is inserted in the β-propeller (6). These I domains contain a metal ion-dependent adhesion site (MIDAS), which coordinates divalent cations (e.g., Mg^2+^), and are the major binding area for integrin ligands (7). All β subunits contain an I-like domain with a similar fold to the α-I domains alongside a plexin-semaphorin-integrin (PSI), four epidermal growth factors (EGF), and a β-tail domain (**Figure 1**). These I-like domains contain a MIDAS, which is flanked by two adjacent metal coordination sites (termed AMIDAS and LIMBS/SyMBS) that bind Ca^2+^ and exert modulatory functions (5). The headpiece of integrins is formed by the β-propeller and I-domain of the α-subunit in contact with the I-like domain of β-subunit (5). In integrins that lack the I domain, the I-like domain makes extensive contacts with the β-propeller, and provides an interface for ligand binding (2).

**Figure 2:**
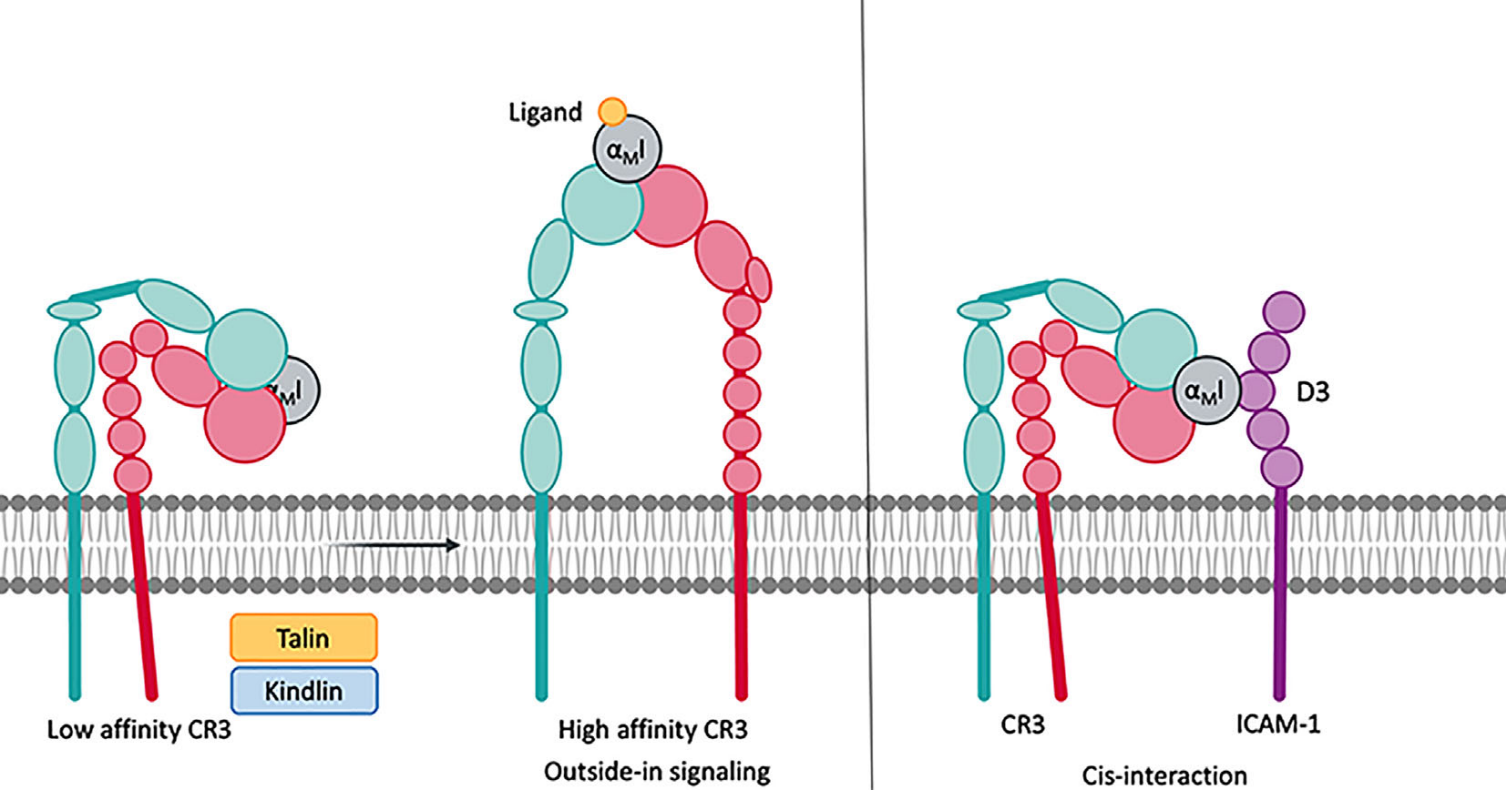
Integrins remain in a low affinity, bent-closed state. Cytoplasmic factors such as talin and kindlin connect the cytoplasmic tail of the integrins to the cytoskeleton. This leads to an extension of the extracellular domains with an open, high affinity, ligand accessible headpiece. CR3 is able to bind to protein on the same surface, which is termed cis-ligation/cis-interaction.

Integrin receptors derive their unparalleled functional versatility, including adhesion, “inside-out” and “outside-in” signaling, and/or ligand and particle uptake (8), from the unique composition of a compact headpiece that serves as an interaction platform, two flexible legs, and a transmembrane link to the cytoskeleton. The activity state of integrin receptors is defined by their three distinct conformations (**Figure 2**). In the activated state, both subunits are fully extended to expose over 4000 Å^2^ of solvent accessible surface (2). In the inactive state, the receptor is bent, and the globular head is kept close to the membrane surface, shielding the surface from solvent. In the intermediate state, the receptor is extended, but the cytoplasmatic tails are not separated, which seems to be induced by ligand binding to the bent conformation (5).

In the absence of stimuli, the integrins generally remain in the inactive, bent-close state, wherein the transmembrane domains of the two subunits are associated (9). Upon cell-stimulatory signals, such as cell surface receptors [e.g., toll-like receptor (TLR) or T-cell receptors], talin and kindlin are recruited to the cytoplasmatic tail of the integrins to connect them to the cytoskeleton (10). The resulting tensile force separates the intracellular tails and extends the extracellular domains to assume the active state (5, 11). This mode of signal transduction is referred to as “inside-out” signaling and may be further influenced by proteins that interact with the cytoplasmatic tail of the β-subunit. Mn^2+^ can induce integrin activation without separation of the cytoplasmatic tails, though bent and extended conformations with an open and closed headpiece coexist (8, 11). During leukocyte extravasation, P-selectin ligation induces the extended conformation with the closed headpiece, thereby enabling leukocyte rolling. Upon ICAM-1 ligation to integrins, this leukocyte rolling is decelerated. Finally, chemokine signaling *via* its GPCR receptors triggers leukocyte arrest, which opens the headpiece (12, 13).

The switch from the bent to extended conformation has profound consequences for ligand binding, which improves by several orders of magnitude (e.g., 4000-fold for cRGD binding to α_5_β_1_) (14). Affinity enhancements are often driven by a large reduction in ligand dissociation (15). This indicates that the extension of the receptor improves the accessibility of the headpiece and also induces conformational changes in the ligand binding domains (6). Indeed, this so-called “switchblade model” suggests a two-step activation process, during which extension of the legs is followed by a rearrangement of the binding area on the headpiece. For I domains, the extension leads to the downward axial displacement of the C-terminal helix to enhance ligand access and affects the position of the three loops that confine the MIDAS region.

While it is well established that ligand binding induces “outside-in” signal transduction, the underlying processes are less understood. Upon binding of extracellular ligands, integrin receptors can form clusters on the cell surface that extend from tenths of angstroms (microcluster) to > 200 nM (macrocluster) (16) and, by affecting binding avidity, enhance cell adhesion (17). Integrin clustering is observed on platelets (18), leukocytes (13), and, as patterned arrays, on primary neutrophils (12). Similar to “inside-out” signaling, conformational changes appear to play a critical role in “outside-in” signal transduction. Ligand-induced conformational propagation and receptor clustering trigger numerous intracellular signaling cascades after assembly of focal signaling complexes at the cytoplasmic face of the cell membrane, which may include kinases and adaptors. Nearly 60 proteins have been identified as constituents of this adhesome (19).

### β_2_ Integrins and Their Role in Health and Disease

The family of β_2_ integrins, comprising four members that all contain an α-I domain, are all found on leukocytes, yet each has a distinct expression pattern (20, 21). Whereas CD11a/CD18 (lymphocyte function-associate antigen 1 or LFA-1; α_L_β_2_) is found on all leukocytes, its expression is more prominent on lymphocytes. CD11b/CD18 (CR3) is the predominant integrin on neutrophils and is common on other myeloid cells, including macrophages, monocytes, eosinophils. It is also found on natural killer (NK) cells, mast cells, and B and T lymphocytes. While CD11c/CD18 (CR4, p150,95, α_X_β_2_) can be detected on NK, B, and T cells, it is predominantly expressed on myeloid dendritic cells, macrophages, and dendritic cells of the splenic white pulp and marginal zone. Finally, CD11d/CD18 (α_D_β_2_) is detected on most circulating monocytes and neutrophils, NK cells, and a small fraction of circulating T cells (22).

Despite their leukocyte-centered and partially overlapping expression profiles, the β_2_ integrin family has distinct ligand binding patterns. LFA-1 primarily binds to intercellular adhesion molecules (ICAM-1 to ICAM-5) and is critical for leukocyte trafficking by enabling firm adhesion to the endothelial layer and subsequent extravasation (6). Moreover, LFA-1 is an essential component of the immunological synapse between T cells and antigen-presenting cells (APC). LFA-1 also modulates the differentiation, survival, and activity of various lymphocyte subpopulations. Whereas CR3 and CR4 are also involved in leukocyte adhesion and migration, they are versatile in their interactions and functions, including phagocytosis of opsonized particles, podosome formation, and effector molecule enhancement (e.g., FcγR, uPAR, CD14). Among the most intriguing aspects of CR3 is its involvement in the removal of superfluous synapses during synaptic pruning (23). Comparatively, little is known about the functional spectrum of CD11d/CD18, which binds ICAM-3, VCAM-1, and matrix proteins (24). It must be noted that some of the functional aspects of β_2_ integrins have only been investigated in animal models and await confirmation in humans.

The tight involvement of β_2_ integrins in host defense and immune modulation (25–28) renders them a potential Achilles’ heel in the susceptibility for infectious, inflammatory, and/or autoimmune diseases. As the most prominent example of this, the autosomal recessive disorder leukocyte adhesion deficiency (LAD) presents with immunodeficiency and recurrent infections due to genetic mutations that encode the β_2_-subunit (25). Furthermore, β_2_ integrins are associated with the pathogenesis of leukocyte-mediated inflammation (during atherosclerosis or reperfusion injury), autoimmune disorders [e.g., systemic lupus erythematosus (SLE)], and dry eye disease. CR3 is an especially important regulator to balance the induction of tolerance, as it may inhibit the release of cytokines but may also generate an inflammation response. However, the regulation of this dual effect is not yet understood. It may be a combination of ligands, co-receptor ligation, or the pre-activation of the cell, and the cell types (e.g., macrophage *vs* dendritic cells) (28).

Several antagonistic compounds that target β_2_ integrins, such as small molecules and antibodies, have been in development as therapeutics (2–4, 26). However, few have reached the clinic. Therefore, it is critical to understand the functional and ligand binding spectrum of β_2_ integrins to increase access to therapeutic intervention.

## CR3: The Master of Ligand Versatility

When assessing the interactome of CR3, this β_2_ integrin family member is unique due to its distinct selectivity profile and its breadth of reported ligands. Whereas most integrins show a preference for RGD and/or LDV motifs (29, 30), these tripeptides do not seem to play a major role in CR3 recognition (31). CD11b has more than 100 reported ligands from soluble mediators, counterreceptors, and ECM components to microbial recognition and evasion molecules. The globular head of CR3, and in particular the α_M_I domain (7), is regarded as the main binding site, with some ligands that also engage with the β-propeller (2) of the α subunit or sites on the β_2_ domain. A lectin domain located on the C-terminus of the α subunit has been proposed but is poorly characterized. Finally, the glycosylation pattern of CR3 might account for binding to other receptors such as DC-SIGN (32), which renders CR3 a ligand itself. The quantity and promiscuity of ligands might be explained by the complexity of molecular interactions of CR3, which include multiple binding sites on the heterodimer and within the α_M_I domain and the glycosylation of CR3.

**Figure 3:**
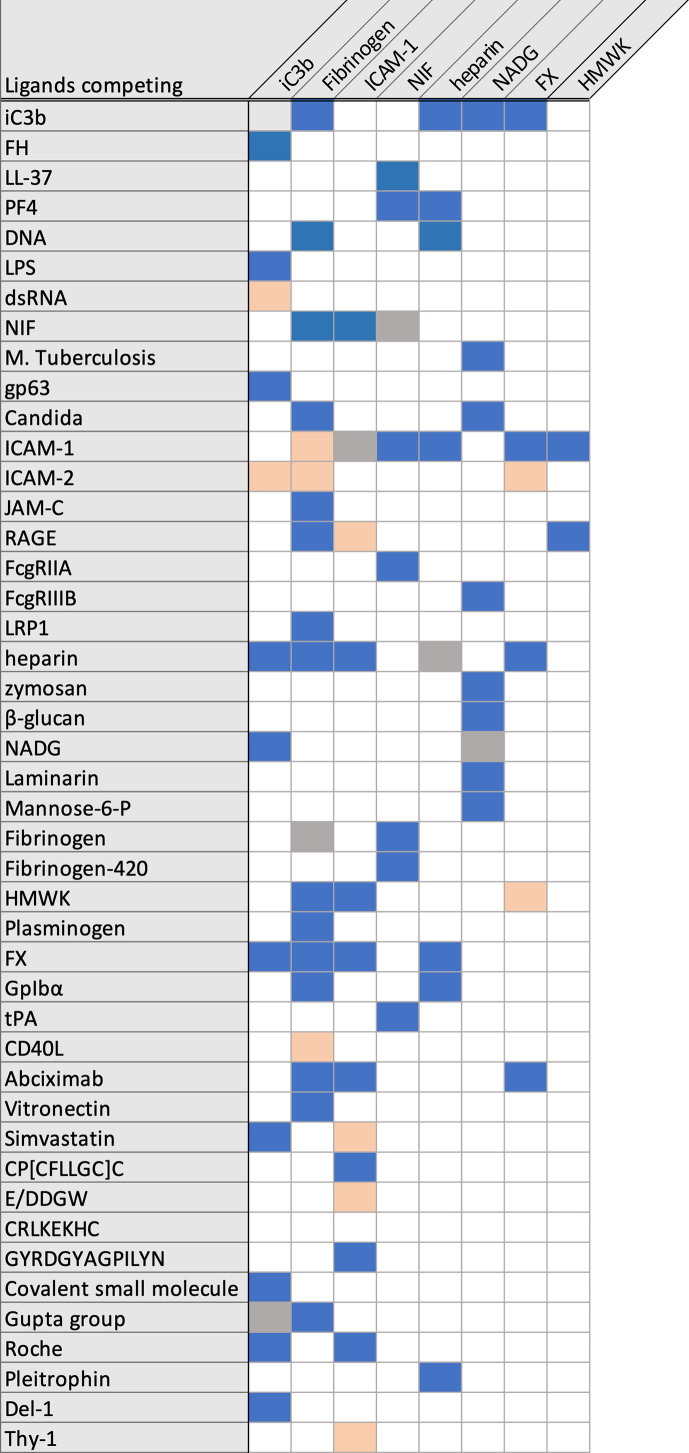
Overview over reported competing/not competing CR3 ligands. Competing ligands are marked in blue, not competing ligands are marked in salmon.

For most of the reported ligands, binding to CR3 was demonstrated in interaction assays using CR3-expressing cells, the purified heterodimer, or recombinant α_M_I. The recombinant α_M_I is expressed as either a wildtype variant or with a I316G mutation that yields a high affinity variant, which has been replicated in a mouse knock-in model (33, 34). The binding site has been mapped through competitive experiments with anti-CD11b antibodies and known CR3 ligands (**Figure 3**), although allosteric effects impede the interpretation of these results (35). As an aside, abolished ligand binding in the presence of EDTA is often interpreted as a MIDAS-mediated interaction with α_M_I, though the function of the β_2_ subunit is equally dependent on divalent cations. Additionally, EDTA may affect the potential binding of lectins to CR3 glycans. Otherwise, the exact binding mode has not been well investigated; a specific interaction to the α_M_I domain was confirmed with biophysical methods, though for less than 20% of reported ligands. Even with recent advances in integrin structure determination, structural insight into ligand binding remains scarce, with only a few available crystal structures of the α_M_I domain in complex with ligands (19). Despite these challenges, the conformational change between open and closed I-domains has been elucidated (36). Advances in cryo-EM (37, 38) and small angle x-ray scattering (39) have provided insight into the global conformation and interaction of CR3. Furthermore, the functional impact of CR3 ligation has been investigated *in vivo* for a few ligands (**Supplementary Tables 1–9**). However, the results from rodent models should be interpreted cautiously as little is reported about differences in CR3 across species. For example, mouse and human CR3 protein sequences share 78% similarity (40, 41), but the LukGH ligand shows specificity for human CR3 and no affinity to mouse CR3 (41, 42). In light of the imprecise description and insufficient validation of CR3 ligation (**Supplementary Tables 1–9**), some aspects about the interactome of this receptor must be regarded with care. Clearly, more investigations are warranted to fully understand CR3 ligand interactions, signaling, and functional consequences.

### The I Domain as a Canonical Binding Site

**Figure 4:**
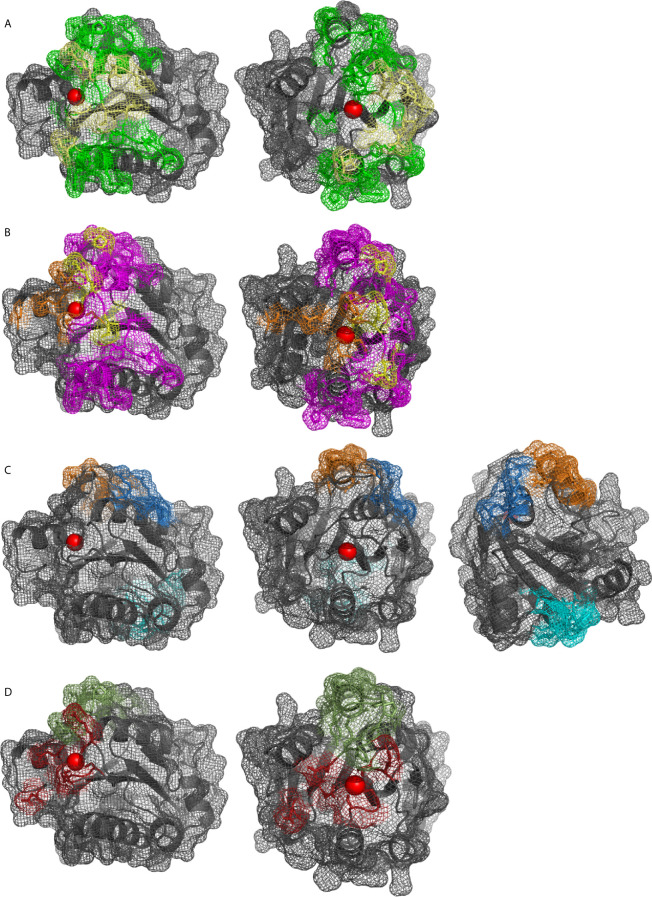
α_M_I domain (pdb: 1IDO) as cartoon and surface shown as mesh, Mg^2+^ in red. Binding sites of ligands reported by mutational, competition, structural or docking studies: **(A)** iC3b (green), C3d (yellow), **(B)** NIF (pink), LukGH (orange), (yellow for overlapping), **(C)** glucosamine (blue), FCgRII (orange), and CD40L and LRP1 (cyan), **(D)** GPIbα (ruby), fibrinogen (green).

The α_M_I domain, first crystallized in 1995 (43), coordinates the divalent metal ion with amino acids S142, S144, and D140 (α1 loop) of the MIDAS domain, and D242 (α5 loop) and T209 (α3-α4 loop). The sixth coordination site of the metal ion is free and can be filled by a carboxylic acid (Asp, Glu), either intrinsic partner, or by an extrinsic ligand (6, 43). The identification of multiple, overlapping binding sites in α_M_I (**Figure 4**) led to the introduction of a mosaic model of interaction areas (44). The βD-α5 loop and α5-helix (K245-A261) within the alphaMI domain are exclusive to CD11b in comparison to CD11a, that have been associated with the recognition of several CR3 ligands (45–47). Several groups have tried to identify a common binding motif for ligand interactions with the α_M_I domain, which led to the proposal of two opposite minimal binding sequences. Vorup-Jensen et al. postulated that a single carboxyl group, which coordinates with the divalent cation of the MIDAS, may define the relevant structural feature for ligand binding, as shown for simvastatin (48). While no specific pattern has been postulated, there are likely more amino acid contacts surrounding the MIDAS. However, this minimal binding feature would not apply to ligands that do not bind the MIDAS (e.g., CD40L, **Figure 4**). Alternatively, Podolnikova et al. proposed a minimal binding sequence of a basic amino acid surrounded by lipophilic ones such as HyBHy, HyHyBHy, HyBHyHy, and HyHyBHyHy (where Hy represents any hydrophobic residue and B is arginine or lysine) (49).

### The Lectin Site

A binding site was identified on the α-subunit of CR3 and termed the “lectin domain”, which interacts with carbohydrate structures and is unique among integrins. This site is distinct from the binding areas at the globular head and was mapped C-terminally to the α_M_I domain, which comprises the broad stretch of amino acids 400–1092 on the leg of CD11b (**Figure 1**) (50, 51). Ligation of the lectin site is suggested to induce a primed state of CR3 (52), which induces phosphorylation of the CD18 cytoplasmatic tail by protein kinase C (53), without increasing CR3 surface-expression. This process leads to a magnesium-dependent conformational change of the α_M_I domain and exposes the CBRM1/5 activation epitope. However, the epitope of the monoclonal antibody (mAb) 24 remains unexposed, which would indicate high affinity binding for ICAM-1 (54).

The simultaneous recognition of ligands *via* the lectin and α_M_I domains induces distinct responses. For example, co-ligation of CR3 by microbial β-glucan (lectin site ligand) and iC3b (α_M_I ligand) triggers phagocytosis and degranulation, while ligation of iC3b on erythrocytes or tumor cells in the absence of lectin site-ligation induced no cytotoxicity (55). Furthermore, β-glucans by themselves can induce ROS production and respiratory burst, whereas CR3 co-ligation by β-glucan and fibrinogen is required to release neutrophil extracellular traps (NET) (56).

The binding specificity of the lectin domain seems rather broad and includes polysaccharides containing mannose, N-acetyl-D-glucosamine (NADG), and glucose (51, 57). Ligands that bind CR3 at least partially *via* the lectin domain are microbial cell wall polysaccharides, such as zymosan, β-glucan,and laminarin (57) as well as glycans found in bacterial pili and fungal hyphae. In contrast, dextran and α-mannan do not bind.

### Cis-Ligation

Cis-ligation is the simultaneous interaction of proteins on the same cell surface, as opposed to trans-ligation, which involves another cell. In addition to trans-ligation, CR3 has been shown to interact with several membrane proteins *via* cis-ligation, which is typically mediated by the lectin and/or the α_M_I domains (see above). Depending on the ligation site, cis-ligation may result in either amplification of the response or in negative regulation. For example, cis-ligation of the lectin site leads to the formation of multi-protein complexes, which may substantially enhance effector function, such as phagocytosis. Conversely, cis-ligation by α_M_I domain interactions seems to trap the integrin in a bent, and thus inactive, conformation and impair receptor functions. Protein-complexes with increased activity after cis-ligation with CR3 include FCγRIII (58, 59), uPAR (60), tPA (61), and SLAMF7 (62). Negative regulation upon cis-ligation was reported for ICAM-1 (13), IL-13Rα1 (63, 64), FcγRIIa (65), and CD22 (66).

## CR3 Modulating Leukocyte Function

As major leukocyte receptors, β_2_ integrins in general and CR3 in particular profoundly shape the cellular immune response through numerous interactions. The repertoire of CR3 ligands associated with leukocyte function are structurally diverse but can still be clustered based on their function (**Table 1** and **Supplementary Table 1**). CR3 is a major receptor for the phagocytosis of opsonized particles. As such, CR3 plays an important role in host defense and the removal of cell debris and apoptotic cells. At the same time, pathogens use this integrin for host/cell invasion and immune evasion. Moreover, CR3 is involved in leukocyte migration, especially during inflammation, with several ligands reported as counterreceptors for this purpose. Finally, the crosstalk of CR3 with proteins of the hemostatic systems plays a pivotal role in thrombosis and links hemostasis to inflammation.

**Table 1:** Host defense mechanisms.

Ligand	Site on CR3	Site on ligand	Function	
iC3b	α_M_I (MIDAS), β_2_ (DXSXS)	TED, C345c, MG7	Phagocytosis, induction of tolerance	(7, 38, 39, 45, 47, 67–82)
C3d/C3dg	α_M_I (MIDAS)	TED	Phagocytosis	(77, 81)
C3(H_2_O) iC3	–	–	Tethering of PMN and platelets	(83)
FH	overlapping with iC3b	CCP6-7, CCP18-20	Neutrophil activation, PMN polarization, H_2_O_2_ and lactoferrin release	(84–86)
Del-1	Competition with iC3b		Impairing binding of iC3b to CR3 and reduced phagocytosis	(87)
LL-37	α_M_I, competition with NIF	Residues 18-37	Increasing phagocytosis of bacteria	(88, 89)
Platelet factor 4	α_M_I	Residues 12-26, 57-70, 58-66, 61-69	Neutrophil activation, phagocytosis, integrin clustering	(90)
FcγRIIA	α_M_I (E253-R261), NIF overlapping, divalent cation dependent, cis ligation	Silalic acid (N64, N145)	Antibody-dependent cytotoxicity, migration, immunological synapse formation	(65, 91, 92)
FcγRIIIB (CD16)	Lectin domain	–	Synergistic respiratory burst upon coligation of IgG and iC3b	(65, 91, 92)
SLAMF7	–	–	Phagocytosis	(62)

CR3 was shown to regulate neutrophil apoptosis, which leads to an enhanced accumulation of CD11b^-/-^ neutrophils (93). Mature neutrophils are terminally differentiated cells with a short circulation half-life. They are cleared from circulation by spontaneous apoptosis and subsequent phagocytosis by resident macrophages in the spleen, bone marrow, and Kupffer cells in the liver (94). A hallmark of inflammatory resolution is the engulfment of apoptotic neutrophils by macrophages (efferocytosis). Inflammatory stimuli (e.g., G-CSF, TNFα, IL-6, LTB4, C5a or LPS) and endothelial transmigration (95) *via* adhesion to ICAM-1 or fibrinogen (96) prolong the life span of neutrophils. Conversely, high levels of TNFα accelerate apoptosis (97). Complement-opsonized targets induce apoptosis of polymorphonuclear leukocytes (PMNs) *via* CR3 (98, 99), while pro-survival signals can be triggered by CR3 clustering-dependent binding to fibrinogen and plasminogen (95, 96, 100). This clustering is induced by ligands that bind both sub-domains of CR3, but not those that solely bind the α_M_I domain (e.g., NIF or P1) (101). On a molecular level, the pro-survival signal *via* activation of Akt is dependent on specific ligands rather than integrin activation. This effect was shown with α_M_I mutants locked in the active state, which failed to induce the pro-survival signal (101).

### Phagocytosis—CR3-Mediated Danger Sensing and Elimination

#### Host Defense Mechanisms

CR3 is a major regulator in host defense and tissue homeostasis due to its central role in the removal of invaders, damaged cells, and protein aggregates by mediating phagocytosis (67, 68). Phagocytosis *via* CR3 largely depends on prior opsonization of surfaces by the complement system and can result in pro-inflammatory signaling, induce tolerance with reduced IL-12 levels, and upregulate tolerogenic IL-10 and TGFβ (102). The complement cascade is initiated upon recognition of antibody clusters or non-self-signatures on targeted cells, and this initiation cleaves the plasma protein C3 to generate an anaphylatoxin (C3a) and the opsonic C3b fragment that is deposited at the site of activation. C3b can form C3 convertase complexes that activate more C3. This fuels an amplification loop that culminates in the cleavage of C5 with subsequent formation of the inflammatory mediator C5a and lytic membrane attack complexes. At the same time, complement regulators degrade C3b into the fragments iC3b and C3dg, which do not participate in amplification, though do mediate immune functions (103).

Among these fragments, **iC3b** is considered the main ligand recognized by CR3 that leads to phagocytosis (67, 68), and the interaction was shown to be dependent on divalent cations (69). To determine the CR3-iC3b binding interaction, investigations identified multiple discontinuous sites on CR3 (70, 104). On the α_M_I domain, this included areas surrounding the MIDAS (i.e., βA-α1, α3-α4, βD-α5, and βE-α6 loops) (7, 71, 72). The fourth blade of the β-propeller in CD11b (73) and a conserved amino acid sequence (DXSXS) in CD18 (72, 74, 75) also participate in the interaction. Consistent with these results, transgenic cells that express CR3 and lack α_M_I can still bind to iC3b, albeit with lower affinity (76).

On host cells that express complement receptor 1 (CR1, CD35) as cofactor, iC3b is further degraded by factor I to surface-tethered C3dg and C3d and soluble C3c. While C3b and C3c have weak affinities for CR3 and CR4, comparable to the affinity of a sole acidic side chain that interacts with the MIDAS, **C3d** and **C3dg** display strong affinity for CR3 and weak for CR4 (77). While the distinct selectivities of the related complement receptors was unexpected, they show that differential patterns of complement opsonization/regulation and CR3/CR4 expression may fine-tune an immune response.

In addition to its cleavage by convertases, C3 can also assume an activated state through low-level hydrolysis in circulation or upon contact with various surfaces. The resulting **C3(H_2_O)** is structurally similar to C3b and can be degraded to **iC3**, which largely corresponds to iC3b. Both C3(H_2_O) and iC3 have been detected on activated platelets and facilitate the tethering of platelets to PMNs (83).

**Factor H (FH)**, a soluble complement regulator, was reported to adhere to PMNs in a divalent cation and CR3-dependent manner, which leads to activation and polarization of adhered PMN with increased release of H_2_O_2_ and lactorferrin (84). FH domains CCP6-7 and CCP18-20 were identified as interaction sites for CR3, and the observed competition with iC3b suggested overlapping binding sites on the receptor (85). FH, and CFHR1 that contains domains homologous to the binding areas of FH and may therefore act as CR3 ligand, can bind simultaneously to neutrophils and *Candida albicans*. Consequently, this enhances neutrophil adherence and pathogen killing (86).

**Developmental endothelial locus-1** (Del-1) is a matrix glycoprotein expressed and secreted by endothelial cells. It has been shown to behave as an antagonist for CR3-mediated phagocytosis through competition with iC3b (87). Del-1 is also able to inhibit the LFA-1:ICAM-1 interaction, which reduces inflammatory cell recruitment and IL-17 induction (105).

Upon recognition of danger signals, leukocytes secrete certain host defense molecules that also bind to CR3. **LL-37**, an endogenous antimicrobial peptide, is secreted by a wide variety of cells (e.g., neutrophils, NK, mast cells, B and T cells, and epithelial cells) and binds to the α_M_I domain of CR3. LL-37 also has a high affinity for bacterial cells, which increases phagocytosis (88, 89). **Platelet factor 4** (PF4, also known as CXCL4) is a cationic protein secreted from α-granules of activated platelets to activate neutrophils, augment phagocytosis, and induce integrin clustering in a CR3-dependent manner (90). Direct binding of PF4 to isolated α_M_I domains has been observed with biolayer inerferometry (90).

Members of the **Fc receptor** family bind immunoglobulins with distinct specificities and functional consequences, which include phagocytic uptake of antibody-coated cells and particles. Simultaneous phagocytosis of iC3b-opsonized and IgG-labeled particles leads to a synergistic respiratory burst (106). Additionally, CR3 is needed for optimal phagocytosis *via* FcγR (107, 108). The interplay between CR3 and FcγR is considered critical for immunological defense mechanisms. For example, the ligation of FcγRIIa is necessary for antibody-dependent cellular cytotoxicity (ADCC) and critical for FcγRIIa-mediated cell-spreading, migration (91, 109), and immunological synapse formation (110). Co-ligation of CR3 and FcγRIII leads to an association of FcγRII with the actin cytoskeleton and subsequent phosphorylation that induces a respiratory burst (92). A soluble form of FcγRIII (sCD16) is shed from cell surfaces and triggers cell activation by interacting with CR3 and CR4, which leads to “outside-in” signaling and cytokine production (111). For FcγRIIa (CD32a), cis-ligation to the CR3 α_M_I domain inhibits neutrophil recruitment, which is absent in the SLE-associated SNP rs1143679 (R77H) (65).

The direct interaction of FcγRIII with CR3 was elucidated by resonance energy transfer studies and microscopy (112). In competition experiments with NADG, the relevant binding site was mapped to the lectin site of CR3. In contrast, the interaction with FcγRIIa was insensitive to NADG, and binding studies with recombinant α_M_I confirmed an overlapping binding site to neutrophil inhibitory factor (NIF) (91). A detailed investigation of the binding mode showed that sialic acids on FcγRIIa glycans (attached to N64 and N145) are responsible for the α_M_I interaction, as treatment with neuraminidase diminished binding (65).

**SLAMF7** (CD319) is a robust marker of malignant plasma cells in multiple myeloma and was shown to be responsible for the phagocytosis of cells after disruption of the tumor surveillance checkpoint CD47-SIRPα. The colocalization of SLAMF7 with CR3 in a cis interaction was shown with co-immunoprecipitation studies and confocal microscopy. Furthermore, phagocytosis was inhibited with anti-CD11b mAbs and CD11b knock-out (62).

#### Direct Pathogen Interactions

Although CR3-dependent phagocytosis is typically mediated by complement and/or antibody opsonization, several pathogens are directly recognized by CR3, which facilitates phagocytosis and induces an antimicrobial response. However, some intracellular pathogens exploit CR3 as an effective entry port to invade cells. Such pathogen interactions with complement integrin receptors have been reviewed recently (113), so in the following sections, we focus on the molecular determinant and involved ligands (**Tables 2**, **3** and **Supplementary Tables 2, 3**) that enable pathogen recognition by CR3.

**Table 2:** Defense against pathogens.

Ligand	Site on CR3	Site on ligand	Function	
neutrophil inhibitor factor (NIF)	α_M_I (MIDAS), Competition with ICAM-1, fibrinogen	–	Blocking adhesion of neutrophils to vascular endothelium	(44, 75, 114–116)
LPS	not iC3b, divalent cation dependent	Lipid A	–	(78, 117)
*Klebsiella pneumonia*	Divalent cation dependent	LPS like	–	(118)
Zymosan	Lectin domain	–	Induces phagocytosis and with co-ligation to iC3b respiratory burst	(119)
β-glucan	Lectin domain	–	Activates integrin, induces phagocytosis and with coligation to iC3b respiratory burst; coligation to fibrinogen induces NETosis	(51, 54, 120, 121)
Double-stranded RNA	–	–	NOX2 activation, production of ROS, TNF-α, IL-12p40, IFN-β	(122)
Laminarin	Competing with N-acetyl-D-glucosamine	–	–	(57)
N-Acetyl-D-glucosamine	Lectin domain, competing with FcγRIIIB, iC3b, Laminarin, Glucos-6-P	–	–	(51, 57)
Mannose-6-P	Competing with N-acetyl-D-glucosamine	–	–	(51, 57)

**Table 3:** Pathogen evasion.

Ligand	Site on CR3	Site on ligand	Function	
*Staphylococcus aureus* Leukocidin GH	α_M_I (MIDAS)	LukH (main) & LukG	Pore formation, virulence	(41, 42)
*Streptococcus pneumoniae* - Pneumolysin	sLe^x^ on α_M_I	–	Pore formation	(123)
*Mycobacterium tuberculosis* and *smegmatis*	α_M_I (not iC3b site), C-terminal for *M. tuberculosis*, competes with laminarin and NADG	–	Binding and internalization of *M. tuberculosis*	(124)
*Neisseria gonorrhoeae*	Activated α_M_I, lectin domain, cooperative with FH	Pilus glycan	Host evasion: internalization without inflammation	(85, 125, 126)
Group B Streptococci	–	–	Phagocytosis	(127)
*Porphyromonas gingivalis* - Fimbrillin	–	–	IL-12 regulation, pathogenicity	(128, 129)
*Bordetella pertussis*	Binding of CyaA Ca^2+^ dependent, not Mg^2+^ dependent, → binding is α_M_I-domain independent?	–	Reduced expression of IL-12, macrophage adhesion	(130–134)
*Bacillus anthracis* - BclA	–	–	Spore uptake	(135)
*Streptococcus pneumoniae* - RrgA	α_M_I	–	Increased phagocytosis, virulence	(136)
*Franciscellas tularensis*	–	–	C3 opsonized results in limited inflammasome priming and pro-inflammatory cytokine production	(137)
*Borrelia burgdorferi –* OspA, OspB	Not overlapping to iC3b	–	–	(138)
*Leishmania* sp. - gp63	–	Residues 365-386, 252-255	–	(139–141)
HIV-1	–	–	–	(142, 143)
Herpes simplex 2	Opsonized with iC3b and without	–	Opsonized HSV2 increased infection of DC	(144)
Hantavirus	In competition to heparin		Increased virulence by NETosis causing severe renal and pulmonary pathology	(145)
*Candida albicans*	Competed by vitronectin, fibrinogen (α_M_I-domain) and NADG, β-glucan (lectin)	beta-glucan	candida killing by co-ligation with FH	(146, 147)
*Blastomyces dermatitis*	Via same binding site like LPS, divalent cation dependent	probably *via* the WI-1 surface protein	Increased phagocytosis, virulence	(148)
*Histoplasma capsulatum*	Divalent cation dependent, not *via* lectin binding site	–	Increased phagocytosis, virulence, induction of ROS	(149)

##### Defense Against Pathogens

**Neutrophil inhibitory factor** (NIF), a glycoprotein of hookworms, blocks adhesion of neutrophils to the vascular endothelium (150) by binding CR3 with high affinity (low nM range) and long target residence (t_½_ ~ 8 h) (114). Interestingly, the CR3-NIF interaction is independent of neutrophil activation (114). The binding site is located within the α_M_I domain (115) with several reported contact areas (75, 114). NIF binding is cation-dependent (70) and competes with fibrinogen and ICAM-1 (116), but not with FX (44). Reports on competitive binding for iC3b are contradictory (44, 116), which may be attributed to different experimental setups (i.e., recombinant α_M_I *vs* the full heterodimer). A homolog-scanning mutagenesis approach highlighted the importance of contact areas that surround MIDAS and identified the α_X_I domain of CR4 as a potent receptor for NIF (116).

** **** *Escherichia coli *binds to macrophages even in the absence of complement components. This might be possible due to its surface glycoprotein, lipopolysaccharide (**LPS**), which was identified as a binding partner to β_2_ integrins. The lipid A part of LPS appears to play a critical role in this binding interaction, since polymyxin B sulfate can block binding (117). The binding site on CD11b was shown to be distinct from iC3b binding, but this was not further characterized (78). The acylpolygalactosides of ***Klebsiella***, which consist of a poly(1, 3)galactose chain, glucosamine disaccharide, and lipid βOH myristates, are similar in structure to LPS. These structures mediate Ca^2+^- and Mg^2+^-dependent binding to monocytes, with involvement from both CD14 and CR3 (118).

**Zymosan A**, a preparation of cell wall glucans from *Saccharomyces cerevisiae*, is a known activator of the complement system, but also directly activates macrophages and induces phagocytosis (119, 151), with **β-glucan** as the responsible component. β-glucans are high-molecular-weight polysaccharides comprised of β-D-glucose and are typically found in the cell walls of bacteria, fungi, and plants [**laminarin** (57)]. β-glucans can activate macrophages that mediate phagocytosis and respiratory burst (51) *via* CR3 (119). They have shown remarkable activity as an immune-modulatory compound in animal models and clinical trials (152). Imprime PGG (see* section 3.4*) is a β-glucan derivative, which has been investigated in clinical trials in combination with therapeutic antibodies for oncotherapy (153–156).

β-glucans do not bind to the canonical binding sites, such as the α_M_I domain or the β_2_ globular head, but rather to the lectin domain (50, 54). Monosaccharides such as mannose, galactose, fucose, and glucose can only compete with β-glucan at very high concentrations (>200 mM), while **NADG**, α- and β-methylmannoside, α- and β-methylglucoside, and **mannose-6-P** are stronger competitors (51, 57). Interestingly, distinct responses between soluble β-glucans (sbglu) and immobilized β-glucans have been reported (52). Binding of sbglu to CR3 yields similar responses as induced “inside-out” signaling (i.e., increased CR3 expression, conformational change to an extended state, increased affinity to fibrinogen), albeit to a lesser extent. Remarkably, the extension induced by sbglu results in an intermediate conformation that leads to cellular activation, which can be detected by the phosphorylation of proteins associated with transcriptional regulation, mRNA processing, and alternative splicing. Another study suggested that sbglu binds CR3 indirectly *via* opsonization with iC3b (120). One possible explanation for those conflicting results might be the use of different species between studies, since dectin-1 is the predominant receptor for β-glucan phagocytosis in mice, while CR3 is solely responsible for β-glucan signaling in humans (121).

**Double stranded RNA** (dsRNA), as a result of viral infection, is bound and internalized by CR3 *via* an unspecified molecular mechanism, which leads to increased NOX2 activity, ROS production, and elevated levels of proinflammatory cytokines, such as TNF-α and IFN-β, as shown in a mouse model (122).

##### Pathogen Evasion

**Leukocidin** is a pore-forming toxin from ***Staphylococcus aureus*** that lyses phagocytic cells, such as neutrophils, monocytes, and macrophages. CD11b and CD18 are both targets of this bi-component cytotoxin, which is composed of LukG and LukH subunits (41) that were shown *via* crystal structure to form a heterotrimer with CR3. The heterotrimer oligomerizes into a β-barrel pore, which is inserted into the cell membrane through bending of the β_2_ integrin (42). LukGH establishes extensive polar contacts and salt bridges to the α_M_I domain, including the MIDAS, as well as lipophilic interactions (42). Cytolysins are able to form pores in a similar way as leucocidin. For example, **pneumolysin** from *Staphylococcus pneumoniae* interacts with CR3 with high affinity (K_D_ ~ 7 nM) *via* the sialyl-Lewis^X^ of the α_M_I domain (123).

***Mycobacterium tuberculosis***, a bacterium that colonizes intracellularly in mononuclear phagocytes, is another pathogen that uses CR3. Binding and internalization of *M. tuberculosis* is mediated by iC3b-opsonization and through direct binding to CR3 (157, 158). The direct binding was mapped to the α_M_I domain, distinct from the iC3b binding site, and to the C-terminus, which is likely the lectin domain (124). *Mycobacterium* strains with capsular polysaccharides composed of glucose, arabinose, and mannose (e.g., *M. tuberculosis*) involve the lectin domain in binding, whereas strains with low densities of phosphatidylinositol mannoside (e.g., *M. smegmatis*) are primarily opsonized by iC3b and phagocytized (159).

***Neisseria gonorrhoeae*** exploits CR3 for internalization without inducing inflammation *via* several mechanisms (160). iC3b-opsonized gonococci and the outer membrane proteins of porins and pili are all known ligands of the α_M_I domain (125, 161). Additionally, a cooperative mechanism of FH-mediated bridging between gonococci and CR3 has been postulated (85). The C-terminal CCP18-20 binds to CR3 when the FH domains CCP6-20 assume the appropriate spatial orientation, while CCP6-10 and CCP18-20 are able to adhere to gonococci (85). In addition, glycans displayed on the pili contribute to binding CR3 in its closed conformation, thereby activating it (126), which indicates an involvement of the lectin domain.

Additional pathogens that bind to CR3, mostly *via* membrane-bound glycoproteins, and can increase virulence are **group B streptococci** (127), ***Porphyromonas gingivalis*** (128, 129), ***Bordetella pertussis*** (130–133), ***Bacillus anthracis*** (162), ***Streptococcus pneumoniae*** (136), ***Francisella tularensis*** (137), ***Borellia burgdorferi*** (138), and the parasite ***Leishmania*** (139, 163). CR3-mediated binding induces phagocytosis, which often results in decreased IL-12 levels (129, 134) and inhibition of inflammasome activation (137), which leads to pathogen evasion from immune surveillance. For most of these pathogens, binding to CR3 was reported without knowledge of the exact binding site. In the case of *Leishmania*, its glycoprotein gp63 was shown to interact with the α_M_I domain (140, 141) and compete with iC3b binding, which indicates an overlapping binding site.

Some viruses hijack CR3 for host cell entry and immune evasion. For example, non-opsonized and iC3b-opsonized **HIV-1** both use CR3 for internalization by monocytes and immature dendritic cells for effective transfer to CD4^+^ T cells (142). Complement-mediated opsonization of HIV-1 leads to enhanced infection due to decreased anti-viral and anti-inflammatory responses (143). Similarly, opsonized **herpes simplex virus 2** is internalized *via* CR3, which leads to increased infection of dendritic cells (144). Other viruses, such as **hantavirus**, use CR3 as an entry receptor but induce strong NETosis (145), thereby harming the host.

Large granular lymphocytes adhere to the hyphae (polymeric β-glucan structures) of ***C. albicans*** *via* CR3 and inhibit their growth (146, 164). The molecular binding site on CR3 was mapped to the α_M_I domain, although the lectin domain also seems to play a role, since N-acetyl-D-glucosamine, D-mannose, and β-glucan compete with binding. Other fungi, such as ***Blastomyces dermatitis*** (148) and ***Histoplasma capsulatum*** (149), also adhere to CR3.

#### Recognition of Host Damage Patterns

**Damage-associated molecular patterns (DAMPs)** typically refer to changes in cell-surface expression profiles upon host cell damage or apoptosis, cellular debris, and aggregated proteins. Such cells and debris are typically removed by phagocytes to maintain homeostasis. Similar to its direct recognition of pathogens, CR3 can directly bind to various DAMP ligands (**Table 4** and **Supplementary Table 4**) and thereby contribute to homeostasis.

**Table 4:** Recognition of host damage patterns.

Ligand	Site on CR3	Site on ligand	Function	
Albumin (also denatured), Ovalbumin	–	Unfolded parts, flexible loops containing acidic residues	–	(165–167)
DNA	Competition with heparin and fibrinogen	–	ROS production	(168)
Myelin basic protein (MBP), galitamer acetate (GA)	α_M_I (MIDAS)		Phagocytosis of denatured myelin	(169–171)
β-amyloid	–	–	NO release, decreased phagocytic activity, increased β-amyloid degradation	(172–176)
α-synuclein	–		Translocation of p47^phox^ → NOX2 activation, ROS production, CR3 involved in synucleopathies?	(177–179)
CD157	–	–	Neuroinflammation	(180)
2,5-Hexanedione	–	–	Translocation of p47^phox^, NOX2 activation, ROS production	(181)
Diesel exhaust	–	–	NOX2 activation, ROS production	(182)
HMGB1 (amphotherin)	–	–	Increases TNF-α, IL-1β and NO formation → neurodegeneration	(183)

Synthetic **DNA** oligodeoxynucleotides are bound and internalized by CR3, which induces production of ROS in TNF-α/fMLP-stimulated PMNs (168). Furthermore, CR3 can recognize many denatured proteins. For example, **denatured BSA** and **ovalbumin** bind CR3 on neutrophils (165). Albumin immobilized on polystyrene and perfluorinated surfaces is a ligand for CR3, which indicates that BSA is not a suitable blocking agent for *in vitro* assays with leukocytes and CR3 (166, 167).

**Myelin basic protein (MBP)** is a potential autoantigen in multiple sclerosis (MS), and a mouse model of MS showed a correlation between MBP binding to CR3 and experimental autoimmune encephalitis (EAE) (169, 170, 184). CR3-deficient mice are protected from developing symptoms in an EAE model (184). Mimicking unfolded MBP, the mixture of basic peptides known as glatiramer acetate (GA), is an active ingredient of an MS therapeutic that competes with MBP binding by also binding the CR3 α_M_I domain (171). Finally, direct binding of MBP to CR3 is dependent on divalent cations (Ca^2+^, Mg^2+^, and Mn^2+^) (171).

Aggregated** β-amyloid** is associated with Alzheimer’s disease (172) as well as increased CR3 expression. In mouse models, β-amyloid binding to CR3 increased NO release, which led to neurotoxicity, but also the induction of phagocytosis and the degradation of β-amyloid (173–175). These data indicate a complex or dual role of CR3 in pathogenesis. Similarly, in the neurodegenerative Parkinson’s disease (PD),** α-synuclein** aggregates are associated with the induction of neurotoxicity by activation of microglia that leads to NOX2 (NADPH oxidase) activation and ROS production *via* Src-Erk- and Rho-dependent pathways (177, 178), where CD11b^-/-^ microglia are better protected (179). Another risk factor for PD, **CD157**, a member of the NADase/ADP-ribosyl cyclase family, can functionally associate with CR3 to potentially drive neuroinflammation (180). Similarly to α-synuclein, **2,5-hexanedione** (181) and **diesel exhaust particles** (182) induce dopaminergic neurodegeneration *via* activated rodent microglia, CR3-dependent NOX2 activation, and ROS production *via* the Src-Erk pathway. When found extracellularly, the intracellular, chromatin-binding protein **high mobility group box protein 1** (HMGB1, amphoterin), causes increases in TNFα, IL-1β, and NO secretion. In this process, the NFκB pathway activates NADPH oxidase, which induces progressive neurodegeneration in rodents. The direct interaction between HMGB1 and CR3 was shown by co-immunoprecipitation experiments (183), but the involved domains and mechanism of action remains unknown.

### CR3 as Modulator of Leukocyte Motility

#### Leukocyte Adhesion and Extravasation

A major role of β_2_ integrins is the mediation of leukocyte adhesion to endothelial or interstitial matrix with subsequent leukocyte migration and tissue inflitration. This process is essential for directing immune cells to sites of inflammation and is facilitated by β_2_ integrins that employ a set of ligands to control leukocyte motility (**Table 5** and **Supplementary Table 5**).

**Table 5:** Leukocyte adhesion and extravasation.

Ligand	Site on CR3	Site on ligand	Function	
ICAM-1 (CD54)	α_M_I, DXSXS in β_2_, not competing with fibrinogen	3. Ig domain	Leukodiapedesis – expression only in inflammatory sites	(185–189)
ICAM-2 (CD102)	α_M_I, not competing with iC3b, fibrinogen and FX	1. Ig domain	T cell aggregation, NK cell migration and cytotoxicity	(190, 191)
ICAM-4	Divalent cation dependent	Ig domains D1 and D2	–	(192)
JAM-C (junctional adhesion molecule)	α_M_I, competing with fibrinogen	–	Platelet-neutrophil interaction, transepithelial migration	(193, 194)
CD147 (Basigin)	–	–	–	(195)
RAGE (AGER)	α_M_I, competing with fibrinogen and HMWK	–	Neutrophil extravasation into peritoneum	(196)
Thy-1 (CD90)	α_M_I, not competing with ICAM-1	–	Neutrophil adhesion to endothelial cells, migration, accumulation in skin lesions	(197, 198)
SIRPα (signal regulatory protein α)	α_M_I	Ig1-2-3 ectodomain	Macrophage fusion, anti-phagocytosis signal	(199)
CD40L	α_M_I, distinct of fibrinogen	Distinct from CD40 and GPIIb/IIIa binding site	leukocyte recruitment in atherosclerosis	(200)
Myeloperoxidase	–	–	–	(201)
Azurocidin and Elastase	–	Catalytic domain	–	(202)
Pro-MMP-2, Pro-MMP-9	α_M_I	Catalytic domain	Suggested to be involved in neutrophil migration	(203, 204)
Pleiotrophin	α_M_I	Thrombospondin type-1 repeat domains	Macrophage migration, MAP kinase activation, phosphorylation of Erk1/2	(205)
Dynorphin A	α_M_I		Enhanced phagocytosis	(206)
DC-SIGN	Le^X^ on CD11b, binds only to CR3 on PMNs	CRD	DC maturation, cytokine production	(32)

ICAMs belong to the immunoglobulin superfamily and serve as primary counterreceptors in this process. They differ in expression pattern and cellular distribution, with ICAM-1 expressed at low levels on leukocytes, endothelial cells, keratinocytes, and fibroblasts and up-regulated upon inflammatory stimuli, such as cytokines. ICAM-2 is constitutively expressed by platelets, leukocytes, and endothelial cells, while ICAM-3 is highly expressed on leukocytes but is absent from endothelial cells. ICAM-4 and ICAM-5 are expressed exclusively on erythrocytes and on a subset of neurons in the telencephalon, respectively (192). Whereas CD11a binds ICAM-1-5, the complement integrins CD11b and CD11c both recognize ICAM-1, -2, and -4. CD11d appears to primarily bind ICAM-3 (207).

**ICAM-1** (CD54) plays a major role in the leukocyte adhesion cascade during extravasation into inflamed tissue. Circulating neutrophils start rolling on endothelial cell upon interaction with P-selectin and arrest under flow after activation by IL-8, which leads to CR3 activation by “inside-out” signaling. CR3 binds to the third Ig-like domain of ICAM-1, while LFA-1 binds the first Ig-like domain (185), which enables simultaneous binding of both integrin receptors. The binding of CR3 to ICAM-1 seems to be dependent on the level of ICAM-1 glycosylation, as CR3 binds with higher avidity to ICAM-1 with low glycosylation (186). The α_M_I domain is the major binding site for ICAM-1 (7, 72, 187, 188), but the conserved DXSXS motif of the β_2_-subunit may also be important (74). Recent studies indicate that ICAM-1 can bind to CR3 on the same cell (cis-ligation), with the integrin in a bent conformation but with an active headpiece. The cis-ligation between ICAM-1 and CR3 renders the bent conformation more stable (13) and was therefore proposed as negative regulation mechanism.

**ICAM-2** (CD102) has been shown to be a ligand of CR3 (190) through interaction with the α_M_I domain *via* its first Ig-like domain (191). A peptide derived from the Ig-like D1 domain competes with ICAM-2 binding to CR3 using a binding site distinct from iC3b, FX, and fibrinogen. Binding of the peptide activates CR3 and increases ICAM-1-mediated neutrophil adhesion as well as CR3´s binding to fibrinogen and iC3b (191).

**ICAM-4** is expressed on erythrocytes and binds isolated CD11a and CD11b in a divalent cation-dependent manner (192, 208), where CR3 is the stronger binding partner. ICAM-4 mediates binding *via* its Ig-like domains D1 and D2 (192). However, the binding site on CR3 has not been investigated in detail.

The junctional adhesion molecule (JAM) receptor family is also part of the type-I Ig superfamily and is found on platelets (193, 209), T-cells, and NK-cells as well as at the desmosomes of endothelial cells and intestinal epithelial cells (194). JAM receptors are believed to play a role in neutrophil-platelet interaction and neutrophil transepithelial migration. **JAM-C** binds to CR3 *via* a binding site that overlaps with that of fibrinogen (193).

Other counterreceptors involved in CR3-mediated leukocyte recruitment are **basigin** (CD147), **extracellular matrix metalloproteinase inducer** (EMMPRIN)**** (195), and the **receptor for advanced glycation end products (RAGE)**, the latter of which is a multiligand receptor expressed on inflamed vascular cells. In a mouse model, neutrophil extravasation into the peritoneum was primarily mediated by CR3 and ICAM-1, with RAGE accounting for 25% of total binding (196). In diabetic mice, RAGE-mediated extravasation increased to 50% (196), which highlights the relevance of this receptor interaction in diabetes. Similarly, **Thy-1 **(CD90), expressed on endothelial cells, may serve as a CR3-counterreceptor, as shown for psoriasis (197).

**SIRPα **(MFR), another member of the Ig superfamily, is involved in macrophage fusion, which accompanies chronic inflammatory conditions. The α_M_I domain mediates the interaction of CR3 with this counterreceptor (210) and exhibits binding to the soluble Ig1-2-3 ectodomains of SIRPα (199). Interestingly, the inactive form of α_M_I can still bind SIRPα, albeit to a lesser extent (199). Basic amino acids flanked by lipophilic residues were identified as binding motifs within SIRPα (49).

**CD40L** is a member of the tumor necrosis factor (TNF) superfamily and regulates B- and T-cell function by interacting with CD40. CD40L is expressed on macrophages, endothelial cells, and smooth muscle cells and plays a role in chronic inflammatory diseases, such as atherosclerosis. It stabilizes arterial thrombi by ligation to GpIIb/IIIa (211). However, CD40L also mediates atherogenesis independently of CD40 *via* CR3 (212). CD40L binds the α_M_I domain at a binding site that is distinct from fibrinogen. While the corresponding site on CD40L has not been investigated, CR3 binding does not compete with CD40 or GPIIb/IIIa binding (200). Blocking the CD40L:CR3 interaction by an anti-CD40L antibody or a cyclic peptide derived from the α_M_I binding site attenuated atherosclerosis in mice, which resulted in less inflamed, smaller, and more stable atherosclerotic lesions without affecting bleeding time and thrombus formation (200).

**Human leukocyte elastase (HLE)** and azurophilic granule proteins**** [such as **myloperoxidase** (201)] also bind to CR3. HLE binding can be blocked by a serine protease inhibitor, which indicates active site involvement in binding to CR3. **Azurodicin**, a homolog protein lacking protease activity, binds with even higher affinity, though the exact molecular meachanism and binding site on CR3 was not investigated (202). A complex of **pro-MMP-9** and CR3 is formed intracellularly in granules and translocated to the cell surface during cell activation (203). The binding sequence was mapped to the catalytic domain of pro-MMP-9, and binding of pro-MMP-2 and MMP-8 to the α_M_I domain has also been reported (204). Studies show that CR3 is cleaved by those proteases, which is a critical process for neutrophil detachment during chemotaxis (213).

**Pleiotrophin**, a cationic cytokine and growth factor, is expressed in injured tissue during regeneration and has affinity for heparin/glycosaminoglycans and CR3. Pleiotrophin is involved in the adherence and migration of neutrophils and leads to MAP kinase activation upon CR3 binding (205).

**Dynorphin A**, a member of the class of endogenous opioid receptor peptides, can induce phagocytosis and ROS production by ligation to CR3. Several CR3-binding motifs that involve basic and hydrophobic residues were identified by sequence analysis, with reported interaction with the α_M_I domain (206).

**DC-SIGN** is a C-type lectin expressed on dendritic cells, where it serves as an adhesion receptor for endothelial cells and PMNs. DC-SIGN binds to the sialyl-Lewis^X^ motif of CR3, which is expressed solely on PMNs (32).

#### Leukocyte Migration on Extracellular Matrix

The **extracellular matrix **(ECM) is composed of various proteins, such as collagen, elastin, fibronectin, laminin, thrombospondin, and glycosaminoglycans (214), and is involved in neutrophil recruitment to inflammation sites (215). Several ECM components have been identified as CR3 ligands (**Table 6** and **Supplementary Table 6**), and binding of **thrombospondin**, **vitronectin** (217), **fibrinogen**, and **fibronectin** activates PMN and induces H_2_O_2_ secretion (216). CR3 interactions have also been confirmed for other ECM components, such as collagen IV**** (215), **undulin**, **laminin** (240), and **lumican** (243). **Mindin** appears to play a dual role in mediating leukocyte migration and serving as an opsonin (244, 261). It also induces phagocytosis *via* the Syk pathway and activates NFκB (245). Similarly, **CCN1** and **CCN2** binding to CR3 leads to NFκB activation (246–248). The oxidation of polyunsaturated fatty acids forms **2-(ω-carboxyethyl)pyrrole** (CEP), which can then modify ECM proteins. CEP is associated with inflammation and mediates the expression of pro-inflammatory cytokines in macrophages. It also increases adhesion and migration *via* binding to CR3 and CR4 (249).

**Table 6:** Leukocyte migration on extracellular matrix.

Ligand	Site on CR3	Site on ligand	Function	
Thrombospondin	–	–	ROS secretion	(216)
Vitronectin	α_M_I, overlapping with fibrinogen, divalent cation dependent	Not RDG, not somatomedin B domain	–	(216, 217)
Fibrinogen	α_M_I, not MIDAS, not cation dependent, overlapping with iC3b, but not directly competing, beta I-like domain is involved in binding	γ-chain, β-C domain	PMN migration of fibrinogen, proinflammatory, involvement in sickle cell anemia, muscle dystrophy	(46, 47, 80, 188, 189, 218–238)
Fibrinogen-420	competes with NIF	αEC	–	(225)
Fibronectin	α_M_I	–	Reduces migration	(215, 216, 239)
Collagen	–	GFOGER of collagen	ROS secretion, PMN migration in inflamed tissue	(215, 240, 241)
Undulin		–		(240)
Laminin		–	ROS secretion	(215, 240, 242)
Lumican	–	–	–	(243)
Mindin	α_M_I	FS domain	Opsonization, phagocytosis	(244, 245)
CCN1 and CCN2	α_M_I	C-terminus of CCN1	Expression and secretion of proinflammatory mediators	(246–248)
CEP	α_M_I	–	Macrophage migration	(249)
Plasminogen	α_M_I, competition with P2 (fibrinogen derived), tranexamic acid	Kringle domains 1, 2, 4, and 5	–	(250)
Angiostatin	α_M_I, further binding sites possible.	Kringle domain 4	Inhibition of neutrophil extravasation, reduction of NFκB activation and TF expression	(251)
Lipoprotein(a)	α_M_I	Apo(a) domain, binding is upregulated by preincubation with homocysteine	Increased NFκB activation and TF expression, increased transmigration/cell recruitment	(252)
uPAR	β-propeller	–	Priming, leukocyte recruitment and migration, cis interaction; enhances fibrinogen binding and plasminogen activation	(253–256)
tPA (tissue plasminogen activator)	Competes with NIF	CR3, fibrin and tPA form adhesive complex	Enhanced fibrin binding, aggregation, and interaction with Annexin A2	(61, 257)
Annexin A2	–	–	–	(61)
NB1 (CD177)	–	–	ROS production, neutrophil activation	(258)
LRP1 (CD91)	α_M_I (opposite MIDAS) competition with fibrinogen	–	Detachment of macrophage through internalization	(257, 259, 260)

**Fibrinogen** is the zymogen form of fibrin, which is primarily known as a central component of hemostasis. However, fibrinogen also ligates CR3 and CR4 and plays important roles in inflammatory processes. Early observations showed that PMNs bind to fibrin and can migrate on a fibrinogen matrix (262, 263), with CR3 identified as the responsible receptor (218). The γ-chain of fibrinogen, which also interacts with the platelet integrin GpIIb/IIIa *via* its RGD motif, can bind CR3 (218) independently of RGD (219–222). A cyclic peptide, derived from the fibrinogen γ-chain (CNRLTIGC) (222), also binds to CR3 (250). Interestingly, the binding motifs within the fibrinogen structure are buried in soluble fibrinogen, but become accessible to CR3 upon fibrinogen binding to surfaces or proteolytic cleavage (46, 223).

**Fibrinogen** binding to CR3 is likely the best-characterized ligand interaction of this versatile integrin receptor (188, 218, 219). A study that associated sequence homologies in the I domains of β_2_ integrins with fibrinogen binding identified the βD-α5 loop (K245-R261) as the binding site within the α_M_I domain (47, 221, 224). This loop is not part of the MIDAS, so fibrinogen binding occurs cation-independently. Competition experiments with FX and iC3b identified partial overlap in the binding regions (224). The detrimental influence of mutations within the β_2_ I-like domain on fibrinogen binding (47) may be caused by regulatory roles rather than direct binding (264), since a α_M_I-less CR3 mutant does not bind fibrinogen (76). Alternative splicing produces two forms of fibrinogen called Fg-340 and Fg-420, with greater abundance found for Fg-340. Both forms function similarly in regard to clotting, crosslinking of FXIII, and fibrinolysis. Interestingly, Fg-420 features an extended domain, which has 40% sequence homology to the γ-chain. This results in a threefold higher affinity of CR3 for Fg-420 relative to Fg-340 (225).

The CR3 interaction with fibrinogen is detrimental for inflammatory responses and is distinct from the role of fibrinogen in hemostasis. Deletion of the CR3-binding motif diminishes adhesion of primary neutrophils and macrophages (226), thereby reducing renal pathology in mice with sickle cell anemia due to the decreased production of IL-6, IL-1, and TNFα (227). The CR3-fibrinogen interaction is also responsible for phagocyte accumulation at sites of biomaterial implantation (265).

**Plasminogen** is the zymogen form of plasmin, which is responsible for dissolving fibrin clots. It interacts with the ECM after plasma leakage into tissue and can bind to leukocytes. Besides VLA-4, CR3 was identified as the responsible receptor, with binding mediated by the α_M_I domain (250). **Angiostatin**, which consists of the kringle domains 1-3 or 1-4 of plasminogen, is expressed during inflammation and wound healing. It competes with fibrinogen and ICAM-1 binding to CR3, which inhibits the extravasation of neutrophils and reduces inflammatory responses (251). **Lipoprotein(a)**, a risk factor for coronary heart disease, has pro-inflammatory effects and is highly homologous to the kringle domain 4 of plasminogen, which enables binding to CR3. Preincubation with homocysteine, another risk factor for coronary heart disease, leads to enhanced binding of lipoprotein(a) to CR3 and, consequently, increased cardiovascular risk (252).

The **urokinase receptor** (uPAR, CD87)**** is a GPI-tethered glycoprotein binding urokinase (urokinase-type plasminogen activator, uPA), a serine protease that activates plasminogen to plasmin. uPAR directs plasminogen activator activity to specific areas on the cell surface and plays a central role in thrombolysis, ECM degradation, and leukocyte migration. The association between CR3 and uPAR (59, 253), and its impact on leukocyte recruitment, was reported in the 1990’s (58, 254). The interaction is enhanced by the addition of Zn^2+^, an effect that is mediated by uPAR (266). The uPAR binding site has been mapped to the W4 blade of the β-propeller of the CD11b subunit (255). However, CR3 is postulated to build complexes with GPI-anchored proteins *via* the lectin domain (60), thereby acting as transmembrane signaling adaptor. Ligation of uPAR and CR3 leads to CR3 activation (priming), similar to ligation of FcyRIIIB and CR3 (58, 59). The complex of urokinase, uPAR, and CR3 enhances CR3-binding to fibrinogen, with no effect on ICAM-1 binding. The enhancement is initiated by the urokinase:uPAR:CR3 complex and is mediated by FAK phosphorylation *via* the MAP kinase pathway (256). Furthermore, uPA is attracted to this complex, which both enables the cleavage of ECM components to facilitate neutrophil migration and negatively modulates the stability of the uPAR:CR3 complex (59). A phage–display-derived peptide against uPAR (M25, STYHHLSLGYMYTLN) can disrupt the uPAR:CR3 complex, which attenuates binding to fibrinogen (255).

Like uPA, **tissue plasminogen activator** (tPA) is a serine protease on endothelial cells that catalyzes the conversion of plasminogen to plasmin. In analogy to urokinase, tPA binds to CR3 and enhances recognition of fibrin, which can be blocked by NIF (257). tPA also forms a complex with **Annexin A2**, which is a Ca^2+^ and phospholipid-binding protein found intra- and extracellularly. It has no transmembrane domain and associates transiently with the membrane, without the ability to transmit signals into the cell. Annexin A2 was identified as receptor for tPA and co-immunoprecipitates with CD11b. This complex of Annexin A2, tPA, and CD11b initiates an “outside-in” signal that activates integrin-linked kinase (ILK) and, subsequently, the NFκB pathway. This signaling is dependent on CD11b, as shown in control experiments with a CD11b-neutralizing antibody and CD11b knock out mice (61). **Neutrophil antigen BI **(NB1/CD177), a GPI-anchored protein in the Ly6/urokinase plasminogen activator receptor family, is also reported to bind CR3, which leads to neutrophil activation and release of ROS (258, 267).

LDL receptor-related protein (LRP) is a large endocytic receptor on macrophages, which is involved in macrophage migration *via* interaction with β_2_ integrins (259). **LRP-1** (CD91) binds to a complex of CR3, tPA, fibrin, and PAI-1, which is subsequently internalized. In turn, this leads to detachment of the macrophages and their migration on the ECM (257). Essential amino acids for this complex formation reside on a site opposite the MIDAS, though binding is not limited to the α_M_I domain (260). A soluble form of LRP1 (sLRP1), which is found in elevated concentrations (~ 10 nM) in plasma during inflammation, competes with CR3 binding to fibrinogen, which might confer a regulatory mechanism for resolving inflammation (260).

**Fibronectin (Fn)**, a known ligand of β_1_ integrins such as α_5_β_1_, also binds to CR3 *via* the α_M_I domain. Interestingly, binding of Fn to CR3 does not increase migration, like binding to α_5_β_1_, and in fact, the opposite effect is observed. Considering the correlation with the density of CR3 surface expression, this effect likely occurs in response to inflammatory signaling, when CR3 is shuttled from intracellular storage vesicles to the cell surface, thereby increasing the receptor density by approximately a factor of 10 (239).

#### Leukocyte Interaction With Hemostasis and Thrombi

Any breach of the body’s physical barriers requires rapid, coordinated defense system responses to stop blood loss, initiate wound repair, and combat microbial intruders. CR3 interactions with ligands of the contact and hemostatic system (**Table 7** and **Supplementary Table 7**) can contribute successful defense reactions, but may also play a role in thrombo-inflammatory disorders. Firm adhesion and trans-platelet migration of leukocytes on vascular thrombus is dependent on CR3 when **GPIbα** is a counter-ligand (268). GPIbα and CR3 binding induces a bidirectional signal that yields proinflammatory and prothrombotic responses. By blocking CR3:GPIbα-mediated leukocyte–platelet interactions with antibodies, leukocyte accumulation after arterial injury could be reduced (286). In addition, responses to tissue injury in models of vasculitis (287), glomerulonephritis (288), and EAE (289) have proven to be dependent on CR3:GPIbα binding. By blocking this interaction, NET formation could be prevented (290). The importance of this interaction was also demonstrated using CR3-deficient mice, which were protected in a thrombotic glomerulonephritis model (288) and exhibited impaired thrombus formation without affecting the coagulation time, platelet count, and activation (269). Furthermore, CR3 clustering from GPIbα binding induced phosphorylation of PKCδ and downregulated Foxp1, thereby reducing tissue factor (TF) expression (269). The CR3:GPIbα binding is promoted by the α_M_I domain (228), with a binding site that is distinct from that of fibrinogen, ICAM-1, and JAM-C (286). The leucine-rich C-terminal flanking region of GPIbα (GPIbαN) was identified as the interaction site (228), which binds with a glutamic acid to the α_M_I MIDAS, similarly to the internal ligand of the α7-helix (229). Further interactions are formed towards a groove on the surface of the α_M_I domain created by F246 and R206 (229).

**Table 7:** Leukocyte interaction with homeostasis and thrombi.

Ligand	Site on CR3	Site on ligand	Function	
GPIbα	α_M_I, competing with heparin, fibrinogen, glucosamine	Leucine- rich N-terminal region	Adhesion and trans-platelet migration, pro-inflammatory and pro-thrombotic, NETosis	(228, 229, 268–270)
Fucoidan	Divalent cation dependent	Sulfates essential for binding	Elastase release, reduced mobilization of bone marrow nucleated cells	(57, 271)
CD44v3	Divalent cation dependent	Heparan sulfate, Heparinase treatment almost completely inhibited the binding	PMN-Epithelial Adhesion	(272)
Heparin heparan	α_M_I, competes with fibrinogen, FX, ICAM-1, iC3b	sulfates essential for binding	–	(168, 268, 273, 274)
Glucosamine	α_M_I	–	Anti-thrombotic, inhibits ligation to GPIbα	(269)
FX (not FXa)	Not mainly mediated by α_M_I, but iC3b competing and Ca^2+^ dependent	Three distinct sequences surrounding the catalytic site:	Gets activated to FXa by degranulation of activated leukocytes (cleavage by cathepsin G)	(76, 188, 275–277)
Kininogen	α_M_I, overlapping with ICAM-1 and fibrinogen, divalent cation dependent, competition with ICAM-1	Domain 3, mainly the C-terminus of domain 5	Elastase release, formation of GPIbα:CR3, release of cytokines (TNF-α, IL-1β, IL-6) and chemokines (IL-8 and MCP-1), in complex with uPAR, LFA-1 and gC1qR?	(230, 270, 278–282)
Thrombomodulin	–	Thrombomodulin domain 3 is required for binding to CR3.	Interferes with ANCA binding, inhibition of neutrophil extracellular trap formation	(283, 284)
protein-C receptor	–	–	–	(285)

**Heparin** (HMW and LMW), an anticoagulant in pharmacotherapy, adheres to neutrophils in an interaction mediated by β_2_ integrins (273). The structural similarity of heparin to β-glucans suggests binding *via* the lectin site. However, the α_M_I domain appears to mediate heparin binding (273), and heparin competes with CR3-binding of FX, ICAM-1, and iC3b, even at lower concentrations than needed for anticoagulation (weak prolongation of the activated partial thromboplastin time (aPPT), 0.1–1 U/mL) (274). Interestingly, desulfated heparin loses its affinity for CR3, which indicates that sulfate groups are key to the interaction (273). A similar influence of sulfation was reported for fucosylated proteoglycans (57), such as **fucoidan** (271). Accordingly, glucosamine, heparin, and related mucopolysaccharides of the sulfated glucosamine glycan type (except for chondroitin sulfate) bind to CR3 and can mediate the binding of proteins decorated with glycans to CR3, as seen with **CD44v3** (272).

**Glucosamine** has been described as an inhibitor of the CR3:GPIbα interaction through direct binding to the α_M_I domain (269). Glucosamine decreased thrombus formation in an *in vivo* model of carotid artery photochemical injury without prolonging bleeding time, which demonstrated its anti-thrombotic potential (269).

Activated monocytes induce coagulation processes through CR3-mediated binding of **FX** in a calcium-dependent and saturable manner (275, 291). Although the binding site has been postulated to lie outside the α_M_I domain (76, 188), FX binding can be blocked by iC3b (219). An FX-derived peptide also directly competes with ICAM-1 (35). Interestingly, while FX is a ligand for CR3, activated FXa is not able to bind (276).

**High-molecular-weight kininogen** (HMWK) is an abundant plasma protein that acts as an initiator of the contact activation pathway during coagulation. It circulates as a complex with plasma prekallikrein (PPK) and is activated upon binding to negatively charged surfaces, where it facilitates the activation of FXII and prevents thrombin binding. When cleaved to HMWKa by plasma kallikrein (PK), it liberates the potent vasodilator, bradykinin. HMWK can bind to neutrophils with high affinity (K_D_ 9–18 nM) and is dependent on Zn^2+^ (278). Of note, HMWKa can still bind to CR3 (K_D_ ~ 60 nM) (279). HMWK domain 3 and the C-terminus of domain 5 were identified as binding sites (270, 280, 281), which seems to overlap with fibrinogen (230) and ICAM-1 (279, 280), but not FX, and indicates involvement of the α_M_I domain. HMWK-binding to CR3 enhances the formation of CR3:GPIbα complexes by 2-fold, likely by bridging the two receptors *via* domain 5 (to CR3) and domain 3 (to GPIbα) (270). The CR3-HMWK interaction leads to an increased release of neutrophil elastase (NE) (278). Meanwhile, HMWKa binding to mononuclear cells leads to the release of cytokines (TNF-α, IL-1β, IL-6) and chemokines (IL-8 and MCP-1) (282).

Besides promoting coagulation, CR3 also interacts with anticoagulant factors. **Thrombomodulin** is a multi-domain anticoagulant with anti-inflammatory properties, where domain 3 is a ligand for CR3, while domains 1 and 2 are important for its anticoagulant and anti-inflammatory effects (283). Furthermore, thrombomodulin interferes with the binding of anti-neutrophil cytoplasmic autoantibodies, which inhibits NET formation (284). The **endothelial protein C-receptor** (EPCR) plays a crucial role in the protein C anticoagulant pathway by promoting protein C activation. Its soluble form binds CR3 on activated neutrophils. Further investigation is needed to identify the molecular binding site on EPCR or CR3 (285) and to elucidate the functional implications of ligation.

### Involvement of CR3 in Additional Interactions

There are close to 100 reported ligands of CR3, but not all can be associated with a functional cluster, despite their importance in physiological/pathophysiological processes (**Table 8** and **Supplementary Table 8**). Intracellular proteins located in the endoplasmic reticulum (ER) interact with CR3, which supports the translation of CR3 and its trafficking to the cell surface. **Protein disulfide isomerase** (PDI) contributes to the proper folding of proteins by catalyzing disulfide bond formation in the ER, but is also expressed on cell surfaces. In the case of CR3, disulfide bonds stabilize the open activated conformation (296), and PDI is involved in neutrophil adhesion during vascular inflammation (292). **B-cell receptor-associated protein 31** (BAP31) is an ER-associated transmembrane protein that is involved in regulating cellular anterograde transport. Its reported binding to CR3 might play a role in protein trafficking to the cell membrane (293).

**Table 8:** Involvement of CR3 in additional interactions.

Ligand	Site on CR3	Site on ligand	Function	
Protein disulfide isomerase (extracellular)		–	Neutrophil recruitment, regulates fibrinogen binding and integrin clustering	(292)
BAP31	Binding was independent of α_M_I-domain	–	–	(293)
CD22	Binds CD11b glycosylation, reduced upon neuramidase treatment		Not binding R77H (SLE)	(66)
CD23	Inhibited by FX, divalent cation dependent	–	ROS production, release of proinflammatory cytokines	(294)
IL-13Rα1	β-propeller, CD11b leg	D1, D3, D2	Cis ligation as negative feedback reduced foam cell formation	(63)
Steel	–	–	Binding of monocytes to steel stents can lead to restenosis	(295)

**CD22** is a glycoprotein on B-cells involved in the negative-feedback regulation of B-cell receptor (BCR) signaling through formation of a complex with CD11b, BCR Lyn, and SHP-1. The negative feedback is dependent on functional CD11b, as R77H polymorphism results in decreased co-localization with the CD22:Lyn : SHP-1 complex. In turn, this leads to enhanced B-cell proliferation and Ca^2+^ influx (66). Interestingly, the R77H mutation seems to affect glycosylation, since the negative feedback interaction of CD22 with CD11b was also diminished when CD11b was treated with neuramidase, and lectin binding was abolished when R77H CD11b was transfected into CHO cells.

**CD23**, a C-type lectin, is a ligand for CD11b and CD11c on resting monocytes. When bound, it induces NO formation and the secretion of pro-inflammatory cytokines (IL-1β, IL-6, and TNFα) (294).

The cytokine interleukin 13 (IL-13) partially shares the signalling pathway with IL-4 through engaging with the IL-4 receptor type II. This receptor is a heterodimer, consisting of **IL-13Rα1** and IL-4Rα, which assembles after ligand binding (IL-13 binds IL-13Rα1; IL-4 binds the first IL-4Rα chain). Ligand binding to the receptor triggers the expression of pro-inflammatory genes such as 15-LO and CD36. CR3 colocalizes with and binds the IL-13Rα1 chain *via* cis-ligation as a negative-feedback mechanism, thereby reducing the formation of foam cells (63, 64). Potential interaction areas were identified within the W5 blade of the β-propeller, the α_M_I domain, and the CD11b leg from a co-evolution analysis of CR3 and IL-13Rα1 (63).

Interestingly, adhesion of monocytes to steel used for stents was shown to depend on CR3, which can lead to restenosis. The binding could be blocked by coating with the semiconductor silicon carbide (295).

### CR3 Ligation in Diagnosis and Therapy

CR3 has long been an interesting translational target due to its highly dynamic involvement in clinically relevant processes that include inflammation and thrombosis and immune cell adhesion, activation, and trafficking. As a result, a plethora of antibodies, inhibitors, and synthetic molecules have been developed that interact with CR3 (**Table 9** and **Supplementary Table 9**).

**Table 9:** CR3 as a potential therapeutic target.

Ligand	Site on CR3	Site on ligand	Function	
Imprime PGG	–	–	Activate anti-cancer innate immune effector functions	(153–156)
Gu-4	Lectin binding site	Oligosaccharides	Inhibition of leukocyte adhesion and transendothelial migration	(297)
Hydroxyethyl starch	–	–	Reducing migration, and chemotaxis of activated PMN	(298)
**Antagonists**				
Abciximab	–	–	Blocking of different CR3 functions	(299)
Leumidin		–	Inhibition of neutrophil adhesion	(300, 301)
Covalent small molecule	Inhibiting CR3:iC3b	–	Anti-inflammatory by reduced neutrophil emigration	(302)
Gupta group	Inhibited binding to fibrinogen, IC_50_ < 1 µM	–	–	(303)
XVA143	Antagonists to the I-like domain, inhibiting binding of iC3b and ICAM-1, IC_50_ 0.9 µM	–	–	(304)
Simvastatin	α_M_I (MIDAS) and other residues	Carboxylic acid	Inhibition of monocyte binding to iC3b	(48)
E/DDGW	Competes with MMP-9	–	–	(204)
CP[CFLLGC]C	Divalent cation dependent, competes with ICAM-1, vWF and collagen	–	Inhibits leukocyte adhesion to ICAM-1	(305)
GYRDGYAGPILYN	Competes with ICAM-1	–	–	(306)
**Agonists**				
2-thioxothiazolidin-4-one	Competing with DDGW peptide, enhanced binding of fibrinogen and proMMP-9	–	–	(307)
Leukoadherins (LA-1/ADH-503)	EC_50_ 10- 40 µM, increasing binding to fibrinogen	–	–	(34, 308–314)

CD11b expression has been a longstanding immunological marker to identify subsets of leukocytes. However, the dynamically increased expression in various disease states also points to the utility of CR3 as a biomarker. The value of monitoring CR3 expression has been shown in acute myeloid leukemia (315, 316), childhood acute lymphoblastic leukemia (317), neonatal sepsis (318), inflammatory lung disease (319), metabolic syndrome (320), Alzheimer’s disease (321), and gastric cancer. In cancer, elevated numbers of CD11b^+^ cells are often considered predictors of a poor prognosis (322). Recently, CR3 expression was found to be elevated in hypoxic COVID-19 patients, but not in patients with mild symptoms. Therefore, CR3 may serve as marker for disease severity in COVID-19 patients (323).

The association of CR3 and other β_2_ integrins with immuno-inflammatory dysregulation in numerous diseases has sparked considerable therapeutic interest in this receptor class (2–4, 26). For example, CR3 has been suggested as an intervention target for ischemic stroke in a rodent model due to its upregulated expression and the beneficial outcome (e.g., reduced neutrophil infiltration) of receptor inhibition. However, clinical trials with a NIF-based CD11b inhibitor and the anti-CD18 antibody rovelizumab for treatment of reperfusion injury in stroke were halted due to lack of efficacy (324). There has been mounting interest in recent years in CR3 for its role in CNS development, homeostasis, and neurodegenerative diseases. However, the biology of CR3 in neurological processes is complex. In rodent models, neuroinflammation in Alzheimer’s and Parkinson’s disease is reduced in a CR3-dependent manner by phagocytotic plaque clearance (172) and Aβ level reductions (174). Conversely, binding of β-amyloid (325) and α-synuclein (178) to CR3 induces production of ROS.

The clinical introduction of β_2_ integrin modulators has been met with challenges after efalizumab, an anti-CD11a mAb for the treatment of psoriasis, was retracted from the market for reactivation of John Cunningham virus. The virus induced potentially life-threatening progressive multifocal leukoencephalopathy (PML) (326). In contrast, the CR3 small molecule agonist **LA-1/ADH-503** had positive effects in various inflammatory- and leukocyte-driven disease models in rodents (308, 327, 328) in addition to Aβ42 levels (174). Recent investigations identified a role of CD11b activation in anti-tumor innate immunity (34) and possible therapeutic applications in pancreatic (329) and lung cancer (330). LA-1/ADH-503 is currently under clinical investigation (licensed to Gossamer Bio as GB1275) in Phase I/II trials for solid tumors (NCT04060342). **Imprime PGG**, a β-glucan (see section 3.1.2.1****) that targets the lectin domain of CR3 primes phagocytes to exert cytotoxicity against tumor cells (152). Combination therapies of Imprime PGG with immune checkpoint inhibitors are currently under evaluation in Phase 2 trials for treating melanoma and metastatic breast cancer (NCT02981303). The efficacy and safety of CR3 agonists remains to be determined.

**Figure 5:**
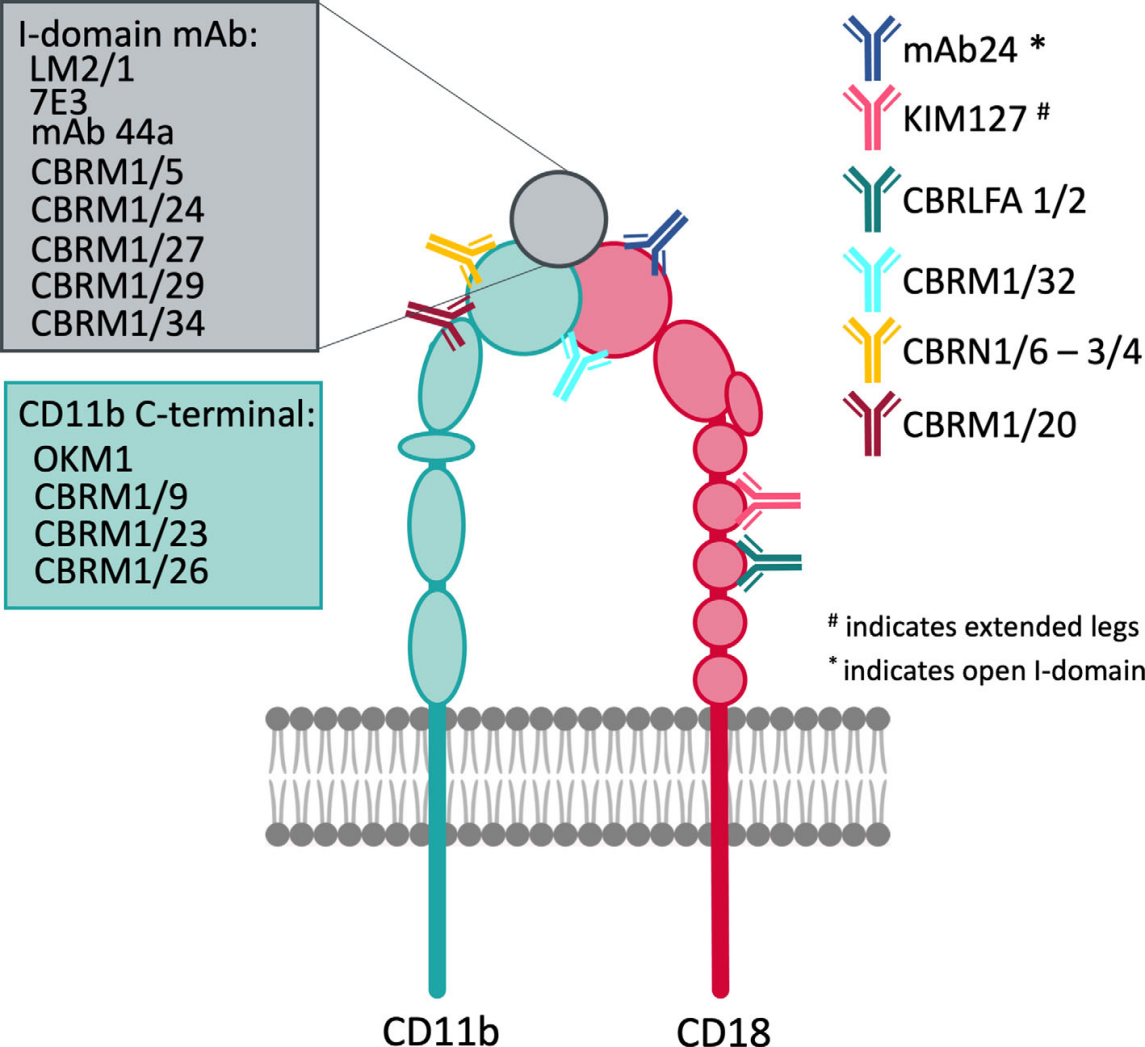
Overview of important antibodies developed against CD11b and CD18 mapped to their reactive regions.

Despite the challenges in clinical applications, various CR3-targeting compounds have been developed that include mAbs, recombinant proteins, peptides, and small molecules. While they are not all designed as therapeutics, they are valued for their potential towards unraveling the complex biology of CR3. Indeed, several mAbs have been developed as probes to investigate binding sites, conformational states, and functional outcomes of CR3 ligation in *in vitro* settings (**Figure 5**) (331). **Abciximab** (7E3 fab), a therapeutic antibody against GpIIb/IIIa and platelet aggregation inhibitor, binds to CR3 and competes with ICAM-1, fibrinogen, and FX (332). However, this antibody is no longer in clinical use due to supply problems.

Saccharides that impact leukocytes might induce effects similar to Imprime PGG when bound to the lectin site of CR3. The acidic fraction of lactose-derived oligosaccharides from human breast milk inhibits leukocyte adhesion (333) and transendothelial migration (297). This has led to the preclinical evaluation of lactosyl derivatives** (Gu-4)** for the treatment of severe burn-related shock and sepsis in animal models (297, 334). Moreover, **hydroxyethyl starch** 130/0.4 (HES), a synthetic colloid used in volume replacement therapies, binds to fMLP-activated neutrophils *via* CR3, which leads to “outside-in” signaling *via* increased activation of the PI3K/Akt pathway, with reduced migration, chemotaxis, and impaired binding to fibrinogen (298).

**Figure 6:**
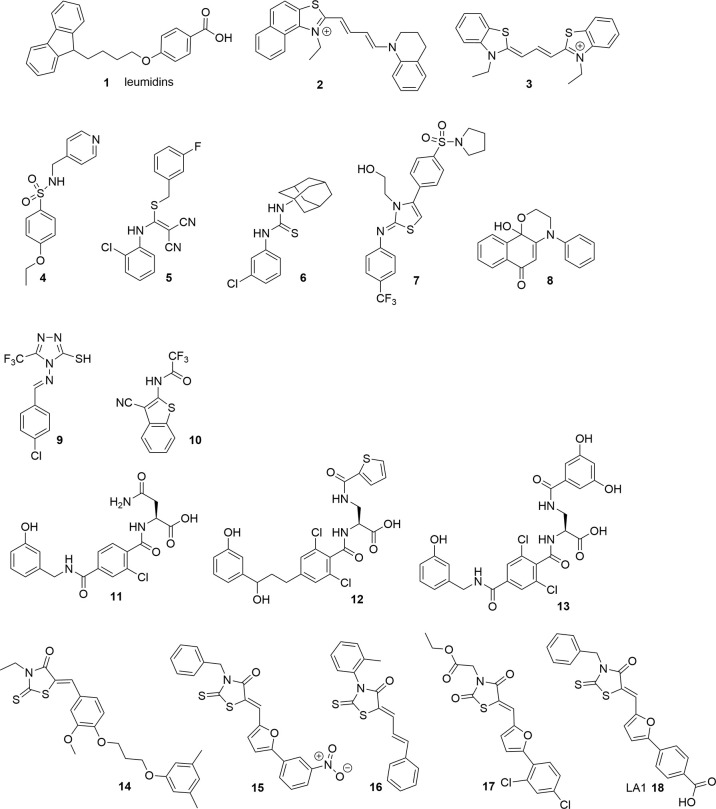
Small molecules developed as possible CR3 modulators.

Attempts have been made to identify selective small-molecule CR3 antagonists. Fluorenylalkanoic and benzoic acids, which originated from Fmoc amino acid derivatives (**leumidins**) that inhibit neutrophil recruitment, were among the first compounds proposed as novel leukocyte adhesion inhibitors (300). Next-generation compounds replaced the carbamate moiety and increased activity 10-fold with the lead molecule (**Figure 6** and **Compound 1**) that had an IC_50_ = 5 µM in neutrophil adherence assays (300). While CR3 has been suggested as the responsible target, this has not been confirmed by direct binding studies (301).

In a screening assay with immobilized CR3 and iC3b as binding partners, two compounds (**Figure 6** and **Compounds 2; 3**) were identified as inhibitors of the CR3:iC3b interaction, with IC_50_ values of 0.14 and 0.33 µM, respectively. At concentrations up to 10 µM, they showed no binding to LFA-1, α_V_β_3_, α_4_β_1,_ or α_4_β_7_. Interestingly, the binding of both compounds is not easily reversed, and covalent binding can be induced by light (302).

The Gupta group screened >92,500 commercially available compounds in a high-throughput screening assay to identify antagonists competing with fibrinogen binding to CR3, the addition of Mn^2+^ rendered the integrin active. From this work, 63 compounds were identified that inhibit fibrinogen binding with an activity < 1 µM, though only a subset of compounds was disclosed (**Figure 6** and **Compounds 4**–**10)** (303).

Roche and Genentech patented compounds characterized as LFA-1 antagonists (**Figure 6** and **Compounds 11–13**), which perturb the interface between the αI domain and βI-like domain. Consequently, these compounds lock the I-like domain in an active conformation, which leads to integrin extension, while the I domain is locked in its inactive state (304). In addition to being antagonists of LFA-1, the compounds also inhibit CR3 binding to iC3b (11, 3µM and 12, 0.1 µM, 13 XVA143) (304).

**Simvastatin** and other statins were identified as allosteric inhibitors of LFA-1, which bind to the so-called L-site on the opposite side of the MIDAS (335). In the CR3 interaction with simvastatin, a recent crystal structure shows that the carboxylic acid of the hydrolyzed lactone interacts with the MIDAS to form a salt bridge, which seems to be the only relevant contact formed. Further studies are needed to explore the specificity and impact of this interaction. In surface plasmon resonance experiments, simvastatin partially inhibited the interaction of CR3 α_M_I with iC3b and C3d, whereas the CR3:ICAM-1 interaction was not inhibited. At micromolar concentrations, simvastatin inhibits monocyte binding to iC3b in flow cytometry and adhesion assays. However, this interaction does not explain the plethora of pleiotropic effects of simvastatin (48).

Several phage display screening campaigns aimed at β_2_ integrins have been conducted, which panned against full CR3 (305), the α_M_I domain (204), or one of its ligands (231). Koivunen et al. panned against the full CR3 purified from blood samples with CX_7_C and CX_9_C libraries (cyclic peptides with two fixed cysteines and 7 or 9 variable positions, respectively) in Mn^2+^-containing buffer. They identified peptides that bind the α_M_I domain in a divalent cation-dependent manner, with cross reactivity to LFA-1 and CR4. The minimum consensus sequence was CLLGC, where the most potent peptide (CP[CFLLGC]C) had an IC_50_ of 20 µM. This peptide inhibited leukocyte adhesion to ICAM-1 and von Willebrand factor (305). A different phage display screen against the purified α_M_I domain with a pool of random peptide libraries (CX_7-10_C and X_9-10_) yielded E/DDGW as a minimal consensus sequence. This sequence is also found in MMPs, which were identified as CR3 ligands (see above). The DDGW peptide is an effective inhibitor of MMP-9-binding to the α_M_I (IC_50_ = 20 µM) as well as the α_L_I domain, though not in competition with CR3-binding to ICAM-1 (204). As an alternative target, the anti-α_M_I mAb 44a, known to block binding of fibrinogen, iC3b, and C1q, but not ICAM-1, was also used for phage display panning. Screening of a CX_6_C library displayed on M13 filamentous phage yielded the peptide (CRLKEKHC), which can dose-dependently inhibit binding of fibrinogen to CR3 (IC_50_ = 3.35 µM) (231).

Another approach to developing binding peptides is to derive them from the complementarity-determining regions of anti-CR3 antibodies, which block binding to ICAM-1. The most potent peptides derived from this method inhibited binding of CR3-expressing CHO cells to ICAM-1 with an IC_50_ = 30 µM. Generation of a focused library based on the identified sequence yielded the active peptide (GYRDGYAGPILYN) (306, 309).

A high-throughput screen was implemented to identify antagonists in competition with DDGW-displaying phages from a pool of 10,000 commercially available small molecules. This screen generated 19 lead compounds with significant and reproducible inhibition of DDGW-phage binding. Most of the compounds had a common 2-thioxothiazolidin-4-one substructure (**Figure 6** and **Compound 14–16**). Surprisingly, although the compounds inhibited phage binding to the α_M_I domain, they also greatly enhanced the binding of pro-MMP-9 and fibrinogen. A binding site in a hydrophobic cavity that appears in the open conformation of the α_M_I domain was proposed using computational modeling methods. **Compound 16** was tested *in vivo* and reduced inflammation response as determined by reduced neutrophil emigration (307). Similarly, a high-throughput screen was conducted by the Gupta group with a library of >13,500 small molecules to identify compounds that increased CR3 binding to fibrinogen in Ca^2+^/Mg^2+^-containing buffer. A large subset of the hits contained a central 2-thioxothiazolidin-4-one motif, which was a previously reported hit structure in an independent screen against the α_M_I-domain in competition with DDGW-phages (307). **Compound 17** (**Figure 6**) had 2-fold higher binding to CD11b over CD11a, with an EC_50_ = 13.6 ± 5 µM. *In silico* docking experiments also suggested that the compounds (termed leukadherins****) bind between helix α7 and α1 and the central β-sheet (310), in agreement with prior reports (307). In contrast to activating antibodies, leukadherins do not induce integrin clusterin or intracellular signaling due to a lack of a global conformational change during binding (311). **Leukadherin-1** (LA-1, ADH-503)**** (**Figure 6** and **Compound 18**), the most prominent compound of the class, is currently in clinical development as GB1275 (see above). It increases CR3-dependent cell adhesion while reducing chemotaxis and transendothelial migration. Consequently, it has showed efficacy in leukocyte-dependent disease models, such as acute peritonitis in mice, vascular injury in rats, and experimental nephristis in mice (312). Furthermore, LA-1 can suppress human innate inflammatory signaling (313), TLR-dependent inflammation, and autoimmunity in SLE (308), in addition to ameliorating endothelial barrier damage in critically ill patients (314). In addition, LA-1 promotes pro-inflammatory macrophage polarisation, which drives anti-tumor innate immunity (34).

## Conclusion and Outlook

Integrins are a versatile class of cell surface receptors with a broad spectrum of functions in inter-cellular communication, tissue development, maintenance, and repair. Integrins have a well established link to the ECM and play a role in leukocyte migration and function. Within this family, CR3 has particularly broad ligand promiscuity. Its ability to bind ligands spans the I-domain, adjunct areas, and distinct regions like the lectin domain. Whereas CR3 ligands were first reported 40 years ago, recent crystallographic insights and binding studies reveal a considerably complex picture. While CR3 has close to 100 ligands, binding for most of these have been demonstrated through biological methods without full investigation at the molecular level, including specific binding sites, competition with other ligands, or functional implications. Additionally, the treasure chest of CR3-mediated interactions and functions is still not exhausted. For example, recent studies showed a link between the complement system and integrins (especially LFA-1) in orchestrating metabolism and immunity (1) through regulation of the intracellular complement system (i.e. the complosome) (336), which is required for Th1 responses. Cultured cells can take up some of the required C3 for those responses in the form of hydrolyzed C3, which serves as ligand for CR3. Looking forward, further and in-depth investigations are needed to elucidate the complex roles of leukocyte integrins and their crosstalk across numerous pathways.

## Author Contributions

All authors contributed to the conception, writing, and editing of the manuscript. All authors contributed to the article and approved the submitted version.

## Funding

This work was supported by grants from the Swiss National Science Foundation (31003A_176104 to DR) and the University of Basel (to CL).

## Conflict of Interest

The authors declare that the research was conducted in the absence of any commercial or financial relationships that could be construed as a potential conflict of interest.
