# Supplementary material for: The Promiscuous Profile of Complement Receptor 3 in Ligand Binding, Immune Modulation, and Pathophysiology
Source: Front Immunol. 2021 Apr 29;12:662164. doi: 10.3389/fimmu.2021.662164 (PMC8118671; doi:10.3389/fimmu.2021.662164)
Supplement: Supplementary file 1 [file Table_1.docx]

Supplementary Material

# Supplementary Figures and Tables

**Table 1: Host defense mechanisms**

| **Ligand** | **Type** | **Site on CR3** | **Site on ligand** | **Function** | **Cross-talk** |  |  |
| --- | --- | --- | --- | --- | --- | --- | --- |
| iC3b | Opsonin | α_M_I-domain, K_D_ 1.2 μM  MIDAS and surrounding AA, D140, S142, S144, T209, D242, D248, Y252,  P147-R152, P201-K217, F246-Y252, ^232^NAFKILVVITDGEK^245^ βA- α1, α3- α4, βD-α5 and βE- α6  β-propeller (D398-A402, L412-419, Y426-434, F435-443, S444-451), β_2_ (DXSXS) esp. D134, Ser136 | TED, C345c, MG7 | Phagocytosis, induction of tolerance | TGF-β, IL-10a | Recombinant α_M_I, CR3 transfected HEK293, monocytes, anti-CD11b mAbs, mutational studies, negative-stain EM, ITC studies, FACS immunoprecipitation, adhesion assays, SPR | (1–21) |
| C3d/C3dg | Opsonin | α_M_I-domain, MIDAS R208, E178, E179, L205, L206, G143, I145; K_D_ C3dg 1 μM | TED  D1245, K1217, R1254 | Phagocytosis in PNH (paroxysmal nocturnal hemoglobinuria) of erythrocytes | - | Recombinant α_M_I, crystal structure of C3d with α_M_I, SPR, phagocytosis assay using PBMCs | (4,10) |
| C3(H_2_O)  iC3 | Opsonin | - | - | Tethering of PMN and platelets | - | Quartz crystal microbalance measurement of interaction C3 and CD11b, recombinant CD11b, CR3 transfected CHO, anti-CD11b mAb | (22) |
| Del-1 | Developmental endothelial locus-1 | Competition with iC3b |  | Impairing binding of iC3b to Mac-1 and reduced phagocytosis |  | ELISA with immobilized CR3, binding of immobilized Del-1 to CR3 transfected CHO and RAW264.7, anti-CD11b mAbs  macrophages from wildtype and CD11b^‑/-^ mice | (23) |
| FH | Host defense regulator | Overlapping with iC3b | CCP6-7, CCP18-20 | Neutrophil activation, PMN polarization, H_2_O_2_ and lactoferrin release | C5a, TNFα | CR3 transfected CHO, PMNs, flow cytometry, anti-CD11b mAb | (24–26) |
| LL-37 | Endogenous antimicrobial peptide | α_M_I, competition with NIF | Residues 18-37 | Increasing phagocytosis of bacteria | - | CR3 transfected HEK293, recombinant, ^125^I-lableled α_M_I, SPR, BLI | (27,28) |
| Platelet factor 4 | Cytokine | α_M_I | Residues 12-26, 57-70, 58-66, 61-69 | Neutrophil activation, phagocytosis, integrin clustering | - | CR3 transfected HEK293, recombinant α_M_I, BLI, adhesion assays  macrophages from wildtype and CD11b^‑/-^ mice | (29) |
| FcγRIIa | - | α_M_I (E253-R261), NIF overlapping, divalent cation dependent, cis ligation | Silalic acid (N64, N145) | Antibody-dependent cytotoxicity, migration, immunological synapse formation | - | PMNs, CR3 transfected HEK293, immunoprecipitation, FACS,  resonance energy transfer studies and colocation via microscopy  wildtype and CD18^‑/-^ mice | (30–32) |
| FcγRIIIb (CD16) | Fc receptor | Lectin domain | - | Synergistic respiratory burst upon coligation of IgG and iC3b | Outside-in activation Co-ligation of CR3 and FCγRIII leads to rearrangement of actin and tyrosine phosphorylation of FcγRII | PMNs, CR3 transfected HEK293, immunoprecipitation, FACS,  resonance energy transfer studies and colocation via microscopy  wildtype and CD18^‑/-^ mice | (30–32) |
| SLAMF7 |  | - | - | Phagocytosis | - | Binding was shown on cells  immunoprecipitation, anti-CD11a mAb, anti-CD11b mAb, anti-CD11c mAb, anti-CD18 mAb | (33) |

**Table 2: Defense against pathogens**

| **Ligand** | **Type** | **Site on CR3** | **Site on ligand** | **Function** | **Cross-talk** |  |  |
| --- | --- | --- | --- | --- | --- | --- | --- |
| Neutrophil inhibitor factor (NIF) | Hockworm protein | α_M_I (MIDAS), G143, E178, E179, D149, R208 P147-R152, P201-K216, D248-R261 competition with ICAM-1, fibrinogen | - | Blocking adhesion of neutrophils to vascular endothelium | - | Recombinant α_M_I, homolog-scanning mutagenesis to α_X_I-domain, anti-CD11a mAb, anti-CD11b mAb, CR3 transfected CHO, PMNs, | (20,34–37) |
| LPS | Bacterial PAMP | Not iC3b, divalent cation dependent | Lipid A | - | - | Data before 1990. Binding was shown on PMNs:  Anti-CD11a mAbs, anti-CD11b mAbs. | (8,38) |
| *Klebsiella pneumonia* | Acylpolygalactosides | Divalent cation dependent | LPS like | - | - | Data from 1994. Binding was shown on cells:  PBMCs, anti-CD11a mAb, anti-CD11b mAb, anti-CD11c mAb, anti-CD18 mAb, anti-CR3 mAb | (39) |
| Zymosan | β-glucan cell wall of Saccharomyces | Lectin domain (residues 400-1092) | - | Induces phagocytosis and with co-ligation to iC3b respiratory burst | PKC mediated phosphorylation of β-subunit cytoplasmatic tail | Data from 1985. Binding was shown by using cells:  Human and bovine neutrophils and mononuclear cells, neutrophils forming rosettes upon binding, blocked by anti-CD11b mAb | (40) |
| β-glucan | (1,3)β-D-glucopyranosyl | Lectin domain (residues 400-1092), determined with chimeric CR3/CR4 receptors | - | Activates integrin, induces phagocytosis and with coligation to iC3b respiratory burst; coligation to fibrinogen induces NETosis | Phagocytosis of β-glucan does not require activation of monocyte | K562, CR3 transfected CHO, PBMCs, leukocytes, FACS, anti-CD11b mAb | (41–44) |
| Double-stranded RNA | Viral PAMP | - | - | NOX2 activation, production of ROS, TNF-𝛼, IL-12p40, IFN-𝛽 | MAPK, NF-𝜅B | Binding was shown by using mice:  CD11b^-/-^ mice, peritoneal macrophages, RAW264.7 cells anti-CD11b mAb | (45) |
| Laminarin |  | Competing with N-acetyl-D-glucosamine | - | - | - | Competition of binding from epithelial cells to purified CR3, anti-CD11b mAb, anti-CD11c mAb | (46) |
| N-Acetyl-D-glucosamine |  | Lectin domain (residues 400-1092), competing with FcγRIIIB, iC3b, Laminarin, Glucos-6-P | - | - | - | Competition of binding from epithelial cells to purified CR3, anti-CD11b mAb, anti-CD11c mAb | (42,46) |
| Mannose-6-P |  | Competing with N-acetyl-D-glucosamine | - | - | - | Competition of binding from epithelial cells to purified CR3, anti-CD11b mAb, anti-CD11c mAb | (42,46) |

**Table 3: Pathogen evasion**

| **Ligand** | **Type** | **Site on CR3** | **Site on ligand** | **Function** | **Cross-talk** |  |  |
| --- | --- | --- | --- | --- | --- | --- | --- |
| *Staphylococcus aureus* Leukocidin GH | Pore-forming toxin | α_M_I-domain: S144, S142, T209 (MIDAS), salt bridge E244, R208, polar contacts R208, van der Waals contacts F246, hydrophobic interaction P249. Hydrogen bond S277, L205 | LukH: E323 (MIDAS), R294, G324 (salt bridge), H188, Y321 (polar contacts), D114, H188, Y321 (van der Waals), W187 (hydrophobic interactions) Y314, D316 (hydrogen bond), LukG: R66 (hydrogen bond) | Pore formation, virulence | - | PMNs, CR3 transfected HEK293, recombinant α_M_I, SPR, BLI crystal structure | (47,48) |
| *Streptococcus pneumoniae* - Pneumolysin | Pore-forming toxin, cytolysin | sLe^x^ on α_M_I | - | Pore formation | - | SPR using recombinant CR3, unglycosilated recombinant α_M_I-domain and CR3 lacking sLex | (49) |
| *Mycobacterium tuberculosis* and *smegmatis* | - | α_M_I (not iC3b site), C-terminal for M.tuberculosis, competes with laminarin and NADG | - | Binding and internalisation of M. tuberculosis | - | Data from 1996:  anti-CD11b mAb, CR3 transfected CHO, PBMCs | (50) |
| *Neisseria gonorrhoeae* | Pili and surface glucan | Activated α_M_I, lectin domain, cooperative with FH | Pilus glycan | Host evasion: internalization without inflammation | - | CR3 transfected CHO, flow cytometry, anti-CD11b mAb, co-immunoprecipitation, ELISA | (25,51,52) |
| Group B Steptococci |  | - | - | Phagocytosis | - | Data from 1992. Binding was shown on cells:  PU5-1.8 mouse macrophage-like cell line, anti-CD11b mAb, anti-CD18 mAb | (53) |
| *Porphyromonas gingivalis* - Fimbrillin | Fimbrae protein | - | - | Downregulation IL-12, increased pathogenicity | Erk1/2 | Binding was suggested by using mice:  mouse peritoneal macrophages, anti-CD11a mAb, anti-CD11b mAb, anti-CD11c mAb, anti-CD18 mAb, immunoprecipitation | (54,55) |
| *Bordetella pertussis* | Filamentous hemagglutinin, pertussis toxin and adenylate cyclase | Binding of CyaA Ca^2+^ dependent, not Mg^2+^ dependent, ⭢ binding is α_M_I-domain independent? | - | Reduced expression of IL-12, macrophage adhesion | - | Human macrophages, PBMCs, THP-1, U937, anti-CD11a mAb, anti-CD11b mAb, anti-CD11c mAb, anti-CD18 mAb, adhesion assays, flow cytometry, murine macrphages | (56–60) |
| *Bacillus anthracis* - BclA | Divalent cation | - | - | Spore uptake | - | CD11b^-/-^ mice, CR3 transfected HEK, anti-CD11b mAb | (61) |
| *Streptococcus pneumoniae* - RrgA | Pilus adhesin | α_M_I | - | Increased phagocytosis, virulence | - | Anti-CD11b mAb, purified human CR3, THP-1, FACS, murine macrophages | (62) |
| *Franciscella tularensis* | Facultative intracellular pathogen | - | - | C3 opsonized results in limited inflammasome priming and pro-inflammatory cytokine production | RasGAP, leads to inhibition of Ras-ERK | Monocytes and macrophages, anti-CD11b mAb | (63) |
| *Borrelia burgdorferi* – OspA, OspB | OpsA, OpsB surface proteins | Not overlapping to iC3b | - | - | - | Purified CR3, CR3 affinity purification of OspA/OspB, immunofluorescence | (64) |
| *Leishmania sp.* - gp63 | Obligate intracellular parasites | - | Residues 365-386, 252-255 | - | - | Data from 1992 and before:  Human monocytes, anti-CD11a mAbs, anti-CD11b mAb, anti-CD11c mAb, anti-CD18 mAb | (65–67) |
| HIV-1 |  | - | - | Decreased inflammatory and antiviral response in iDCs | - | Binding was shown on PBMCs, anti-CD11b mAb, anti-CD18 mAb, ELISA | (68,69) |
| Herpes simplex 2 |  | Opsonized with iC3b and without | - | Opsonized HSV2 increased infection of DC | - | Binding was shown by using cells:  Silencing of CD11b in DCs, PBMCs from healthy donors and SLE patients | (70) |
| Hantavirus | Zoonotic pathogen | In competition to heparin | - | Increased virulence by NETosis causing severe renal and pulmonary pathology | - | Binding was only shown by using cells:  PBMCs, anti-CD11a mAb, anti-CD11b mAb, anti-CD11c mAb, anti-CD18 mAb, FACS, ELISA, CD18^-/-^ mice | (71) |
| *Candida albicans* | Dimorphic fungus | Competed by vitronectin, fibrinogen (α_M_I-domain) and NADG, β-glucan (lectin) | Β-glucan | Candida killing by co-ligation with FH | - | Data from 1996 and before. Binding was shown on lymphocytes:  anti-CD11b mAb, anti-CD11c mAb, anti-CD18 mAb. | (72,73) |
| *Blastomyces dermatitis* | Dimorphic fungus | Via same binding site like LPS, divalent cation dependent | Probabpy via the WI-1 surface protein | Increased phagocytosis, virulence | - | Data from 1995. Binding was shown on monocytes:  anti-CD11a mAb, anti-CD11b mAb, anti-CD18 mAb, anti WI-1 mAb. | (74) |
| *Histoplasma capsulatum* | Dimorphic fungus | Divalent cation dependent, not via lectin binding site | - | Increased phagocytosis, virulence, induction of ROS | - | Data from 1987. Binding was shown on monocytes:  anti-CD11a mAb, anti-CD11b mAb, anti-CD18 mAb. | (75) |

**Table 4: Recognition of host damage patterns**

| **Ligand** | **Type** | **Site on CR3** | **Site on ligand** | **Function** | **Cross-talk** |  |  |
| --- | --- | --- | --- | --- | --- | --- | --- |
| Albumin (also denatured), Ovalbumin | Globular protein | - | Unfolded parts and flexible loops containing acidic residues | - | - | Binding was shown on cells:  U937, THP-1, anti CD11b mAb, anti-CD18 mAb | (76–78) |
| DNA |  | Competition with heparin and fibrinogen | - | ROS production | - | Data from 1997:  anti-CD11b mAb, purified CR3, CR3 transfected CHO | (79) |
| Myelin basic protein (MBP), galitamer acetate (GA) |  | α_M_I (MIDAS), divalent cation dependent |  | Phagocytosis of denatured myelin |  | Monocytes, CR3 transfected R562, recombinant α_M_I, SPR, ELISA | (80–82) |
| β-amyloid | Component of amyloid plaques in alzheimer's disease | - | - | NO release, decreased phagocytic activity, increased b-amyloid degradation, | - | Binding was shown on cells isolated from post mortem tissues from patients, anti-CD11b mAb, anti-CD18 mAb, FACS, immunoprecipitation, CR3^‑/-^ mice, rat microglia | (83–87) |
| α-synuclein | Neuronal protein | - |  | Translocation of p47^phox^ ⭢ NOX2 activation, ROS production, CR3 involved in synucleopathies? | Possibly Rho signaling pathway | Binding was shown by using mice derived BV2 microglial cells, anti-CD11b, FACS, immunoprecipitation CD11b^‑/-^ mice, CR3^‑/-^ mice | (88–90) |
| CD157 | glycosylphosphatidylinositol-anchored molecule | - | - | Neuroinflammation | - | Binding was suggested in a PD model mice, anti-CD11b mAb, anti-CD18 mAb | (91) |
| 2,5-Hexanedione | Active metabolite of n-Hexane | - | - | Translocation of p47^phox^, NOX2 activation, ROS production | Src-Erk pathway | Binding was shown by using mice derived BV2 microglial cells, anti-CD11b | (92) |
| Diesel exhaust | Air pollution | - | - | NOX2 activation, ROS production | - | Binding was suggested by comparing WT and CR3 kockout mice, anti-CD11b | (93) |
| HMGB1 (amphotherin) | Chromatin binding protein | - | - | Increases TNF-α, IL-1β and NO formation ⭢ neurodegeneration | NF-𝜅B | Binding was suggested by comparing WT and CR3 kockout mice, co-immunoprecipitation | (94) |

**Table 5: CR3 as modulator of leukocyte function**

| **Ligand** | **Type** | **Site on CR3** | **Site on ligand** | **Function** | **Cross-talk** |  |  |
| --- | --- | --- | --- | --- | --- | --- | --- |
| ICAM-1 (CD54) | Adhesion molecule | α_M_I, DXSXS in β_2_, not competing with fibrinogen, cis-ligation sICAM-1 6.45 ± 0.58 µM | 3. Ig domain | Leukodiapedesis – expression only in inflammatory sites | - | Recombinant α_M_I, purified CR3, PMNs, mutational studies, SPR, anti-CD11a mAb, anti-CD11b mAb, ELISA, immunoprecipitation | (7,95–98) |
| ICAM-2 (CD102) | Adhesion molecule | α_M_I, not competing with iC3b, fibrinogen and FX | 1. Ig domain | T cell aggregation, NK cell migration and cytotoxicity | - | Data from 1995:  CR3 transfected CHO, purified CR3, THP-1, anti-CD11a mAb, anti-CD11b mAb, anti-CD18 mAb, flow cytometry | (99,100) |
| ICAM-4 | Adhesion molecule | Divalent cation dependent | Ig domains D1 and D2 | - | - | CR3 transfected COS-7, anti-CD11b mAb | (101) |
| JAM-C  (junctional adhesion molecule) | Adhesion molecule | α_M_I, competing with fibrinogen | - | Platelet-neutrophil interaction, transepithelial migration | - | Purified CR3, purified α_M_I, anti-CD11a mAb, anti-CD11b mAb, ELISA | (102,103) |
| CD147 (Basigin) | Transmembrane glycoprotein, IgG superfamily | - | - | - | - | Recombinant CR3, monocytes, ELISA, adhesion assay under static and dynamic conditions, anti-CR3, anti-CD147  Wildtype and CR3^‑/-^ mice | (104) |
| RAGE (AGER) | IgG family | α_M_I, competing with fibrinogen and HMWK | - | Neutrophil extravasation into peritoneum | - | Purified CR3, CR3 transfected K562, recombinant α_M_I | (105) |
| Thy-1 (CD90) | Activation-associated adhesion molecule | α_M_I, not competing with ICAM-1 | - | Neutrophil adhesion to endothelial cells, migration, accumulation in skin lesions | TNF-α | PMNs from healthy and psoriatic patients, anti-CD11b mAb, anti-Thy-1 mAb, flow cytometry | (106,107) |
| SIRPα  (signal regulatory protein α) | IgG family | α_M_I, K_D_ 1.4 µM | Ig1-2-3 ectodomain, Residues 87-98, 147-155, 150-158, 240-257, 33-41, 114-122 | Macrophage fusion, anti-phagocytosis signal | - | CR3 transfected HEK293, recombinant α_M_I, anti-CD11b mAb, BLI, immunoprecipitation, flow cytometry | (108) |
| CD40L | TNF superfamily | α_M_I (residues 162-170, K_D_ 200-670 nM), distinct of fibrinogen | Distinct from CD40 and GPIIb/IIIa binding site | Leukocyte recruitment in atherosclerosis |  | Recombinant α_M_I, CR3 transfected CHO, dynamic and static cell adhesion assays, flow cytometry, SPR | (109) |
| Azurocidin and Elastase | Antimicrobial protein | - | Catalytic domain | - | - | Data from 1996:  anti-CD11b mAb, purified CR3, immunoprecipitation, ELISA | (110) |
| Myeloperoxidase | Peroxidase | - | - | - | - | Data from 1997. Binding was shown on monocytes, ELISA, anti-CD11b mAb, anti-CD18 mAb | (111) |
| Pleiotrophin | Basic heparin binding brain mitogen | α_M_I | Two thrombospondin type-1 repeat domains which are flanked by unstructured highly basic termini form Pleiotrophin Four clusters contain α_M_I binding domains. Large basic surfaces | Macrophage migration, MAP kinase activation, phosphorylation of Erk1/2 | - | Anti-CD11b mAb, CR3 transfected HEK293, recombinant α_M_I, BLI, ELISA | (112) |
| Dynorphin A | Endogenous opioid peptide, cationic | α_M_I | 9 different 9-mer, all bind with different affinity:  YGGFLRRIR  FLRRIRPKL  PKLKWDNQK  KWDNQKRYG  NQKRYGGFL  RYGGFLRRQ  GFLRRQFKV  RRQFKVVGG | Enhanced phagocytosis | - | Recombinant α_M_I, CR3 transfected HEK293, HEK with CD11b without I domain, anti-CD11b mAb, anti-CD18 mAb, ELISA | (113) |
| Pro-MMP2, Pro-MMP9 | Matrix metalloproteinases, gelatinases | αMI | Catalytic domain | Suggested to be involved in neutrophil migration | - | PMNs, recombinant α_M_I, purified CR3, CR3 transfected L929, anti-CD11a mAb, anti-CD11b mAb, anti-CD18 mAb | (114,115) |
| DC-SIGN | C-type lectin | Le^X^ on CD11b, binds only to CR3 on PMNs | Mutation studies indicate that the c-type lectin domain is involved iin binding to CR3 | DC maturation, cytokine production | - | PMNs, immunoblot, immunoprecipitation, anti-CD11b mAb, purified CR3, immature DCs, α1-3,4-fucosidase (targets Lewis^X,^ removes fucose moiety required for DC SIGN binding) | (116) |

**Table 6: Leukocyte migration on extracellular matrix**

| **Ligand** | **Type** | **Site on CR3** | **Site on ligand** | **Function** | **Cross-talk** |  |  |
| --- | --- | --- | --- | --- | --- | --- | --- |
| Thrombospondin | ECM | - | - | ROS secretion | - | Data from 1989 and binding was on PMNs from healthy donors and LAD patients, anti-CD11a mAb, anti-CD11b mAb, anti-CD11c mAb, anti-CD18 mAb | (117) |
| Vitronectin |  | α_M_I, overlapping with fibrinogen, divalent cation dependent | Not RDG, not somatomedin B domain | - | - | PMNs from healthy donors and LAD patients, recombinant CR3, CR3 transfected CHO, anti-CD11a mAb, anti-CD11b mAb, anti-CD11c, anti-CD18 mAb | (117,118) |
| Fibrinogen | Zymogen glycoprotein | α_M_I (βD-α5 loop, residues 245-261), not MIDAS, not cation dependent, overlapping with iC3b, but not directly competing, beta I-like domain is involved in binding (residues 388-395), K_D_ 3.98 ± 0.86 µM | γ-chain (residues 192-286, 377-395), β-C domain (residues 247-255) | PMN migration of fibrinogen, proinflammatory, involvement  in sickel cell anemia, muscle dystrophy | IL-6, IL-1β, TNFα | CR3 expressing HEK293, recombinant α_M_I, THP-1, U937, PBMC, PMNs,  anti-CD11a mAb, anti-CD11b mAb, anti-CD11c mAb, anti-CD18 mAb, homology between β_2_-integrins, SPR, immunoprecipitation, ELISA,  wildtype and CD11b^‑/-^ mice | (7,9,96,98,119–140) |
| Fibrinogen-420 | Alternative splicing | Competes with NIF | αEC | - | - | CR3 transfected HEK cells, recombinant α_M_I, anti-CD11b mAb, anti-CD18 mAb | (141) |
| Fibronectin | Glycoprotein | α_M_I | - | Reduces migration | - | CR3 transfected HEK293, recombinant α_M_I, PMNs from healthy donors and LAD patients, anti-CD11a mAb, anti-CD11b mAb, anti-CD11c mAb, anti-CD18 mAb, ELISA | (117,142,143) |
| Collagen | ECM | - | GFOGER of collagen | ROS secretion, PMN migration in inflamed tissue | - | PMNs, recombinant α_M_I, anti-CD11a mAb, anti-CD11b mAb, anti-CD11c mAb, anti-CD18 mAb, ELISA | (143–145) |
| Undulin | ECM |  | - | - | - | Data from 1995 and binding was shown by using cells:  PMNs, anti-CD11a mAb, anti-CD11b mAb, anti-CD11c mAb, anti-CD18 mAb | (144) |
| Laminin | ECM |  | - | ROS secretion | - | Data from 1995 and before. Binding was shown by using cells:  PMNs, anti-CD11a mAb, anti-CD11b mAb, anti-CD11c mAb, anti-CD18 mAb, immunoprecipitation | (143,144,146) |
| Lumican | ECM | - | - | - | - | Binding was shown on PMNs, migration assays, FACS, adhesion assays, anti-CD11a mAb, anti-CD11b mAb, anti-CD18 mAb | (147) |
| Mindin | Spondin 2, ECM protein, PRM | α_M_I | FS domain | Opsonization, phagocytosis | Syk, NF-kB p65 | CR3 transfected HEK293, anti-CD11b mAb, anti-CD18 mAb, immunoprecipitation, wildtype and mindin^‑/-^ mice | (148,149) |
| CCN1 and CCN2 | Connective tissue growth factor, matricellular signaling molecules | α_M_I | C-terminus of CCN1 (SSVKKYRPKYCGS) | Expression and secretion of proinflammatory mediators | NF-kB | PBMCs, THP-1, recombinant α_M_I, anti-CD11b mAb, anti-CD11c mAb, anti-CD18 mAb, ELISA, wildtype and CR3^‑/-^ mice | (150–152) |
| CEP | Oxidized ECM | α_M_I | - | Macrophage migration | - | CR3/CR4/LFA-1-transfected HEK293 cells, recombinant α_M_I-, α_L_I-, α_X_I-domains, SPR  wild type and β_2_-deficient mice | (153) |
| Plasminogen | Zymogen serine protease (plasmin) | α_M_I, immobilized competes with P2 (fibrinogen derived), soluble (K_D_ of 0.3 ±0.01 µM) by tranexamic acid | Kringle domains 1, 2, 4, and 5 | - | - | CR3/I less CR3 transfected HEK293 recombinant α_M_I, SPR, anti-CD11b mAb | (129) |
| Angiostatin | Fragment of Plasminogen, K1-3, K1-4 | α_M_I, further binding sites possible. | Kringle domain 4 | Inhibition of neutrophil extravasation, reduction of NFκB activation and TF expression |  | Purified CR3, purified α_M_I, anti-CD11a mAb, anti-CD11b mAb, anti CD18 mAb | (154) |
| Lipoprotein(a) | Apo(a) has a high homology with kringle 4 of plasminogen | α_M_I | Apo(a) domain, binding is upregulated by preincubation with homocysteine | Increased NFκB activation and TF expression, increased transmigration/cell recruitment |  | Purified CR3, THP-1, anti-CD11a mAb, anti-CD11b mAb | (155) |
| uPAR | GPI glycoprotein | W4 blade of β-propeller (residues 424-440) | - | Priming, leukocyte recruitment and migration, cis interaction; enhances fibrinogen binding and plasminogen activation | FAK, MAP kinase | CR3 transfected HEK293 and CHO, U937, HL60, THP-1, chimer recombinant CD11b, immunoprecipitation | (156–159) |
| tPA (tissue plasminogen activator) | Serine protease | Competes with NIF | CR3, fibrin and tPA form adhesive complex | Enhanced fibrin binding, aggregation and interaction with Annexin A2 | ILK, NF-κB | CR3 transfected HEK293, anti-CD11b mAb, ELISA, immunoprecipitation, wild type and CD11b^‑/-^ mice | (160,161) |
| Annexin A2 | tPA receptor | - | - | - | Clustering of CD11b and outside-in signaling, ILK, NF-kB | Binding was suggested by using knockout mice:  co-immunoprecipitation, anti-CD11b mAb, wild type and CD11b^‑/-^ mice | (160) |
| NB1 (CD177) | Human member of Ly6/uPar family | - | - | ROS production, neutrophil activation | - | CR3 transfected cells, recombinant CD11b, anti CD11b mAb, anti-CD18 mAb, anti-CD11a mAb, anti-CD11c mAb, SPR, immunoprecipitation | (162) |
| LRP1 | Scavenger receptor | α_M_I (Res. 162-170, opposite MIDAS) competition with fibrinogen | - | Detachment of macrophage through internalization | Complex of CR3, tPA, fibrin and PAI-1 | CR3 transfected HEK293, U937, recombinant α_M_I, immunoprecipitation, confocal microscopy, SPR, ELISA, wild type and CD11b^‑/-^ mice | (161,163,164) |

**Table 7: Leukocyte interaction with homeostasis and thrombi**

| **Ligand** | **Type** | **Site on CR3** | **Site on ligand** | **Function** | **Cross-talk** |  |  |
| --- | --- | --- | --- | --- | --- | --- | --- |
| GPIbα |  | α_M_I (residues 142-144, 208, 209, 242, 244, 273, 279), competing with heparin, fibrinogen, glucosamine | Leucine- rich N-terminal region (residues 201-268) | Adhesion and trans-platelet migration, pro-inflammatory and pro-thrombotic, NETosis | PKCδ, Foxp1 TF | THP-1, U937, CR3 transfected HEK293 and K562, recombinant α_M_I, chimeric LFA-1 with α_M_I , site-directed mutagenesis; NMR, crystal structure of mouse α_M_I-domain and mouse GPIbα, SPR, wild type and CD11b^‑/-^ mice | (120,121,165–167) |
| Fucoidan | Fucosylated proteoglycans | Divalent cation dependent | Sulfates essential for binding | Elastase release, reduced mobilization of bone marrow nucleated cells | - | Purified CR3, bone-marrow nucleated cells from wildtype and CD11b^‑/-^ mice, anti-CD11b mAb | (46,168) |
| CD44v3 |  | Divalent cation dependent | Heparan sulfate, Heparinase treatment almost  completely inhibited the binding | PMN-Epithelial Adhesion | - | Purified CR3, PMNs, ELISA, anti-CD11b mAb, anti-CD11c mAb | (169) |
| Heparin  heparan | Glycosamin-glycan | α_M_I, competes with fibrinogen, FX, ICAM-1, iC3b | Sulfates essential for binding | - | - | THP-1, PMNs, purified CR3, CR3 transfected CHO, anti-CD11b mAb | (79,165,170,171) |
| Glucosamine |  | α_M_I (residues 216, 220, 221, 217, 213, K_D_ 100 µM) | - | Anti-thrombotic, inhibits ligation to GPIbα | - | Recombinant α_M_I, CR3 transfected HEK293, THP-1. SPR | (166) |
| FX (not FXa) | Zymogen serine protease of coagulation system | Not mainly mediated by α_M_I, but iC3b competing and Ca^2+^ dependent | Three distinct sequences surrounding the catalytic site (residues 366-375, 422-430, 238-246) | Gets activated to FXa by degranulation of activated leukocytes (cleavage by cathepsin G) | - | U937, THP-1, PBMCs, wild type and I-less CR3 transfected HEK293, recombinant α_M_I, anti-CD11a mAb, anti-CD11b mAb, anti-CD11c mAb | (3,98,172–174) |
| Kininogen | HMWK und HMWKa | α_M_I, overlapping with ICAM-1 and fibrinogen, divalent cation dependent, HMWKa, K_D_ 62 nM, interaction of soluble ICAM-1 with immobilized CR3 was blocked by HK (IC_50_: 215 nM), HKa (IC_50_: 100 nM), domain 5 (IC_50_: 150 nM), domain 3 (IC_50_: 500 nM) | Domain 3, mainly the C-terminus of domain 5 (residues 475-497, 440-455) | Elastase release, formation of GPIbα:CR3, release of cytokines (TNF-α, IL-1β, IL-6) and chemokines (IL-8 and MCP-1), in complex with uPAR, LFA-1 and gC1qR? | IL-1β release via NFkB, p38 and JNK | U937, PMNs, CR3 transfected K562 and HEK293, purified CR3, anti-CD11b mAb, ELISA | (119,167,175–179) |
| Thrombomodulin | Anticoagulant | - | Thrombomodulin domain 3 is required for binding to CR3. | Interferes with ANCA binding, inhibition of neutrophil extracellular trap formation | - | Binding was shown on cells:  PBMCs, anti-CD11a mAb, anti-CD11b mAb, CR3^‑/-^ HL-60 | (180,181) |
| protein-C receptor | Soluble endothelial protein C-receptor (EPCR) | - | - | - | - | CR3 transfected CHO anti-CD11b mAb, static and dynamic adhesion assays | (182) |

**Table 8: Involvement of CR3 in additional interactions**

| **Ligand** | **Type** | **Site on CR3** | **Site on ligand** | **Function** | **Cross-talk** |  |  |
| --- | --- | --- | --- | --- | --- | --- | --- |
| Protein disulfide isomerase (extracellular) | Endoplasmatic reticulum and cell surface thiol isomerase |  | - | Neutrophil recruitment, regulates fibrinogen binding and integrin clustering | - | Recombinant CR3, SPR, immunoprecipitation, anti-CD11b mAb, anti CD18 mAb | (183) |
| BAP31 | B-Cell associated protein 31 | Binding was independent of α_M_I-domain | - | - | - | Purified CR3, anti-CD11b mAb, anti-CD11a mAb, anti-CD11c mAb, anti-CD18 mAb, ELISA, immunoprrecipitation | (184) |
| CD22 | Siglec-2 | Binds CD11b glycosylation, reduced upon neuramidase treatment | - | Not binding R77H (SLE) | - | Binding was suggested by using knockout mice:  wild type and CD11b^‑/-^ mice, anti-CD11b mAb, FACS | (185) |
| CD23 | C-type lectin | Inhibited by FX, divalent cation dependent | - | ROS production, release of proinflammatory cytokines (IL-1b, IL-6, TNFa) | - | Data from 1995:  CD11b transfected COS-7, isolated monocytes, anti-CD11b mAb, FACS | (186) |
| IL-13Rα1 | Cytokine receptor | W5 blade of β-propeller (453-511) CD11b leg (615-1033) | D1 (27-117), D2 (129-219), D3 (233-346) | Cis ligation as negative feedback reduced foam cell formation | - | CR3 transfected HEK293, immunoprecipitation, cell adhesion assay, anti-CD11b mAb | (187) |
| Steel | Used for stents | - | - | Binding of monocytes to steel stents can lead to restenosis | - | CR3 transfected CHO, monocytes, anti-CD11b mAb, anti-CD18 mAb | (188) |

**Table 9: CR3 as a potential therapeutic target**

| **Ligand** | **Type** | **Site on CR3** | **Site on ligand** | **Function** | **Cross-talk** |  |  |
| --- | --- | --- | --- | --- | --- | --- | --- |
| Imprime PGG | Soluble β-glucan | - | - | Activate anti-cancer innate immune effector functions | - | - | (189–192) |
| Gu-4 | Lactosyl- derivative | Lectin binding site | oligosaccharides | Inhibition of leukocyte adhesion and transendothelial migration | - | Binding was shown on THP-1 cells:  anti-CD11b mAb | (193) |
| Hydroxyethyl starch | Colloid for plasma replacement therapy | - | - | Reducing their migration, and chemotaxis of activated PMN | PI3K/Akt | Binding was shown on neutrophils, anti-CD11a mAb, anti-CD11b mAb, anti-CD18 mAb | (194) |
| **Antagonists** | | | | | | | |
| Abciximab | GPIIb/IIIa inhibitor | - | - | Blocking of different CR3 functions | - | Binding was shown on cells:  Monocytes, CR3 expressing THP-1, U937, anti-CD11b mAb | (195) |
| Leumidin | LMW anti-inflammatory drug | IC_50_ 5 µM | - | Inhibition of neutrophil adhesion | - | Data from 1996 and before. Binding was shown on cells:  PMNs, anti-CD11b mAb, anti-CD11c mAb, anti-CD11a mAb, anti-CD18 mAb | (196,197) |
| Covalent small molecule | LMW anti-inflammatory drug | Inhibiting CR3:iC3b, IC_50_ 0.14 µM | - | Anti-inflammatory by reduced neutrophil emigration | - | Purified CR3, PMNs, anti-CD11b mAb, anti-CD18 mAb, ELISA | (198) |
| Gupta group | Small molecule | Inhibited binding to fibrinogen, IC_50_ < 1 µM | - | - | - | CR3 transfected K562, anti-CD11b mAb, anti-CD18 mAb | (199) |
| Genentech and Roche | Small molecule | Antagonists to the I-like domain, inhibiting binding of iC3b and ICAM-1, IC_50_ 0.9 µM | - | - | - | CR3 transfected K562, SDS-PAGE assay of CR3 stabilization | (200) |
| Simvastatin | HMG-CoA-Reductase inhibitor | α_M_I-domain, MIDAS (S142, S144, T209) other amino acid residues are involved, which are not part of the MIDAS (G143, G207, F246) | Carboxylic acid | Inhibition of monocyte binding to iC3b | - | Crystal structure, SPR, recombinant α_M_I, anti-CD18mAb, CR3 transfected K562 | (201) |
| E/DDGW | Peptide (phage display) | Competes with MMP-9, IC_50_ 20 µM | - | - | - | Recombinant α_M_I, purified CR3, ELISA | (114) |
| CP[CFLLGC]C | Peptide (phage display) | Divalent cation dependent, IC_50_ 20 µM, competes with ICAM-1, vWF and collagen | - | Inhibits leukocyte adhesion to ICAM-1 | - | Purified CR3, recombinant α_M_I, anti-CD11a mAb, anti-CD11b mAb, anti-CD18 mAb | (202) |
| GYRDGYAGPILYN | Peptide (phage display) | Competes with ICAM-1, IC_50_ 30 µM | - | - | - | Purified CR3, recombinant α_M_I | (203) |
| **Agonists** | | | | | | | |
| 2-thioxothiazolidin-4-one | Small molecule | Competing with DDGW peptide, enhanced binding of fibrinogen and proMMP-9 | - | - | - | THP-1, recombinant α_M_I, ELISA | (204) |
| Leukoadherins | Small molecule | EC_50_ 10 - 40 µM; LA-1 EC_50_ 13.6 µM, increasing binding to fibrinogen | - | - | No effect on signaling, reducing chemotaxis and transendothelial migration | CR3 transfected K562, PBMCs, recombinant α_M_I, ELISA, immunoprecipitation, anti-CD11b mAb, anti-CD18 mAb  wild type, CR3^‑/-^, and CR3 knock in mice | (205–212) |

**References**

1. Ross G. Identification of a C3bi-specific membrane complement receptor that is expressed on lymphocytes, monocytes, neutrophils, and erythrocytes. *J Exp Med* (1982) **155**:96–110. doi:10.1084/jem.155.1.96

2. Ross G. Generation of three different fragments of bound C3 with purified factor I or serum. II. Location of binding sites in the C3 Fragments for Factors B and H, complement receptors , and bovine conglutinin. *J Exp Med* (1983) **158**:334–352. doi:10.1084/jem.158.2.334

3. Yalamanchili P, Lu C, Oxvig C, Springer TA. Folding and function of I domain-deleted Mac-1 and lymphocyte function-associated antigen-1. *J Biol Chem* (2000) **275**:21877–21882. doi:10.1074/jbc.M908868199

4. Bajic G, Yatime L, Sim RB, Vorup-Jensen T, Andersen GR. Structural insight on the recognition of surface-bound opsonins by the integrin i domain of complement receptor 3. *Proc Natl Acad Sci U S A* (2013) **110**:16426–16431. doi:10.1073/pnas.1311261110

5. Kamata T, Wright R, Takada Y. Critical Threonine and Aspartic acid Residues within the I Domains of beta2 integrins for interaction with Intercellular adhesion Molecule 1 (ICAM-1) and C3bi. *J Biol Chem* (1995) **270**:12531–12535.

6. Michishita M, Videm V, Amin Arnaout M. A novel divalent cation-binding site in the a domain of the β2 integrin CR3 (CD11b/CD18) is essential for ligand binding. *Cell* (1993) **72**:857–867. doi:10.1016/0092-8674(93)90575-B

7. Diamond MS, Garcia-Aguilar J, Bickford JK, Corbi AL, Springer TA. The I domain is a major recognition site on the leukocyte integrin Mac-1 (CD11b/CD18) for four distinct adhesion ligands. *J Cell Biol* (1993) **120**:1031–1043. doi:10.1083/jcb.120.4.1031

8. Wright SD, Levin SM, Jong MTC, Chad Z, Kabbash LG. CR3 (CD11b/CD18) expresses one binding site for Arg-Gly-Asp-containing peptides and a second site for bacterial lipopolysaccharide. *J Exp Med* (1989) **169**:175–183.

9. Yakubenko VP, Lishko VK, Lam SCT, Ugarova TP. A molecular basis for integrin αMβ2 ligand binding promiscuity. *J Biol Chem* (2002) **277**:48635–48642. doi:10.1074/jbc.M208877200

10. Lin Z, Schmidt CQ, Koutsogiannaki S, Ricci P, Risitano AM, Lambris JD, Ricklin D. Complement C3dg-mediated erythrophagocytosis: Implications for paroxysmal nocturnal hemoglobinuria. *Blood* (2015) **126**:891–894. doi:10.1182/blood-2015-02-625871

11. Xu S, Wang J, Wang J-H, Springer TA. Distinct recognition of complement iC3b by integrins α X β 2 and α M β 2. *Proc Natl Acad Sci* (2017) **114**:3403–3408. doi:10.1073/pnas.1620881114

12. Taniguchi-Sidle A, Isenman DE. Mutagenesis of the Arg-Gly-Asp triplet in human complement component C3 does not abolish binding of iC3b to the leukocyte integrin complement receptor type III (CR3, CD11b/CD18). *J Biol Chem* (1992) **267**:635–643.

13. Arnaout MA, Todd RF, Dana N, Melamed J, Schlossman SF, Colten HR. Inhibition of phagocytosis of complement C3- or immunoglobulin G-coated particles and of C3bi binding by monoclonal antibodies to a monocyte-granulocyte membrane glycoprotein (Mo1). *J Clin Invest* (1983) **72**:171–179. doi:10.1172/JCI110955

14. Jensen R, Bajic G, Sen M, Springer T, Vorup-Jensen T, Andersen G. Complement receptor 3 forms a compact high affinity complex with iC3b. *bioRxiv* (2020) doi:10.1101/2020.04.15.043133

15. Zhang L, Plow EF. Overlapping, but not identical, sites are involved in the recognition of C3bi, neutrophil inhibitory factor, and adhesive ligands by the α(M)β2 integrin. *J Biol Chem* (1996) **271**:18211–18216. doi:10.1074/jbc.271.30.18211

16. Li Y, Zhang L. The Fourth Blade within the β-Propeller Is Involved Specifically in C3bi Recognition by Integrin αMβ2. *J Biol Chem* (2003) **278**:34395–34402. doi:10.1074/jbc.M304190200

17. Ueda T, Rieu P, Brayer J, Arnaout MA. Identification of the complement iC3b binding site in the beta 2 integrin CR3 (CD11b/CD18). *Proc Natl Acad Sci* (1994) **91**:10680–10684. doi:10.1073/pnas.91.22.10680

18. Zhang L, Plow EF. Amino acid sequences within the α subunit of integrin αmβ2 (MAC-1) critical for specific recognition of C3bi. *Biochemistry* (1999) **38**:8064–8071. doi:10.1021/bi990141h

19. Bajt ML, Godmann T, McGuire S lea. Beta 2 (CD18) mutations abolish ligand recognition by I domain integrins LFA-1 (alpha L beta 2, CD11a/CD18) and MAC-1 (alpha M beta 2, CD11b/CD18). *J Biol Chem* (1995) **270**:94–98. doi:10.1074/jbc.270.1.94

20. Goodman TG, Lynn Bajt M. Identifying the Putative Metal Ion-dependent Adhesion Site in the beta2 (CD18) Subunit Required for alphaLbeta2 and alphaMbeta2 Ligand Interactions. *J Biol Chem* (1996) **271**:23729–23736.

21. McGuire SL, Bajt ML. Distinct ligand binding sites in the I domain of integrin αMβ2 that differentially affect a divalent cation-dependent conformation. *J Biol Chem* (1995) **270**:25866–25871. doi:10.1074/jbc.270.43.25866

22. Hamad OA, Mitroulis I, Fromell K, Kozarcanin H, Chavakis T, Ricklin D, Lambris JD, Ekdahl KN, Nilsson B. Contact activation of C3 enables tethering between activated platelets and polymorphonuclear leukocytes via CD11b/CD18. *Thromb Haemost* (2015) **114**:1207–1217. doi:10.1160/TH15-02-0162

23. Mitroulis I, Kang YY, Gahmberg CG, Siegert G, Hajishengallis G, Chavakis T, Choi EY. Developmental endothelial locus-1 attenuates complement-dependent phagocytosis through inhibition of Mac-1-integrin. *Thromb Haemost* (2014) **111**:1004–1006. doi:10.1160/TH13-09-0794

24. DiScipio RG, Daffern PJ, Schraufstätter IU, Sriramarao P, DiScipio RG, Daffern PJ, Schraufstätter IU. Human polymorphonuclear leukocytes adhere to complement factor H through an interaction that involves alphaMbeta2 (CD11b/CD18). *J Immunol* (1998) **160**:4057–66.

25. Agarwal S, Ram S, Ngampasutadol J, Gulati S, Zipfel PF, Rice PA. Factor H Facilitates Adherence of Neisseria gonorrhoeae to Complement Receptor 3 on Eukaryotic Cells . *J Immunol* (2010) **185**:4344–4353. doi:10.4049/jimmunol.0904191

26. Losse J, Zipfel PF, Józsi M. Factor H and Factor H-Related Protein 1 Bind to Human Neutrophils via Complement Receptor 3, Mediate Attachment to Candida albicans , and Enhance Neutrophil Antimicrobial Activity . *J Immunol* (2010) **184**:912–921. doi:10.4049/jimmunol.0901702

27. Lishko VK, Moreno B, Podolnikova NP, Ugarova TP. Identification of Human Cathelicidin Peptide LL-37 as a Ligand for Macrophage Integrin αMβ2 (Mac-1, CD11b/CD18) that Promotes Phagocytosis by Opsonizing Bacteria. *FASEB J* (2016) **2016**:39–55.

28. Zhang X, Bajic G, Andersen GR, Christiansen SH, Vorup-Jensen T. The cationic peptide LL-37 binds Mac-1 (CD11b/CD18) with a low dissociation rate and promotes phagocytosis. (2016) **1864**:471–478. doi:10.1016/j.bbapap.2016.02.013

29. Lishko VK, Yakubenko VP, Ugarova TP, Podolnikova NP. Leukocyte integrin Mac-1 (CD11b/CD18, alphaMbeta2, CR3) acts as a functional receptor for platelet factor 4. *J Biol Chem* (2018) **293**:6869–6882. doi:10.1074/jbc.RA117.000515

30. Xiong Y, Cao C, Makarova A, Hyman B, Zhang L. Mac-1 promotes FcγRIIA-dependent cell spreading and migration on immune complexes. *Biochemistry* (2006) **45**:8721–8731. doi:10.1021/bi060529u

31. Saggu G, Okubo K, Chen Y, Vattepu R, Tsuboi N, Rosetti F, Cullere X, Washburn N, Tahir S, Rosado AM, et al. Cis interaction between sialylated FcγRIIA and the αI-domain of Mac-1 limits antibody-mediated neutrophil recruitment. *Nat Commun* (2018) **9**:5058. doi:10.1038/s41467-018-07506-1

32. Zhou M, Brown EJ. CR3 (Mac-1, alphaMbeta2 , CD11b / CD18 ) and FcgRIII Cooperate in Generation of a Neutrophil Respiratory Burst : Requirement for FcgRII and Tyrosine Phosphorylation. *J Cell Biol* (1994) **125**:1407–1416.

33. Chen J, Zhong MC, Guo H, Davidson D, Mishel S, Lu Y, Rhee I, Pérez-Quintero LA, Zhang S, Cruz-Munoz ME, et al. SLAMF7 is critical for phagocytosis of haematopoietic tumour cells via Mac-1 integrin. *Nature* (2017) **544**:493–497. doi:10.1038/nature22076

34. Rieu P, Ueda T, Haruta I, Sharma CP, Arnaout MA. The A-domain of β2 integrin CR3 (CD11b/CD18) is a receptor for the hookworm-derived neutrophil adhesion inhibitor NIF. *J Cell Biol* (1994) **127**:2081–2091. doi:10.1083/jcb.127.6.2081

35. Muchowski PJ, Chang ER, Soule HR, Moyle M, Zhang L, Plow EF. Functional interaction between the integrin antagonist neutrophil inhibitory factor and the I domain of CD11b/CD18. *J Biol Chem* (1994) **269**:26419–26423.

36. Ustinov VA, Plow EF. Delineation of the key amino acids involved in neutrophil inhibitory factor binding to the I-domain supports a mosaic model for the capacity of integrin αMβ2 to recognize multiple ligands. *J Biol Chem* (2002) **277**:18769–18776. doi:10.1074/jbc.M110242200

37. Zhang L, Plow EF. Identification and Reconstruction of the Binding Site within aMb2 for a Specific and High Affinity Ligand, NIF. *J Biol Chem* (1997) **272**:17558–17564. doi:10.1074/jbc.272.28.17558

38. Wright SD, Jong MTC. Adhesion-promoting receptors on human macrophages recognize escherichia coli by binding to lipopolysaccharide. *J Exp Med* (1986) **164**:1876–1888. doi:10.1084/jem.164.6.1876

39. Hmama Z, Mey A, Normier G, Binz H, Revillard JP. CD14 and CD11b mediate serum-independent binding to human monocytes of an acylpolygalactoside isolated from Klebsiella pneumoniae. *Infect Immun* (1994) **62**:1520–1527. doi:10.1128/iai.62.5.1520-1527.1994

40. Ross GD, Cain JA, Lachmann PJ. Membrane complement receptor type three ( CR3 ) has lectin-like properties analogous to bovine conglutinin as functions as a receptor for zymosan and rabbit erythrocytes as well as a receptor for iC3b. *J Immunol* (1985) **134**:3307–3315.

41. Větvička V, Thornton BP, Ross GD. Soluble β-glucan polysaccharide binding to the lectin site of neutrophil or natural killer cell complement receptor type 3 (CD11b/CD18) generates a primed state of the receptor capable of mediating cytotoxicity of iC3b-opsonized target cells. *J Clin Invest* (1996) **98**:50–61. doi:10.1172/JCI118777

42. Thornton BP, Vetvicka V, Pitman M, Goldman RC, Ross GD. Analysis of the Sugar Specificity and Molecular location of the beta-Glucan-Binding lectin Site of Complement Receptor Type 3 (CD11b/CD18). *J Immunol* (1996) **156**:1235–1246.

43. Bose N, Chan ASH, Guerrero F, Maristany CM, Qiu X, Walsh RM, Ertelt KE, Jonas AB, Gorden KB, Dudney CM, et al. Binding of soluble yeast β-glucan to human neutrophils and monocytes is complement-dependent. *Front Immunol* (2013) **4**:1–14. doi:10.3389/fimmu.2013.00230

44. van Bruggen R, Drewniak A, Jansen M, van Houdt M, Roos D, Chapel H, Verhoeven AJ, Kuijpers TW. Complement receptor 3, not Dectin-1, is the major receptor on human neutrophils for β-glucan-bearing particles. *Mol Immunol* (2009) **47**:575–581. doi:10.1016/j.molimm.2009.09.018

45. Zhou H, Liao J, Aloor J, Nie H, Wilson BC, Fessler MB, Gao H-MH-M, Hong J-SJ-S. CD11b/CD18 (Mac-1) Is a Novel Surface Receptor for Extracellular Double-Stranded RNA To Mediate Cellular Inflammatory Responses. *J Immunol* (2013) **190**:115–125. doi:10.4049/jimmunol.1202136

46. Zen K, Liu Y, Cairo D, Parkos CA. CD11b/CD18-Dependent Interactions of Neutrophils with Intestinal Epithelium Are Mediated by Fucosylated Proteoglycans. *J Immunol* (2002) **169**:5270–5278. doi:10.4049/jimmunol.169.9.5270

47. Dumont AL, Yoong P, Day CJ, Alonzo F, Mcdonald WH, Jennings MP, Torres VJ. Staphylococcus aureus LukAB cytotoxin kills human neutrophils by targeting the CD11b subunit of the integrin Mac-1. *Proc Natl Acad Sci* (2013) **110**:10794–10799. doi:10.1073/pnas.1305121110/-/DCSupplemental.www.pnas.org/cgi/doi/10.1073/pnas.1305121110

48. Trstenjak N, Milić D, Graewert MA, Rouha H, Svergun D, Djinović-Carugo K, Nagy E, Badarau A. Molecular mechanism of leukocidin GH–integrin CD11b/CD18 recognition and species specificity. *Proc Natl Acad Sci U S A* (2020) **117**:317–327. doi:10.1073/pnas.1913690116

49. Shewell LK, Day CJ, Jen FEC, Haselhorst T, Atack JM, Reijneveld JF, Everest-Dass A, James DBA, Boguslawski KM, Brouwer S, et al. All major cholesterol-dependent cytolysins use glycans as cellular receptors. *Sci Adv* (2020) **6**:1–13. doi:10.1126/sciadv.aaz4926

50. Cywes C, Godenir NL, Hoppe HC, Scholle RR, Steyn LM, Kirsch RE, Ehlers MRW. Nonopsonic binding of Mycobacterium tuberculosis to human complement receptor type 3 expressed in Chinese hamster ovary cells. *Infect Immun* (1996) **64**:5373–5383. doi:10.1128/iai.64.12.5373-5383.1996

51. Jennings MP, Jen FEC, Roddam LF, Apicella MA, Edwards JL. Neisseria gonorrhoeae pilin glycan contributes to CR3 activation during challenge of primary cervical epithelial cells. *Cell Microbiol* (2011) **13**:885–896. doi:10.1111/j.1462-5822.2011.01586.x

52. Edwards JL, Brown EJ, Uk-Nham S, Cannon JG, Blake MS, Apicella MA. A co-operative interaction between Neisseria gonorrhoeae and complement receptor 3 mediates infection of primary cervical epithelial cells. *Cell Microbiol* (2002) **4**:571–584. doi:10.1046/j.1462-5822.2002.t01-1-00215.x

53. Antal JM, Cunningham J V., Goodrum KJ. Opsonin-independent phagocytosis of group B streptococci: Role of complement receptor type three. *Infect Immun* (1992) **60**:1114–1121. doi:10.1128/iai.60.3.1114-1121.1992

54. Takeshita A, Murakami Y, Yamashita Y, Ishida M, Fujisawa S, Kitano S, Hanazawa S. Porphyromonas gingivalis fimbriae use β2 integrin (CD11/CD18) on mouse peritoneal macrophages as a cellular receptor, and the CD18 β chain plays a functional role in fimbrial signaling. *Infect Immun* (1998) **66**:4056–4060. doi:10.1128/iai.66.9.4056-4060.1998

55. Hajishengallis G. “Subversion of Innate Immunity by Periodontopathic Bacteria via Exploitation of Complement Receptor-3.,” in *Current Topics in Complement II. Advances in Experimental Medicine and Biology*, 203–219.

56. Relman D, Tuomanen E, Falkow S, Golenbock DT, Saukkonen K, Wright SD. Recognition of a bacterial adhesin by an integrin: Macrophage CR3 (αMβ2, CD11b CD18) binds filamentous hemagglutinin of Bordetella pertussis. *Cell* (1990) **61**:1375–1382. doi:10.1016/0092-8674(90)90701-F

57. Ishibashi Y, Claus S, Relman D. Bordetella pertussis Filamentous Hemagglutinin Interacts with a Leukocyte Signal Transduction Complex and Stimulates Bacterial Adherence to Monocyte CR3 (CD11b/CD18). *J Exp Med* (1994) **180**:1225–1233. doi:10.1016/B978-1-4557-4801-3.00232-0

58. Wong WSF, Simon DI, Rosoff PM, Rao NK, Chapman HA. Mechanisms of pertussis toxin‐induced myelomonocytic cell adhesion: role of Mac‐1 (CD11b/CD18) and urokinase receptor (CD87). *Immunology* (1996) **88**:90–97. doi:10.1046/j.1365-2567.1996.d01-646.x

59. McGuirk P, Mills KHG. Direct anti-inflammatory effect of a bacterial virulence factor: IL-10-dependent suppression of IL-12 production by filamentous hemagglutinin from Bordetella peptussis. *Eur J Immunol* (2000) **30**:415–422. doi:10.1002/1521-4141(200002)30:2<415::AID-IMMU415>3.0.CO;2-X

60. Guermonprez P, Khelef N, Blouin E, Rieu P, Ricciardi-Castagnoli P, Guiso N, Ladant D, Leclerc C. The Adenylate Cyclase Toxin of Bordetella pertussis Binds to Target Cells via the α M β 2 Integrin (Cd11b/Cd18) . *J Exp Med* (2001) **193**:1035–1044. doi:10.1084/jem.193.9.1035

61. Oliva C, Turnbough CL, Kearney JF. CD14-Mac-1 interactions in Bacillus anthracis spore internalization by macrophages. *Proc Natl Acad Sci* (2009) **106**:13957–13962. doi:10.1073/pnas.0902392106

62. Orrskog S, Rounioja S, Spadafina T, Gallotta M, Norman M, Hentrich K, Fälker S. Pilus Adhesin RrgA Interacts with Complement Receptor 3 , Thereby. *MBio* (2013) **4**:1–12. doi:10.1128/mBio.00535-12.Editor

63. Hoang K V., Rajaram MVS, Curry HM, Gavrilin MA, Wewers MD, Schlesinger LS. Complement receptor 3-mediated inhibition of inflammasome priming by Ras GTPase-activating protein during francisella tularensis phagocytosis by human mononuclear phagocytes. *Front Immunol* (2018) **9**:561. doi:10.3389/fimmu.2018.00561

64. Garcia RC, Murgia R, Cinco M. Complement receptor 3 binds the Borrelia burgdorferi outer surface proteins OspA and OspB in an iC3b-independent manner. *Infect Immun* (2005) **73**:6138–6142. doi:10.1128/IAI.73.9.6138-6142.2005

65. Blackwell JM. Receptors and recognition mechanisms of Leishmania species. *Trans R Soc Trop Med Hyg* (1985) **19**:606–612.

66. Russell DG. Complement receptor type 3 (CR3) binds to an Arg-Gly-Asp-containing region of the major surface glycoprotein, gp63, of Leishmania promastigotes. *J Exp Med* (1988) **168**:279–292. doi:10.1084/jem.168.1.279

67. Soteriadou KP, Remoundos MS, Katsikas MC, Tzinia AK, Tsikaris V, Sakarellos C, Tzartos SJ. The Ser-Arg-Tyr-Asp region of the major surface glycoprotein of Leishmania mimics the Arg-Gly-Asp-Ser cell attachment region of fibronectin. *J Biol Chem* (1992) **267**:13980–13985.

68. Tjomsland V, Ellegård R, Che K, Hinkula J, Lifson JD, Larsson M. Complement opsonization of HIV-1 enhances the uptake by dendritic cells and involves the endocytic lectin and integrin receptor families. *PLoS One* (2011) **6**:e23542. doi:10.1371/journal.pone.0023542

69. Ellegård R, Crisci E, Burgener A, Sjöwall C, Birse K, Westmacott G, Hinkula J, Lifson JD, Larsson M. Complement Opsonization of HIV-1 Results in Decreased Antiviral and Inflammatory Responses in Immature Dendritic Cells via CR3. *J Immunol* (2014) **193**:4590–4601. doi:10.4049/jimmunol.1401781

70. Crisci E, Ellegård R, Nyström S, Rondahl E, Serrander L, Bergström T, Sjöwall C, Eriksson K, Larsson M. Complement Opsonization Promotes Herpes Simplex Virus 2 Infection of Human Dendritic Cells. *J Virol* (2016) **90**:4939–4950. doi:10.1128/jvi.00224-16

71. Raftery MJ, Lalwani P, Krautkrӓmer E, Peters T, Scharffetter-Kochanek K, Krüger R, Hofmann J, Seeger K, Krüger DH, Schönrich G. β2 integrin mediates hantavirus-induced release of neutrophil extracellular traps. *J Exp Med* (2014) **211**:1485–1497. doi:10.1084/jem.20131092

72. Forsyth CB, Mathews HL. Lymphocytes Utilize CD11b/CD18 for Adhesion to Candida albicans. *Cell Immunol* (1996) **170**:91–100.

73. Hostetter MK. Adhesins and ligands involved in the interaction of Candida spp. with epithelial and endothelial surfaces. *Clin Microbiol Rev* (1994) **7**:29–42. doi:10.1128/CMR.7.1.29

74. Newman SL, Chaturvedi S, Klein BS. The WI-1 antigen of Blastomyces dermatitidis yeasts mediates binding to human macrophage CD11b/CD18 (CR3) and CD14. *J Immunol* (1995) **154**:753–761.

75. Bullock WE, Wright SD. Role or the adherence-promoting receptors, CR3, LFA-1, and p150,95, in binding of Histoplasma Capsulatum by human macrophages. *J Exp Med* (1987) **165**:195–210.

76. Davis GE. The Mac-1 and p150,95 beta2 Integrins Bind Denatured Proteins to Mediate Leukocyte Cell-Substrate Adhesion. *Exp Cell Res* (1992) **200**:242–252. doi:10.1016/0014-4827(92)90170-D

77. Brevig T, Holst B, Ademovic Z, Rozlosnik N, Røhrmann JH, Larsen NB, Hansen OC, Kingshott P. The recognition of adsorbed and denatured proteins of different topographies by β 2 integrins and effects on leukocyte adhesion and activation. *Biomaterials* (2005) **26**:3039–3053. doi:10.1016/j.biomaterials.2004.09.006

78. Godek ML, Michel R, Chamberlain LM, Castner DG, Grainger DW. Adsorbed serum albumin is permissive to macrophage attachment to perfluorocarbon polymer surfaces in culture. *J Biomed Mater Res - Part A* (2009) **88**:503–519. doi:10.1002/jbm.a.31886

79. Benimetskaya L, Loike JD, Khaled Z, Loike G, Silverstein SC, Cao L, El Kjoury J, Cai TQ, Stein CA. Mac-1 (CD1lb/CD18) is an oligodeoxynucleotide-binding protein. *Nat Med* (1997) **3**:414–420.

80. Rotshenker S. Microglia and macrophage activation and the regulation of complement-receptor-3 (CR3/MAC-1)-mediated myelin phagocytosis in injury and disease. *J Mol Neurosci* (2003) **21**:65–72. doi:10.1385/JMN:21:1:65

81. DeJong B, Smith M. A Role for Complement in Phagocytosis of Myelin. *Neurochem res* (1997) **22**:491–498. doi:10.1023/a:1027372129989

82. Stapulionis R, Pinto Oliveira CL, Gjelstrup MC, Pedersen JS, Hokland ME, Hoffmann SV, Poulsen K, Jacobsen C, Vorup-Jensen T. Structural Insight into the Function of Myelin Basic Protein as a Ligand for Integrin αMβ2. *J Immunol* (2008) **180**:3946–3956. doi:10.4049/jimmunol.180.6.3946

83. Goodwin JL, Kehrli ME, Uemura E. Integrin Mac-1 and β-amyloid in microglial release of nitric oxide. *Brain Res* (1997) **768**:279–286. doi:10.1016/S0006-8993(97)00653-7

84. Czirr E, Castello NA, Mosher KI, Castellano JM, Hinkson I V., Lucin KM, Baeza-Raja B, Ryu JK, Li L, Farina SN, et al. Microglial complement receptor 3 regulates brain Aβ levels through secreted proteolytic activity. *J Exp Med* (2017) **214**:1081–1092. doi:10.1084/jem.20162011

85. Fu H, Liu B, Frost JL, Hong S, Jin M, Ostaszewski B, Shankar GM, Costantino IM, Carroll MC, Mayadas TN, et al. Complement component C3 and complement receptor type 3 contribute to the phagocytosis and clearance of fibrillar Aβ by microglia. *Glia* (2012) **60**:993–1003. doi:10.1002/glia.22331

86. Choucair-Jaafar N, Laporte V, Levy R, Poindron P, Lombard Y, Gies JP. Complement receptor 3 (CD11b/CD18) is implicated in the elimination of β-amyloid peptides. *Fundam Clin Pharmacol* (2011) **25**:115–122. doi:10.1111/j.1472-8206.2010.00811.x

87. Zabel M, Schrag M, Crofton A, Tung S, Beaufond P, Van Ornam J, Dininni A, Vinters H V., Coppola G, Kirsch WM. A shift in microglial β-amyloid binding in Alzheimer’s disease is associated with cerebral amyloid angiopathy. *Brain Pathol* (2013) **23**:390–401. doi:10.1111/bpa.12005

88. Hou L, Bao X, Zang C, Yang H, Sun F, Che Y, Wu X, Li S, Zhang D, Wang Q. Integrin CD11b mediates α-synuclein-induced activation of NADPH oxidase through a Rho-dependent pathway. *Redox Biol* (2018) **14**:600–608. doi:10.1016/j.redox.2017.11.010

89. Hou L, Wang K, Zhang C, Sun F, Che Y, Zhao X, Zhang D, Li H, Wang Q. Complement receptor 3 mediates NADPH oxidase activation and dopaminergic neurodegeneration through a Src-Erk-dependent pathway. *Redox Biol* (2018) **14**:250–260. doi:10.1016/j.redox.2017.09.017

90. Zhang W, Dallas, Shannonhang D, Guo J-P, Pang H, Wilson B, Miller DS, Chen B, Zhang W, McGeer PL, Hong J-S, et al. Microglial PHOX and Mac-1 are Essential to the Enhanced Dopaminergic Neurodegeneration Elicited by A30P and A53T Mutant Alpha-Synuclein. *Glia* (2007) **55**:1178–1188. doi:10.1002/glia

91. Panina YA, Shilina E V. The Coexpression of CD157 / CD11b / CD18 in an Experimental Model of Parkinson ’ s Disease. (2015) **9**:312–316. doi:10.1134/S181971241504011X

92. Zhang C, Hou L, Yang J, Che Y, Sun F, Li H, Wang Q. 2,5-Hexanedione induces dopaminergic neurodegeneration through integrin αMβ2/NADPH oxidase axis-mediated microglial activation article. *Cell Death Dis* (2018) **9**: doi:10.1038/s41419-017-0091-7

93. Levesque S, Taetzsch T, Lull ME, Johnson JA, McGraw C, Block ML. The role of MAC1 in diesel exhaust particle-induced microglial activation and loss of dopaminergic neuron function. *J Neurochem* (2013) **125**:756–765. doi:10.1111/jnc.12231

94. Gao H-M, Zhou H, Zhang F, Wilson BC, Kam W, Hong J-S. HMGB1 Acts on Microglia Mac1 to Mediate Chronic Neuroinflammation That Drives Progressive Neurodegeneration. *J Neurosci* (2011) **31**:1081–1092. doi:10.1523/JNEUROSCI.3732-10.2011

95. Diamond MS, Staunton DE, Marlin SD, Springer TA. Binding of the integrin Mac-1 (CD11b/CD18) to the third immunoglobulin-like domain of ICAM-1 (CD54) and its regulation by glycosylation. *Cell* (1991) **65**:961–971. doi:10.1016/0092-8674(91)90548-D

96. McCleverty CJ, Liddington RC. Engineered allosteric mutants of the integrin alphaMbeta2 I domain: structural and functional studies. *Biochem J* (2003) **372**:121–127. doi:10.1042/bj20021273

97. Smith CW, Marlin SD, Rothlein R, Toman C, Anderson DC. Cooperative interactions of LFA-1 and Mac-1 with intracellular adhesion molecule-1 in facilitating adherence and transendothelial migration of human neutrophils in vitro. *J Clin Invest* (1989) **83**:2008–2017. doi:10.1172/JCI114111

98. Zhou L, Lee DHS, Plescia J, Lau CY, Altieri DC. Differential ligand binding specificities of recombinant CD11b/CD18 integrin I-domain. *J Biol Chem* (1994) **269**:17075–17079.

99. Li R, Xie J, Kantor C, Koistinen V, Altieri DC, Nortamo P, Gahmberg CG. A peptide derived from the intercellular adhesion molecule-2 regulates the avidity of the leukocyte integrins CD11b/CD18 and CD11c/CD18. *J Cell Biol* (1995) **129**:1143–1153. doi:10.1083/jcb.129.4.1143

100. Xie J, Li R, Kotovuori P, Vermot-Desroches C, Wijdenes J, Arnaout MA, Nortamo P, Gahmberg CG. Intercellular adhesion molecule-2 (CD102) binds to the leukocyte integrin CD11b/CD18 through the A domain. *J Immunol* (1995) **155**:3619–3628.

101. Hermand P, Huet M, Callebaut I, Gane P, Ihanus E, Gahmberg CG, Cartron JP, Bailly P. Binding sites of leukocyte β2 integrins (LFA-1, Mac-1) on the human ICAM-4/LW blood group protein. *J Biol Chem* (2000) **275**:26002–26010. doi:10.1074/jbc.M002823200

102. Santoso S, Sachs UJH, Kroll H, Linder M, Ruf A, Preissner KT, Chavakis T. The junctional adhesion molecule 3 (JAM-3) on human platelets is a counterreceptor for the leukocyte integrin Mac-1. *J Exp Med* (2002) **196**:679–691. doi:10.1084/jem.20020267

103. Zen K, Babbin BA, Liu Y, Whelan JB, Nusrat A, Parkos CA. JAM-C Is a Component of Desmosomes and a Ligand for CD11b/CD18-mediated Neutrophil Transepithelial Migration. *Mol Biol Cell* (2004) **15**:3926–3937. doi:10.1091/mbc.E04

104. Heinzmann D, Noethel M, Ungern-Sternberg S V., Mitroulis I, Gawaz M, Chavakis T, May AE, Seizer P. CD147 is a novel interaction partner of integrin αMβ2 mediating leukocyte and platelet adhesion. *Biomolecules* (2020) **10**:7–12. doi:10.3390/biom10040541

105. Chavakis T, Bierhaus A, Al-Fakhri N, Schneider D, Witte S, Linn T, Nagashima M, Morser J, Arnold B, Preissner KT, et al. The Pattern Recognition Receptor (RAGE) Is a Counterreceptor for Leukocyte Integrins: A Novel Pathway for Inflammatory Cell Recruitment. *J Exp Med* (2003) **198**:15007–1515. doi:10.1084/jem.20030800

106. Wetzel A, Chavakis T, Preissner KT, Sticherling M, Haustein U-F, Anderegg U, Saalbach A. Human Thy-1 (CD90) on Activated Endothelial Cells Is a Counterreceptor for the Leukocyte Integrin Mac-1 (CD11b/CD18). *J Immunol* (2004) **172**:3850–3859. doi:10.4049/jimmunol.172.6.3850

107. Wetzel A, Wetzig T, Haustein UF, Sticherling M, Anderegg U, Simon JC, Saalbach A. Increased neutrophil adherence in psoriasis: Role of the human endothelial cell receptor Thy-1 (CD90). *J Invest Dermatol* (2006) **126**:441–452. doi:10.1038/sj.jid.5700072

108. Podolnikova NP, Hlavackova M, Wu Y, Yakubenko VP, Faust J, Balabiyev A, Wang X, Ugarova TP. Interaction between the integrin Mac-1 and signal regulatory protein α (SIRPα) mediates fusion in heterologous cells. *J Biol Chem* (2019) **294**:7833–7849. doi:10.1074/jbc.RA118.006314

109. Wolf D, Hohmann JD, Wiedemann A, Bledzka K, Blankenbach H, Marchini T, Gutte K, Zeschky K, Bassler N, Hoppe N, et al. Binding of CD40L to Mac-1’s i-domain involves the EQLKKSKTL motif and mediates leukocyte recruitment and atherosclerosis-but does not affect immunity and thrombosis in mice. *Circ Res* (2011) **109**:1269–1279. doi:10.1161/CIRCRESAHA.111.247684

110. Cai TQ. Human leukocyte elastase is an endogenous ligand for the integrin CR3 (CD11b/CD18, Mac-1, alpha M beta 2) and modulates polymorphonuclear leukocyte adhesion. *J Exp Med* (1996) **184**:1213–1223. doi:10.1084/jem.184.4.1213

111. Johansson MW, Patarroyo M, Öberg F, Siegbahn A, Nilsson K. Myeloperoxidase mediates cell adhesion via the aMb2 integrin (Mac-1, CD11b/CD18). *J Cell Sci* (1997) **110**:1133–1139.

112. Shen D, Podolnikova NP, Yakubenko VP, Ardell CL, Balabiyev A, Ugarova TP, Wang X. Pleiotrophin, a multifunctional cytokine and growth factor, induces leukocyte responses through the integrin Mac-1. *J Biol Chem* (2017) **292**:18848–18861. doi:10.1074/jbc.M116.773713

113. Podolnikova NP, Brothwell JA, Ugarova TP. The Opioid Peptide Dynorphin A Induces Leukocyte Responses via Integrin Mac-1 (αMβ2 , CD11b/CD18). *Mol Pain* (2015) **11**:33. doi:10.1186/s12990-015-0027-0

114. Stefanidakis M, Björklund M, Ihanus E, Gahmberg CG, Koivunen E. Identification of a Negatively Charged Peptide Motif within the Catalytic Domain of Progelatinases That Mediates Binding to Leukocyte β2 Integrins. *J Biol Chem* (2003) **278**:34674–34684. doi:10.1074/jbc.M302288200

115. Stefanidakis M, Ruohtula T, Borregaard N, Gahmberg CG, Koivunen E. Intracellular and Cell Surface Localization of a Complex between α M β 2 Integrin and Promatrix Metalloproteinase-9 Progelatinase in Neutrophils . *J Immunol* (2004) **172**:7060–7068. doi:10.4049/jimmunol.172.11.7060

116. Van Gisbergen KPJM, Sanchez-Hernandez M, Geijtenbeek TBH, Van Kooyk Y. Neutrophils mediate immune modulation of dendritic cells through glycosylation-dependent interactions between Mac-1 and DC-SIGN. *J Exp Med* (2005) **201**:1281–1292. doi:10.1084/jem.20041276

117. Nathan C, Srimal S, Farber C, Sanchez E, Kabbash L, Asch A, Gailit J, Wright SD. Cytokine-induced respiratory burst of human neutrophils: Dependence on extracellular matrix proteins and CD11/CD18 integrins. *J Cell Biol* (1989) **109**:1341–1349. doi:10.1083/jcb.109.3.1341

118. Kanse SM, Matz RL, Preissner KT, Peter K. Promotion of Leukocyte Adhesion by a Novel Interaction Between Vitronectin and the beta2 Integrin Mac-1 (alphaMbeta2, CD11b/CD18). *Atherioscler Thromb Vasc Biol* (2004) **24**:2251–2256. doi:10.1161/01.ATV.0000146529.68729.8b

119. Gustafson EJ, Lukasiewicz H, Wachtfogel YT, Norton KJ, Schmaier AH, Niewiarowski S, Colman RW. High molecular weight kininogen inhibits fibrinogen binding to cytoadhesins of neutrophils and platelets. *J Cell Biol* (1989) **109**:377–387. doi:10.1083/jcb.109.1.377

120. Morgan J, Saleem M, Ng R, Armstrong C, Wong SS, Caulton SG, Fickling A, Williams HELL, Munday AD, López JA, et al. Structural basis of the leukocyte integrin Mac-1 I-domain interactions with the platelet glycoprotein Ib. *Blood Adv* (2019) **3**:1450–1459. doi:10.1182/bloodadvances.2018027011

121. Ehlers R, Ustinov V, Chen Z, Zhang X, Rao R, Luscinskas FW, Lopez J, Plow E, Simon DI. Targeting platelet-leukocyte interactions: Identification of the integrin Mac-1 binding site for the platelet counter receptor glycoprotein Ibα. *J Exp Med* (2003) **198**:1077–1088. doi:10.1084/jem.20022181

122. Wang Z, Thinn AMM, Zhu J. A pivotal role for a conserved bulky residue at the α1-helix of the αI integrin domain in ligand binding. *J Biol Chem* (2017) **292**:20756–20768. doi:10.1074/jbc.M117.790519

123. Flick MJ, Du XL, Witte DP, Jiroušková M, Soloviev DA, Busuttil SJ, Plow EF, Degen JL. Leukocyte engagement of fibrin(ogen) via the integrin receptor αMβ2/Mac-1 is critical for host inflammatory response in vivo. *J Clin Invest* (2004) **113**:1596–1606. doi:10.1172/JCI20741

124. Perez RL, Ritzenthaler JD, Roman J. Transcriptional Regulation of the Interleukin-1β Promoter via Fibrinogen Engagement of the CD18 Integrin Receptor. *J Respir Cell Mol Biol* (1999) **20**:1059–1066.

125. Fan S-T, Edgington TS. lntegrin Regulation of Leukocyte Inflammatory Functions CD11b/CD18 Enhancement of the Tumor Necrosis Factor-alpha Responses of Monocytes. *J Immunol* (1993) **150**:2972–2980.

126. Nasimuzzaman M, Arumugam PI, Mullins ES, James JM, VandenHeuvel K, Narciso MG, Shaw MA, McGraw S, Aronow BJ, Malik P. Elimination of the fibrinogen integrin α M β 2 -binding motif improves renal pathology in mice with sickle cell anemia . *Blood Adv* (2019) **3**:1519–1532. doi:10.1182/bloodadvances.2019032342

127. Vidal B, Ardite E, Suelves M, Ruiz-Bonilla V, Janué A, Flick MJ, Degen JL, Serrano AL, Muñoz-Cánoves P. Amelioration of Duchenne muscular dystrophy in mdx mice by elimination of matrix-associated fibrin-driven inflammation coupled to the αMβ2 leukocyte integrin receptor. *Hum Mol Genet* (2012) **21**:1989–2004. doi:10.1093/hmg/dds012

128. Yakubenko VP, Solovjov DA, Zhang L, Yee VC, Plow EF, Ugarova TP. Identification of the Binding Site for Fibrinogen Recognition Peptide γ383-395 within the αMI-Domain of Integrin αMβ2. *J Biol Chem* (2001) **276**:13995–14003. doi:10.1074/jbc.M010174200

129. Lishko VK, Novokhatny V V., Yakubenko VP, Skomorovska-Prokvolit H V., Ugarova TP. Characterization of plasminogen as an adhesive ligand for integrins αMβ2 (Mac-1) and α5β1 (VLA-5). *Blood* (2004) **104**:719–726. doi:10.1182/blood-2003-09-3016

130. Wright SD, Weitz JI, Huang AJ, Levin SM, Silverstein SC, Loike JD. Complement receptor type three (CD11b/CD18) of human polymorphonuclear leukocytes recognizes fibrinogen. *Proc Natl Acad Sci* (1988) **85**:7734–7738. doi:10.1073/pnas.85.20.7734

131. Altieri DC, Bader R, Mannucci PM, Edgington TS. Oligospecificity of the cellular adhesion receptor Mac-1 encompasses an inducible recognition specificity for fibrinogen. *J Cell Biol* (1988) **107**:1893–1900. doi:10.1083/jcb.107.5.1893

132. Altieri DC, Agbanyo FR, Plescia J, Ginsberg MH, Edgington TS, Plow EF. A unique recognition site mediates the interaction of fibrinogen with the leukocyte integrin Mac-1 (CD11b/CD18). *J Biol Chem* (1990) **265**:12119–12122.

133. Altieri DC, Plescia J, Plow EF. The Structural Motif Glycine 190-Valine 202 of the Fibrinogen y Chain Interacts with CD11b/CD18 Integrin (aMb2, Mac-1) and Promotes Leukocyte Adhesion. *J Biol Chem* (1993) **268**:1847–1853.

134. Ugarova TP, Solovjov DA, Zhang L, Loukinov DI, Yee VC, Medved L V, Plow EF. Identification of a Novel Recognition Sequence for Integrin aMb2 within the y-chain of Fibrinogen. *J Biol Chem* (1998) **273**:22519–22527.

135. Ugarova TP, Yakubenko V. Recognition of Fibrinogen by Leukocyte Integrins. *Ann N Y Acad Sci* (2001) **936**:368–385. doi:10.1111/j.1749-6632.2001.tb03523.x

136. Lishko VK, Kudryk B, Yakubenko VP, Yee VC, Ugarova TP. Regulated unmasking of the cryptic binding site for integrin αMβ2 in the γC-domain of fibrinogen. *Biochemistry* (2002) **41**:12942–12951. doi:10.1021/bi026324c

137. Ugarova TP, Lishko VK, Podolnikova NP, Okumura N, Merkulov SM, Yakubenko VP, Yee VC, Lord ST, Haas TA. Sequence γ377-395(P2), but not γ190-202(P1), is the binding site for the αMI-domain of integrin αMβ2 in the αC-domain of fibrinogen. *Biochemistry* (2003) **42**:9365–9373. doi:10.1021/bi034057k

138. Houimel M, Mazzucchelli L. Random phage-epitope library based identification of a peptide antagonist of Mac-1 β2 integrin ligand binding. *Matrix Biol* (2012) **31**:66–77. doi:10.1016/j.matbio.2011.10.003

139. Lishko VK, Podolnikova NP, Yakubenko VP, Yakovlev S, Medved L, Yadav SP, Ugarova TP. Multiple binding sites in fibrinogen for integrin αMβ 2 (Mac-1). *J Biol Chem* (2004) **279**:44897–44906. doi:10.1074/jbc.M408012200

140. Yakovlev S, Zhang L, Ugarova T, Medved L. Interaction of fibrin(ogen) with leukocyte receptor α Mβ2 (Mac-1): Further characterization and identification of a novel binding region within the central domain of the fibrinogen γ-module. *Biochemistry* (2005) **44**:617–626. doi:10.1021/bi048266w

141. Lishko VK, Yakubenko VP, Hertzberg KM, Grieninger G, Ugarova TP. The alternatively spliced alpha(E)C domain of human fibrinogen-420 is a novel ligand for leukocyte integrins alpha(M)beta(2) and alpha(X)beta(2). *Blood* (2001) **98**:2448–55.

142. Lishko VK, Yakubenko VP, Ugarova TP. The interplay between integrins αMβ2 and α5β1 during cell migration to fibronectin. *Exp Cell Res* (2003) **283**:116–126. doi:10.1016/S0014-4827(02)00024-1

143. Thompson HL, Matsushima K. Human polymorphonuclear leucocytes stimulated by tumour necrosis factor-alpha show increased adherence to extracellular matrix proteins which is mediated via the CD11b/18 complex. *Clin Exp Immunol* (1992) **90**:280–285. doi:10.1111/j.1365-2249.1992.tb07943.x

144. Walzog B, Schuppan D, Heimpel C, Hafezi-Moghadam A, Gaehtgens P, Ley K. The Leukocyte Integrin Mac-1 (CD11b/CD18) contributes to binding of human granulocytes to collagen. *Exp Cell Res* (1995) **218**:28–38.

145. Lahti M, Heino J, Käpylä J. Leukocyte integrins αLβ2, αMβ2 and αXβ2 as collagen receptors - Receptor activation and recognition of GFOGER motif. *Int J Biochem Cell Biol* (2013) **45**:1204–1211. doi:10.1016/j.biocel.2013.03.016

146. Bohnsack JF, Akiyama SK, Damsky CH, Knapej WA, Zimmermansii GA. Human neutrophil adherence to laminin in vitro: Evidence for a distinct neutrophil integrin receptor for laminin. *J Exp Med* (1990) **171**:1221–1237. doi:10.1084/jem.171.4.1221

147. Lee S, Bowrin K, Hamad AR, Chakravarti S. Extracellular matrix lumican deposited on the surface of neutrophils promotes migration by binding to β2 integrin. *J Biol Chem* (2009) **284**:23662–23669. doi:10.1074/jbc.M109.026229

148. He YW, Li H, Zhang J, Hsu CL, Lin E, Zhang N, Guo J, Forbush KA, Bevan MJ. The extracellular matrix protein mindin is a pattern-recognition molecule for microbial pathogens. *Nat Immunol* (2004) **5**:88–97. doi:10.1038/ni1021

149. Liu Y sheng, Wang L fen, Cheng XS, Huo YN, Ouyang XM, Liang LY, Lin Y, Wu JF, Ren JL, Guleng B. The pattern‐recognition molecule mindin binds integrin Mac‐1 to promote macrophage phagocytosis via Syk activation and NF‐κB p65 translocation. *J Cell Mol Med* (2019) **23**:3402–3416. doi:10.1111/jcmm.14236

150. Schober JM, Lau LF, Ugarova TP, Lam SCT. Identification of a novel integrin αMβ2 binding site in CCN1 (CYR61), a matricellular protein expressed in healing wounds and atherosclerotic lesions. *J Biol Chem* (2003) **278**:25808–25815. doi:10.1074/jbc.M301534200

151. Schober JM, Chen N, Grzeszkiewicz TM, Jovanovic I, Emeson EE, Ugarova TP, Ye RD, Lau LF, C-T Lam S. Identification of integrin alphaMbeta2 as an adhesion receptor on peripheral blood monocytes for Cyr61 (CCN1) and connective tissue growth factor (CCN2): immediate-early gene products expressed in atherosclerotic lesions. *Blood* (2002) **99**:4457–4465.

152. Bai T, Chen C-C, Lau LF. Matricellular Protein CCN1 Activates a Proinflammatory Genetic Program in Murine Macrophages. *J Immunol* (2010) **184**:3223–3232. doi:10.4049/jimmunol.0902792

153. Yakubenko VP, Cui K, Ardell CL, Brown KE, West XZ, Gao D, Stefl S, Salomon RG, Podrez EA, Byzova T V., et al. Oxidative modifications of extracellular matrix promote the second wave of inflammation via b2 integrins. *Blood* (2018) **132**:78–88. doi:10.1182/blood-2017-10-810176

154. Chavakis T, Athanasopoulos A, Rhee JS, Orlova V, Schmidt-Wöll T, Bierhaus A, May AE, Celik I, Nawroth PP, Preissner KT. Angiostatin is a novel anti-inflammatory factor by inhibiting leukocyte recruitment. *Blood* (2005) **105**:1036–1043. doi:10.1182/blood-2004-01-0166

155. Sotiriou SN, Orlova V V., Al‐Fakhri N, Ihanus E, Economopoulou M, Isermann B, Bdeir K, Nawroth PP, Preissner KT, Gahmberg CG, et al. Lipoprotein(a) in atherosclerotic plaques recruits inflammatory cells through interaction with Mac‐1 integrin. *FASEB J* (2006) **20**:559–561. doi:10.1096/fj.05-4857fje

156. Simon DI, Wei Y, Zhang L, Rao NK, Xu H, Chen Z, Liu Q, Rosenberg S, Chapman HA. Identification of a urokinase receptor-integrin interaction site. Promiscuous regulator of integrin function. *J Biol Chem* (2000) **275**:10228–10234. doi:10.1074/jbc.275.14.10228

157. Zhang H, Colman RW, Sheng N. Regulation of CD11b/CD18 (Mac-1) adhesion to fibrinogen by urokinase receptor (uPAR). *Inflamm Res* (2003) **52**:86–93. doi:10.1007/s000110300006

158. May AE, Kanse SM, Lund LR, Gisler RH, Imhof BA, Preissner KT. Urokinase Receptor (CD87) Regulates Leukocyte Recruitment via β 2 Integrins In Vivo. *J Exp Med* (1998) **188**:1029–1037. doi:10.1084/jem.188.6.1029

159. Xue W, Kindzelskii AL, Todd RF, Petty HR. Physical association of complement receptor type 3 and urokinase-type plasminogen activator receptor in neutrophil membranes. *J Immunol* (1994) **152**:4630–40.

160. Lin L, Wu C, Hu K. Tissue plasminogen activator activates NF-κB through a pathway involving annexin A2/CD11b and integrin-linked kinase. *J Am Soc Nephrol* (2012) **23**:1329–1338. doi:10.1681/ASN.2011111123

161. Cao C, Lawrence DA, Li Y, Von Arnim CAF, Herz J, Su EJ, Makarova A, Hyman BT, Strickland DK, Zhang L. Endocytic receptor LRP together with tPA and PAI-1 coordinates Mac-1-dependent macrophage migration. *EMBO J* (2006) **25**:1860–1870. doi:10.1038/sj.emboj.7601082

162. Jerke U, Rolle S, Dittmar G, Bayat B, Santoso S, Sporbert A, Luft F, Kettritz R. Complement receptor Mac-1 is an adaptor for NB1 (CD177)-mediated PR3-ANCA neutrophil activation. *J Biol Chem* (2011) **286**:7070–7081. doi:10.1074/jbc.M110.171256

163. Ranganathan S, Cao C, Catania J, Migliorini M, Zhang L, Strickland DK. Molecular basis for the interaction of low density lipoprotein receptor-related protein 1 (LRP1) with integrin αMβ2: Identification of binding sites within αMβ2 for LRP1. *J Biol Chem* (2011) **286**:30535–30541. doi:10.1074/jbc.M111.265413

164. Spijkers PPEM, da Costa Martins P, Westein E, Gahmberg CG, Zwaginga JJ, Lenting PJ. LDL-receptor – related protein regulates beta2-integrin – mediated leukocyte adhesion. *Blood* (2005) **105**:170–177. doi:10.1182/blood-2004-02-0498.Supported

165. Simon DI, Chen Z, Xu H, Li CQ, Dong JF, McIntire L V., Ballantyne CM, Zhang L, Furman MI, Berndt MC, et al. Platelet Glycoprotein Ibalpha is a counterreceptor for the leukocyte integrin Mac-1 (CD11b/CD18). *J Exp Med* (2000) **192**:193–204. doi:10.1084/JEM.192.2.193

166. Wang Y, Gao H, Shi C, Erhardt PW, Pavlovsky A, Soloviev DA, Bledzka K, Ustinov V, Zhu L, Qin J, et al. Leukocyte integrin Mac-1 regulates thrombosis via interaction with platelet GPIbα. *Nat Commun* (2017) **8**:15559. doi:10.1038/ncomms15559

167. Chavakis T, Santoso S, Clemetson KJ, Sachs UJH, Isordia-Salas I, Pixley RA, Nawroth PP, Colman RW, Preissner KT. High Molecular Weight Kininogen Regulates Platelet-Leukocyte Interactions by Bridging Mac-1 and Glycoprotein Ib. *J Biol Chem* (2003) **278**:45375–45381. doi:10.1074/jbc.M304344200

168. Hidalgo A, Peired AJ, Weiss LA, Katayama Y, Frenette PS. The integrin αMβ2 anchors hematopoietic progenitors in the bone marrow during enforced mobilization. *Blood* (2004) **104**:993–1001. doi:10.1182/blood-2003-10-3702

169. Zen K, Liu DQ, Li LM, Chen CXJ, Guo YL, Ha B, Chen X, Zhang CY, Liu Y. The heparan sulfate proteoglycan form of epithelial CD44v3 serves as a CD11b/CD18 counter-receptor during polymorphonuclear leukocyte transepithelial migration. *J Biol Chem* (2009) **284**:3768–3776. doi:10.1074/jbc.M807805200

170. Diamond MS, Alon R, Parkos CA, Quinn MT, Springer TA. Heparin is an adhesive ligand for the leukocyte integrin Mac-1 (CD11b/CD18). *J Cell Biol* (1995) **130**:1473–1482. doi:10.1083/jcb.130.6.1473

171. Peter K, Schwarz M, Conradt C, Nordt T, Moser M, Kübier W, Bode C. Heparin inhibits ligand binding to the leukocyte integrin Mac-1 (CD11b/CD18). *Circulation* (1999) **100**:1533–1539. doi:10.1161/01.CIR.100.14.1533

172. Altieri DC, Edgington TS. The Saturable High Affinity Association of Factor X to ADP-stimulated Monocytes Defines a Novel Function of the Mac-1 Receptor. *J Biol Chem* (1988) **263**:7007–7015.

173. Altieri DC, Etingin OR, Fair DS, Brunck TK, Geltosky JE, Hajjar DP, Edgington TS. Structurally homologous ligand binding of integrin Mac-1 and viral glycoprotein C receptors. *Science (80- )* (1991) **254**:1200–1202. doi:10.1126/science.1957171

174. Plescia J, Altieri DC. Activation of Mac-1 (CD11b/CD18)-bound factor X by released cathepsin G Defines an Alternative Pathway of Leucocyte Initiation of Coagulation. *Cell* (1996) **879**:873–879.

175. Sheng N, Fairbanks MB, Heinrikson RL, Canziani G, Chaiken IM, Mosser DM, Zhang H, Colman RW. Cleaved high molecular weight kininogen binds directly to the integrin CD11b/CD18 (Mac-1) and blocks adhesion to fibrinogen and ICAM-1. *Blood* (2000) **95**:3788–3795. doi:10.1182/blood.v95.12.3788.012k47_3788_3795

176. Wachtfogel YT, DeLa Cadena RA, Kunapuli SP, Rick L, Miller M, Schultze RL, Altieri DC, Edgington TS, Colman RW. High molecular weight kininogen binds to Mac-1 on neutrophils by its heavy chain (domain 3) and its light chain (domain 5). *J Biol Chem* (1994) **269**:19307–19312.

177. Khan MM, Bradford HN, Isordia-Salas I, Liu Y, Wu Y, Espinola RG, Ghebrehiwet B, Colman RW. High-molecular-weight kininogen fragments stimulate the secretion of cytokines and chemokines through uPAR, Mac-1, and gC1qR in monocytes. *Arterioscler Thromb Vasc Biol* (2006) **26**:2260–2266. doi:10.1161/01.ATV.0000240290.70852.c0

178. Gustafson EJ, Schmaier AH, Wachtfogel YT, Kaufman N, Kucich U, Colman RW. Human neutrophils contain and bind high molecular weight kininogen. *J Clin Invest* (1989) **84**:28–35. doi:10.1172/JCI114151

179. Chavakis T, Kanse SM, Pixley RA, May AE, Isordia-Salas I, Colman RW, Preissner KT. Regulation of leukocyte recruitment by polypeptides derived from high molecular weight kininogen. *FASEB J* (2001) **15**:2365–2376. doi:10.1096/fj.01-0201com

180. Kawamoto E, Okamoto T, Takagi Y, Honda G, Suzuki K, Imai H, Shimaoka M. LFA-1 and Mac-1 integrins bind to the serine/threonine-rich domain of thrombomodulin. *Biochem Biophys Res Commun* (2016) **473**:1005–1012. doi:10.1016/j.bbrc.2016.04.007

181. Watanabe-Kusunoki K, Nakazawa D, Kusunoki Y, Kudo T, Hattanda F, Nishio S, Masuda S, Tomaru U, Kondo T, Atsumi T, et al. Recombinant thrombomodulin ameliorates autoimmune vasculitis via immune response regulation and tissue injury protection. *J Autoimmun* (2020) **108**:102390. doi:10.1016/j.jaut.2019.102390

182. Fink K, Busch HJ, Bourgeois N, Schwarz M, Wolf D, Zirlik A, Peter K, Bode C, von zur Muhlen C. Mac-1 Directly Binds to the Endothelial Protein C-Receptor: A Link between the Protein C Anticoagulant Pathway and Inflammation? *PLoS One* (2013) **8**:1–5. doi:10.1371/journal.pone.0053103

183. Hahm E, Li J, Kim K, Huh S, Rogelj S, Cho J. Extracellular protein disulfide isomerase regulates ligand-binding activity of aMb2 integrin and neutrophil recruitment during vascular inflammation. *Blood* (2013) **121**:3789–3800. doi:10.1182/blood-2012

184. Zen K, Utech M, Liu Y, Soto I, Nusrat A, Parkos CA. Association of BAP31 with CD11b/CD18. Potential role in intracellular trafficking of CD11b/CD18 in neutrophils. *J Biol Chem* (2004) **279**:44924–44930. doi:10.1074/jbc.M402115200

185. Ding C, Ma Y, Chen X, Liu M, Cai Y, Hu X, Xiang D, Nath S, Zhang HG, Ye H, et al. Integrin CD11b negatively regulates BCR signalling to maintain autoreactive B cell tolerance. *Nat Commun* (2013) **4**:2813. doi:10.1038/ncomms3813

186. Lecoanet-Henchoz S, Gauchat JF, Aubry JP, Graber P, Life P, Paul-Eugene N, Ferrua B, Corbi AL, Dugas B, Plater-Zyberk C, et al. CD23 Regulates monocyte activation through a novel interaction with the adhesion molecules CD11b-CD18 and CD11c-CD18. *Immunity* (1995) **3**:119–125. doi:10.1016/1074-7613(95)90164-7

187. Cao C, Zhao J, Doughty EK, Migliorini M, Strickland DK, Kann MG, Zhang L. Mac-1 regulates IL-13 activity in macrophages by directly interacting with IL-13Rα1. *J Biol Chem* (2015) **290**:21642–21651. doi:10.1074/jbc.M115.645796

188. Schuler P, Assefa D, Ylänne J, Basler N, Olschewski M, Ahrens I, Nordt T, Bode C, Peter K. Adhesion of monocytes to medical steel as used for vascular stents is mediated by the integrin receptor Mac-1 (CD11b/CD18; αM β2) and can be inhibited by semiconductor coating. *Cell Commun Adhes* (2003) **10**:17–26. doi:10.1080/15419060302065

189. Biothera. Efficacy/Safety of Imprime PGG With Cetuximab & Paclitaxel/Carboplatin Therapy in Pts With Untreated Advanced Non-Small Cell Lung Cancer. *NCT00874848* (2016)

190. Biothera. Study of Imprime PGG and Pembrolizumab in Advanced Melanoma and Triple Negative Breast Cancer. *NCT02981303* (2019)

191. Biothera. Efficacy and Safety Study of Imprime PGG With Cetuximab in Subjects With Stage IV KRAS-Mutated Colorectal Cancer. *NCT00912327* (2012)

192. Biothera. Pembrolizumab + Imprime PGG for Metastatic Non-small Cell Lung Cancer After Progression on First-Line Therapy: Big Ten Cancer Research Consortium BTCRC-LUN15-017. *NCT03003468* (2019)

193. Yan TT, Li Q, Zhou HT, Zhao YT, Yu SQ, Xu GL, Yin ZM, Li ZJ, Zhao ZH. Gu-4 suppresses affinity and avidity modulation of CD11b and improves the outcome of mice with endotoxemia and sepsis. *PLoS One* (2012) **7**:e30110. doi:10.1371/journal.pone.0030110

194. Trentini A, Murganti F, Rosta V, Cervellati C, Manfrinato MC, Spadaro S, Dallocchio F, Volta CA, Bellini T. Hydroxyethyl starch 130/0.4 binds to neutrophils impairing their chemotaxis through a Mac-1 dependent interaction. *Int J Mol Sci* (2019) **20**:817. doi:10.3390/ijms20040817

195. Schwarz M, Nordt T, Bode C, Peter K. The GP IIb/IIIa inhibitor abciximab (c7E3) inhibits the binding of various ligands to the leukocyte integrin Mac-1 (CD11b/CD18, αMβ2). *Thromb Res* (2002) **107**:121–128. doi:10.1016/S0049-3848(02)00207-4

196. Endemann G, Feng Y, Bryant CM, Hamilton GS, Perumattam J, Mewshaw RE, Liu DY. Novel anti-inflammatory compounds prevent CD11b/CD18, αMβ2 (Mac-1)-dependent neutrophil adhesion without blocking activation-induced changes in mac-1. *J Pharmacol Exp Ther* (1996) **276**:5–12.

197. Hamilton GS, Mewshaw RE, Bryant CM, Feng Y, Endemann G, Madden KS, Janczak JE, Perumattam J, Stanton LW, Yang X, et al. Fluorenylalkanoic and Benzoic Acids as Novel Inhibitors of Cell Adhesion Processes in Leukocytes. *J Med Chem* (1995) **38**:1650–1656. doi:10.1021/jm00010a009

198. Bansal VS, Vaidya S, Somers EP, Kanuga M, Shevell D, Weikel R, Detmers PA. Small molecule antagonists of complement receptor type 3 block adhesion and adhesion-dependent oxidative burst in human polymorphonuclear leukocytes. *J Pharmacol Exp Ther* (2003) **304**:1016–1024. doi:10.1124/jpet.102.045286

199. Faridi MH, Maiguel D, Brown BT, Suyama E, Barth CJ, Hedrick M, Vasile S, Sergienko E, Schürer S, Gupta V. High-throughput screening based identification of small molecule antagonists of integrin CD11b/CD18 ligand binding. *Biochem Biophys Res Commun* (2010) **394**:194–199. doi:10.1016/j.bbrc.2010.02.151

200. Shimaoka M, Salas A, Yang W, Weitz-Schmidt G, Springer TA. Small Molecule Integrin Antagonists that Bind to the beta2 Subunit I-like Domain and Activate Signals in One Direction and Block Them in the Other. *Immunity* (2003) **19**:391–402. doi:10.1016/s1074-7613(03)00238-3

201. Jensen MR, Bajic G, Zhang X, Laustsen AK, Koldsø H, Skeby KK, Schiøtt B, Andersen GR, Vorup-Jensen T. Structural Basis for Simvastatin Competitive Antagonism of Complement Receptor 3. *J Biol Chem* (2016) **291**:16963–76. doi:10.1074/jbc.M116.732222

202. Koivunen E, Ranta TM, Annila A, Taube S, Uppala A, Jokinen M, Van Willigen G, Ihanus E, Gahmberg CG. Inhibition of β2 integrin-mediated leukocyte cell adhesion by leucine-leucine-glycine motif-containing peptides. *J Cell Biol* (2001) **153**:905–915. doi:10.1083/jcb.153.5.905

203. Feng Y, Chung D, Garrard L, McEnroe G, Lim D, Scardina J, McFadden K, Guzzetta A, Lam A, Abraham J, et al. Peptides derived from the complementarity-determining regions of anti- Mac-1 antibodies block intercellular adhesion molecule-1 interaction with Mac-1. *J Biol Chem* (1998) **273**:5625–5630. doi:10.1074/jbc.273.10.5625

204. Björklund M, Aitio O, Stefanidakis M, Suojanen J, Salo T, Sorsa T, Koivunen E. Stabilization of the activated αMβ2 integrin by a small molecule inhibits leukocyte migration and recruitment. *Biochemistry* (2006) **45**:2862–2871. doi:10.1021/bi052238b

205. Faridi MH, Maiguel D, Barth CJ, Stoub D, Day R, Schürer S, Gupta V. Identification of novel agonists of the integrin CD11b/CD18. *Bioorganic Med Chem Lett* (2009) **19**:6902–6906. doi:10.1016/j.bmcl.2009.10.077

206. Faridi MH, Altintas MM, Gomez C, Duque JC, Vazquez-Padron RI, Gupta V. Small molecule agonists of integrin CD11b/CD18 do not induce global conformational changes and are significantly better than activating antibodies in reducing vascular injury. *Biochim Biophys Acta* (2013) **1830**:3696–3710. doi:10.1016/j.bbagen.2013.02.018

207. Maiguel D, Faridi MH, Wei C, Kuwano Y, Balla KM, Hernandez D, Barth CJ, Lugo G, Donnelly M, Nayer A, et al. Small molecule-mediated activation of the integrin CD11b/CD18 reduces inflammatory disease. *Sci Signal* (2011) **4**:1–15. doi:10.1126/scisignal.2001811

208. Roberts AL, Fürnrohr BG, Vyse TJ, Rhodes B. The complement receptor 3 (CD11b/CD18) agonist Leukadherin-1 suppresses human innate inflammatory signalling. *Clin Exp Immunol* (2016) **185**:361–371. doi:10.1111/cei.12803

209. Faridi MH, Khan SQ, Zhao W, Lee HW, Altintas MM, Zhang K, Kumar V, Armstrong AR, Carmona-Rivera C, Dorschner JM, et al. CD11b activation suppresses TLR-dependent inflammation and autoimmunity in systemic lupus erythematosus. *J Clin Invest* (2017) **127**:1271–1283. doi:10.1172/JCI88442

210. Dickson CM, LeBlanc B, Edhi MM, Heffernan DS, Faridi MH, Gupta V, Cioffi WG, O’Brian X, Reichner JS. Leukadherin-1 ameliorates endothelial barrier damage mediated by neutrophils from critically ill patients. *J Intensive Care* (2018) **6**:1–10. doi:10.1038/s41575-019-0191-1

211. Schmid MC, Khan SQ, Kaneda MM, Pathria P, Shepard R, Louis TL, Anand S, Woo G, Leem C, Faridi MH, et al. Integrin CD11b activation drives anti-tumor innate immunity. *Nat Commun* (2018) **9**:1–14. doi:10.1038/s41467-018-07387-4

212. Yao X, Dong G, Zhu Y, Yan F, Zhang H, Ma Q, Fu X, Li X, Zhang QQ, Zhang J, et al. Leukadherin-1-Mediated activation of CD11b Inhibits LPS-Induced pro-inflammatory response in macrophages and protects mice against endotoxic shock by blocking LPS-TLR4 interaction. *Front Immunol* (2019) **10**:215. doi:10.3389/fimmu.2019.00215
